# Genetics and timing of sex determination in the East African cichlid fish *Astatotilapia burtoni*

**DOI:** 10.1186/s12863-014-0140-5

**Published:** 2014-12-14

**Authors:** Corina Heule, Carolin Göppert, Walter Salzburger, Astrid Böhne

**Affiliations:** Zoological Institute, University of Basel, Vesalgasse 1, 4051 Basel, Switzerland

**Keywords:** Sexual development, Cichlidae, Adaptive radiation, Speciation, Gene expression profiles

## Abstract

**Background:**

The factors determining sex are diverse in vertebrates and especially so in teleost fishes. Only a handful of master sex-determining genes have been identified, however great efforts have been undertaken to characterize the subsequent genetic network of sex differentiation in various organisms. East African cichlids offer an ideal model system to study the complexity of sexual development, since many different sex-determining mechanisms occur in closely related species of this fish family. Here, we investigated the sex-determining system and gene expression profiles during male development of *Astatotilapia burtoni*, a member of the rapidly radiating and exceptionally species-rich haplochromine lineage.

**Results:**

Crossing experiments with hormonally sex-reversed fish provided evidence for an XX-XY sex determination system in *A. burtoni*. Resultant all-male broods were used to assess gene expression patterns throughout development of a set of candidate genes, previously characterized in adult cichlids only.

**Conclusions:**

We could identify the onset of gonad sexual differentiation at 11–12 dpf. The expression profiles identified *wnt4B* and *wt1A* as the earliest gonad markers in *A. burtoni*. Furthermore we identified late testis genes (*cyp19a1A*, *gsdf*, *dmrt1* and *gata4*), and brain markers (*ctnnb1A*, *ctnnb1B*, dax1A, *foxl2*, *foxl3*, *nanos1A*, *nanos1B*, *rspo1*, *sf-1*, *sox9A* and *sox9B*).

**Electronic supplementary material:**

The online version of this article (doi:10.1186/s12863-014-0140-5) contains supplementary material, which is available to authorized users.

## Background

Sexual development encompasses sex determination and sex differentiation and can be viewed as a complex genetic network that is initiated by a sex-determining trigger mediating the expression of sex differentiation genes, which ultimately establish the male or female phenotype [1]. In teleost fishes, with over 25,000 species the largest vertebrate group, sex determination mechanisms are much more variable compared to other vertebrates [2]. So far, six master sex-determining genes have been identified in teleosts, namely *dmy/dmrt1bY* in *Oryzias latipes* and *O. curvinotus* [3,4], *gsdf*^*Y*^ in *O. luzonensis* [5], *sox3* in *O. dancena* [6], *amhy* in *Odontesthes hatcheri* [7], *amhr2* in *Takifugu rubripes* [8] and *sdY* in *Oncorhynchus mykiss* and several other salmonids [9,10]. In addition to this variation in the initial regulators, we and others could show recently that also the subsequent genetic steps of sex differentiation are not conserved in fishes, asking for further investigation of the mechanisms of sexual development in this group of animals [11,12].

**Figure 1:**
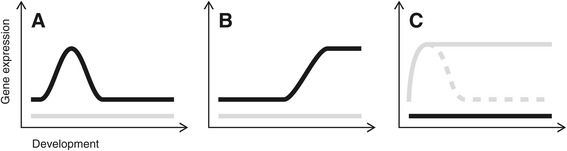
**Schematic expression patterns of sexual development genes.** The graphs show possible expression profiles post fertilization in the developing brain (grey line) and testis (black line). **(A)** Early testis genes (including sex-determining genes) are highly expressed before and/or at the onset of gonadal formation and subsequently down regulated. **(B)** Late testis genes are expressed later in development, mainly during the formation and maintenance of gonads. **(C)** Brain genes are higher expressed in the brains than in the testis, forming the brain and/or influencing the sexual development gene network via the action of hormones. Their expression can be maintained (continuous line) or decreased (dashed line) after the first increase in expression.

Master sex-determining genes are thought to be expressed early in development, thus marking the initial time point of the sexual development cascade. Their expression then either decreases directly after (comparable to the expression pattern shown in Figure 1A and in particular described for *dmy/dmrt1bY* in *O. latipes* [13]) or is maintained during the juvenile stage (as suggested for *amhy* [7] and *sdY* [9]). To the best of our knowledge, there is no example of a sex determination gene that is still highly expressed in adult fish. However, expression studies on several fish sex determination genes covering the development from embryo to adults are lacking, and in mammals, the sex-determining gene *sry* is expressed in adult testis of mouse and rat [14,15].

Sex differentiation genes, on the other hand, can act at different time points after their initiation until sexual maturity (i.e., until gonads are fully developed) or even afterwards, e.g., by being involved in gonad maintenance and function (Figure 1B and exemplified by *dmrt1* [16-18]).

Similarly to gene expression patterns in the gonads, sex differentiation genes can be expressed in the brain as part of the hypothalamus-pituitary-gonadal axis, and hence can -like gonad genes- follow one of the two patterns shown in Figure 1C.

In general, gonads are formed by the interplay of sexual development genes and the action of hormones [19-22]. This can be a rather plastic process, especially in fish, making it more difficult to classify sex differentiation genes according to their expression profiles and also questioning a separation between sex determination and differentiation [23].

Cichlid fishes, and the species flocks of cichlids in the East African Great lakes in particular, are an excellent model system in evolutionary biology, with hundreds of closely related species showing a high degree of diversity in morphology, behavior and ecology [24-27]. This diversity also seems to apply to sex determination systems, as evidenced by data suggesting that different mechanisms occur in cichlids including sex determination via environmental (temperature and pH) and genetic factors (single gene or polygenic actions), or a combination thereof [28-33]. The best-studied cichlid in terms of sexual development is the widely distributed and farmed Nile tilapia (*Oreochromis niloticus*), which has an XX-XY sex-determining system that can strongly be influenced by temperature [34]. There are two time windows (2–3 days post fertilization, dpf, and 10–20 dpf), in which temperature and steroid hormones can override genetic sex determination in the Nile tilapia, with the actual critical time period of gonad differentiation at 9 to 15 dpf [34 and references therein]. Studies of sexual development in the Nile tilapia encompass both, genetic and morphological data, and therefore make this species a good reference system.

Here, we focused on another cichlid species, *Astatotilapia burtoni,* which inhabits Lake Tanganyika, and its affluent rivers, and is a model system especially in behavioral but also genetic research (e.g., [35]). This sexually dimorphic species, in which males are larger and brightly colored whereas females are rather dull, belongs to the most derived and species-rich lineage of East African cichlids, the haplochromines. Like the Nile tilapia, *A. burtoni* is a maternal mouthbrooder; the female incubates the fertilized eggs in her buccal cavity at least until hatching. Because of different developmental pace, the sexual development of *A. burtoni* cannot be compared in exact (day to day) time steps to the Nile tilapia. Although Nile tilapia and *A. burtoni* embryos hatch approximately at the same age (5–6 dpf [36] and 4–7 dpf, [37], respectively), Nile tilapia embryos start free swimming earlier than *A. burtoni* embryos (12 and 14 dpf, respectively [36,37]) but become sexually mature later (at the age of 22–24 weeks [38] compared to 13–14 weeks in the here used *A. burtoni* strain, personal observation). Until now, the embryonic and juvenile development of *A. burtoni* has not been studied in detail. Even though *A. burtoni* is one of the five cichlid species with a sequenced genome [39], neither the sex-determining system nor the time window of sex determination have been characterized.

Based on the assumption that sex is determined genetically, we used a common approach to infer male or female heterogamety. We generated mono-sex fish groups over steroid hormone treatments via food and conducted crossing experiments. The resultant sex ratios point to an XX-XY sex-determining system in *A. burtoni*. Subsequent crossings were carried out to generate a YY-supermale to sire male-only offspring. Making use of candidate genes expressed in brain and gonad tissue of adult *A. burtoni* [11], we studied changes in gene expression throughout male sexual development. Without prior knowledge on the time window of actual sex determination in this species, we decided to investigate gene expression as early as possible starting at 7 dpf. We profiled expression of sexual development genes from 7–48 dpf using high throughput quantitative real-time polymerase chain reaction on single individuals. Most of the gene expression profiles corresponded to one of the following patterns: early testis genes, late testis genes and brain/head genes (Figure 1).

## Results

### Generating all-male broods in *A. burtoni*

**Figure 2:**
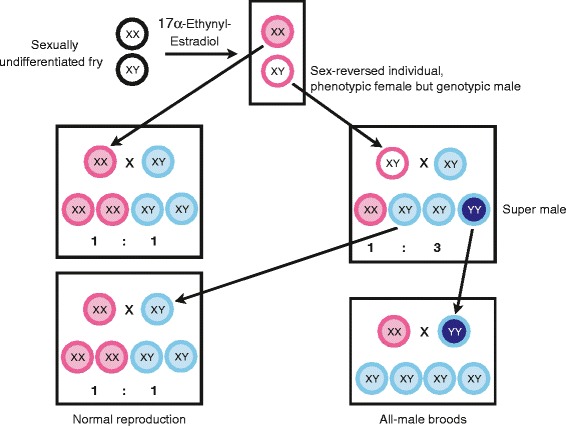
**Crossing scheme to obtain all-male broods from estrogen sex-reversed fish.** Sexually undifferentiated fry including both, XX- and XY-genotypes, were treated with estrogen resulting in only phenotypical females. These females – genetic females and sex-reversed genotypic males – were then crossed to untreated genotypic males. Crossing of XX-females to untreated males (left site) reflect the normal reproduction with a sex ratio of 1:1, with the corresponding female XX and male XY genotypes in the offspring. Crossing of sex-reversed XY-females to untreated XY-males led to a sex ratio of 1:3 with the genotypes XX (phenotype female), XY (phenotype male), YY (super male, phenotype male). These two types of phenotypically undistinguishable males were back-crossed to normal XX-females resulting again in either a 1:1 sex ratio (for XY-males) or in all-male broods (for the YY-male). Pink and blue outer circles denote phenotypic females and males, respectively.

Sexual development in fish is plastic and sex reversal can be induced in a variety of species even after reaching sexual maturity [40]. For these purposes, steroid hormones or hormone synthesis inhibitors can be administered over the surrounding water or via food supply. Here, we fed four *A. burtoni* broods with estrogen treated flake food during four weeks of development in order to obtain all-female broods. We started treatment at the earliest feeding point of this species, at around 14 dpf. This procedure has been carried out successfully in another cichlid species, the Nile tilapia (personal communication H D’Cotta), which starts feeding at around 12 dpf [36]. After treatment, we obtained 100% morphological females in all broods. These natural female and feminized fish were used for crossings with untreated, normal males. Among the offspring of these individual crossings, four broods showed a ~ 1 : 3 (female : male) sex ratio, whereas other crosses, likely derived from normal females, which can morphologically not be distinguished from sex-reversed individuals, had a sex ratio of approximately 1 : 1. This is a strong indication for an XY-XX system in *A. burtoni* (Figure 2)*.* Note that a ZZ-ZW female heterogametic sex determination system can be ruled out for *A. burtoni*, because sex-reversed ZZ females would have produced only males in the first generation of crossings, all of our crosses however contained at least 1/3 female offspring.

Crossings of sex-reversed XY fish (phenotypic females) with normal, XY-males should lead to the following types and proportion of offspring: one quarter of XX-females, two quarters of XY-males and one quarter of YY-males (super-males) (Figure 2). Note that, morphologically, the two types of males should be undistinguishable.

Subsequent crossings of all males of one of the broods with a 1:3 sex ratio to normal females revealed one male that only produced male offspring, suggesting that it is indeed a YY-male, lending further support to an XX/XY sex determination system in this species.

### Expression profiles of sexual development genes

We crossed the YY-super-male to XX-females to produce all-male broods, which we used to investigate expression patterns of sex differentiation genes during early male development. In similar experiments in the Nile tilapia, the spurious occurrence of females in the offspring of super-males has been reported [41]. To allow a potential detection of such spontaneously occurring phenotypic females in these broods, gene expression was measured in individual samples rather than pooling samples. To our knowledge, this is the first study that used a large number of individual samples in a dense sampling scheme for establishing the gene expression profiles of a set of candidate genes for sexual development (24 genes tested in 88 individuals sampled at 22 time points during a period of 40 days). Fish were dissected from the yolk and separated in head and trunk, as proxies for developing brain and gonad. Single organ dissection is not possible at these early stages of development, especially if gene expression is to be accessed on an individual basis. The chosen approach has already successfully been applied in other species [5,7,42-47].

The relative expression of a set of candidate genes, previously tested in brain and gonad tissue of adult cichlid fishes [11], plus one additional gene, *gsdf*, was profiled during male development. These genes are candidates for sex determination and differentiation as suggested by their described function in fish and tetrapods. This gene list includes, wherever existing, the two paralogous gene copies emerging from the fish-specific whole-genome duplication [48].

**Figure 3:**
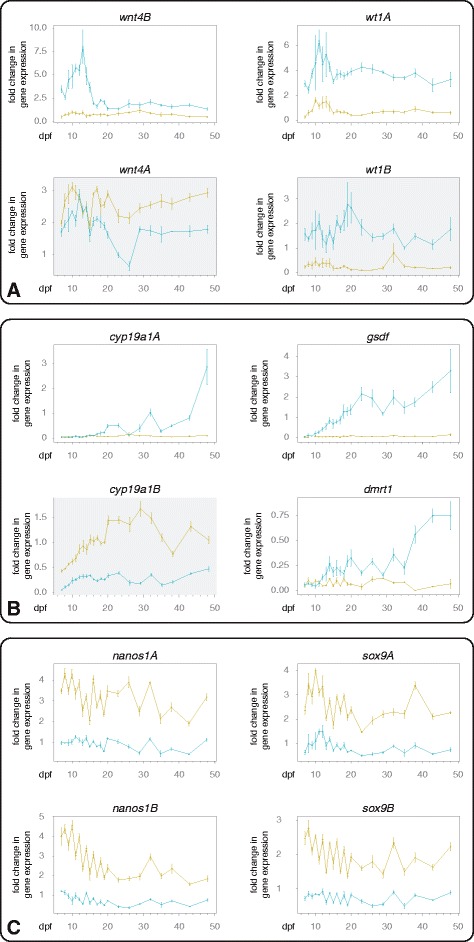
**Gene expression of sexual development genes in heads and trunks of developing male** ***A. burtoni*** **. (A)** *Wnt4B* and *wt1A* were the only detected early testis genes, here shown with their paralogous gene copies *wnt4A* and *wt1B* (grey background). **(B)** *Cyp19a1A*, *gsdf* and *dmrt1* are examples of late testis genes, *cyp19a1B* is the teleost specific paralog of *cyp19a1A* (grey background). **(C)** *Nanos1A*, *nanos1B*, *sox9A* and *sox9B* are examples for brain genes. Gene expression is shown as fold change (Livak) ± SE in heads (green) and trunks (blue) from 7 – 48 dpf using *rpl7* as reference gene and a juvenile tissue mix as reference tissue (see Additional file 3 for further details).

The brain and the gonads are the main tissues acting in sexual development. In addition, sexually dimorphic expression can be observed in the brain even earlier than in the gonad, a pattern already described in cichlids [43,49]. Samples were taken between 7 and 48 dpf, with a daily sampling at the beginning of the experiment (7 – 20 dpf) and then every third (during 20 – 38 dpf) and afterwards every fifth day (38 – 48 dpf,) as day-to-day changes are more prominent early in development [36]. We then used the Fluidigm system to test the expression of the 24 candidate genes. Gene expression was calculated as fold change in gene expression using the delta-delta-CT method [50], compared to expression in a juvenile tissue pool (Figure 3 and Additional file 1) or relative to the mean of the four biological replicates at the first sampling point at 7 dpf (Additional file 2). For each sampling point the fold change in gene expression in heads and trunks of four individuals was calculated. For details on sample sizes for each gene see Additional file 3.

The expression profile of a known testis-specific gene (*dmrt1*) in all tested trunks strongly suggests that all individuals were indeed males and that none of the offspring was a female. In addition, we raised fish that were not used for the gene expression experiment to adulthood/maturity and confirmed that all of them were males. We hence did not detect any occurrence of spurious females.

We investigated gene expression patterns according to the expression profiles explained in Figure 1 and compared expression between heads and trunks. Figure 3 shows the most prominent examples for the expression profiles early testis genes, late testis genes and (early) brain genes (for all expression profiles see Additional files 1 and 2). In the following, we describe the results in more detail.

### Testis and brain markers

From all 24 candidate genes, only *wnt4B* and *wt1A* are likely to represent early testis genes, i.e., showing a peak in expression early in development and in trunks only (Figure 3A, corresponding to the profile shown in Figure 1A). *Cyp19a1A*, *gsdf* and *dmrt1* appeared as late testis genes with an increase in trunk expression over time (Figure 3B, corresponding to the profile shown in Figure 1B). *Gata4* showed a similar increase in expression in trunks starting earlier as the other genes, around 15 dpf (see Additional file 1). In total, we detected 12 ‘brain’ genes (*ctnnb1A*, *ctnnb1B*, *cyp19a1B, dax1A*, *foxl2A/foxl2*, *foxl2B*, *nanos1A*, *nanos1B*, *rspo1*, *sf-1*, *sox9A* and *sox9B*). For illustration purposes, we show the results for both gene copies of *wnt4*, *wt1* and *cyp19a1* in Figure 3.

### *Wnt4A* and *wnt4B –* different fates for gene copies

*Wnt4A* showed higher expression levels in heads than in trunks, whereas *wnt4B* showed the opposite signature with a higher expression in trunks than in heads. Also in adult males, *wnt4A* is significantly higher expressed in brain compared to testis tissue [11]. In adult cichlids, there is a detectable difference in gene expression between the two paralogs of *wnt4*, with the A-copy being ovary- and the B-copy being testis-specific [11]. W*nt4B* was one of only two genes with the earliest peak of expression in trunks (7 – 15 dpf), resembling the pattern of a sex-determining gene.

### *Wt1A* and *wt1B* – testis genes with different temporal patterns

*Wt1A* and *wt1B* are both higher expressed in trunks than in heads throughout the experimental time period, which is congruent with the pattern observed in adult males of *A. burtoni* [11]. *Wt1A* is the second gene that showed an expression peak in trunks at the beginning of development (between 7 and 15 dpf) but in contrast to *wnt4B* at the same time point also an increase of expression in heads (Figure 3A).

### *Dmrt1* and *gsdf* - late testis genes possibly important for gonad maintenance

*Dmrt1* is known as *the* conserved vertebrate testis gene [51] and also shows testis-specificity in adult *A. burtoni* [11]. We found similar levels of gene expression in heads and trunks early in development (7 – 11 dpf) followed by an increase (12 – 48 dpf) in expression in trunks only, pointing to a later function in testis development (Figure 3B). In many of the head samples *dmrt1* expression could not be detected (see Additional file 3 for details), which is consistent with previous results in adult brains [11].

*Gsdf* (*gonadal soma-derived factor*) is a sexual development gene only existing in fish [52], which has received considerable attention recently. In the above-mentioned *O. luzonensis*, Y- and X-chromosome specific alleles have been identified for this gene (*gsdf*^*Y*^and *gsdf*^*X*^, respectively), with the former turning out to be the master sex determiner in this species [5]. In another species, the sablefish *Anoplopoma fimbria*, *gsdf* seems to be a strong candidate for the sex-determining locus, too [53]. Furthermore in medaka, *gsdf* expression has been implicated with early testicular differentiation [54].

In *A. burtoni* the expression profile of *gsdf* resembled that of *dmrt1*, with a constant increase of expression in trunks after a short time of low expression (7–10 dpf), and constant low expression in heads (Figure 3B). Just as for *dmrt1*, in some of the head samples, *gsdf* expression could not be detected (see Additional file 3 for details).

### The aromatases *cyp19a1A* and *cyp19a1B*

The expression pattern of the aromatase *cyp19a1A* in the heads remained similar over time whereas its expression in trunks increased constantly. The expression of *cyp19a1B* was always higher in heads than in trunks, with an increase in expression in both tissues during 7 – 11 dpf, followed by a stable period (12 – 43 dpf), and then the expression in trunks increased again (48 dpf). The expression pattern of *cyp19a1A* in adults of *A. burtoni* in brain and gonad tissue shows no difference, and the expression pattern of *cyp19a1B* shows a significant testis-specific over-expression [11]. In developing *A. burtoni* males, *cyp19a1A* seems to play a role in the gonads. The testis-specific expression of *cyp19a1B* seen in adults only becomes established after 48 dpf, with a start of rising expression detected in our experiments after 40 dpf.

### Markers of the developing brain

As mentioned above, we detected 12 ‘brain’ genes. The strongest differences in expression between heads and trunks, and hence likely representing brain up-regulated genes, were found for *nanos1A*, *nanos1B*, *sox9A* and *sox9B* (Figure 3C)*.* This is consistent with the expression patterns seen in adult males of *A. burtoni*, where a significantly higher expression in brain tissue than in the testis has been found [11]. The expression level of *nanos1B* in heads was highest at 7 dpf and then decreased (comparable to Figure 1C, dashed line). *Sox9*, similar to *dmrt1*, is considered a prominent example for a gene generally involved in testis formation and function [55,56]. However, this does not seem to be the case in developing and adult *A. burtoni*.

### Investigation of the early testis markers: Sequence and promoter analysis of *wnt4B* and *wt1A*

**Figure 4:**
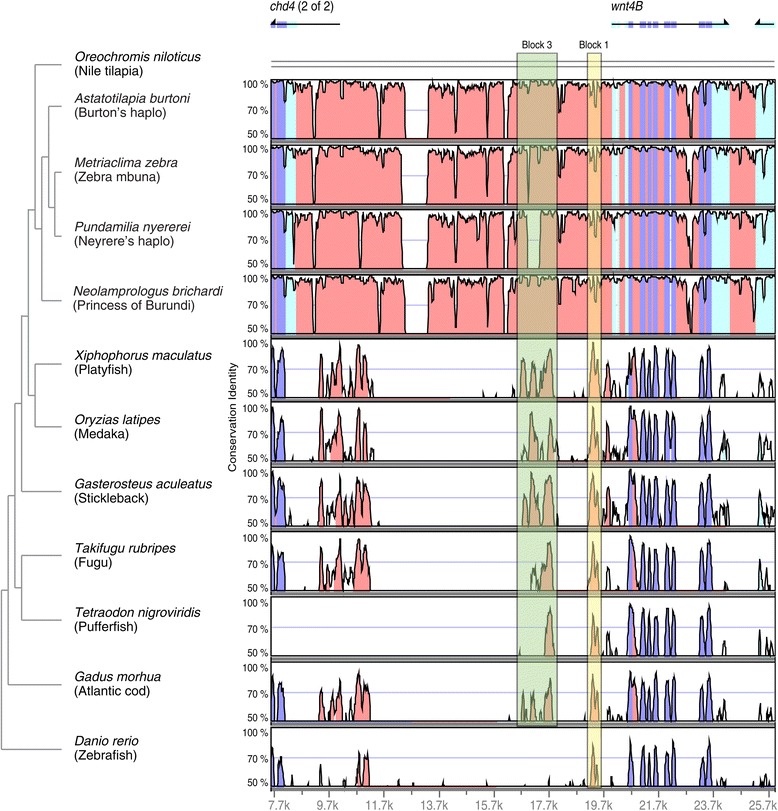
**Comparison of the** ***wnt4B*** **upstream region.** Shuffle-LAGAN Vista plots [57,58] for *wnt4B* and its 5' adjacent gene *chd4*. Peaks indicate conservation identity of sequences above 50% across the tested species. Blue stands for coding and pink for noncoding regions, respectively. Light blue regions represent UTRs. Yellow block 1 and green block 2 were investigated in the process of transcription factor binding site analysis (Table 1).

**Figure 5:**
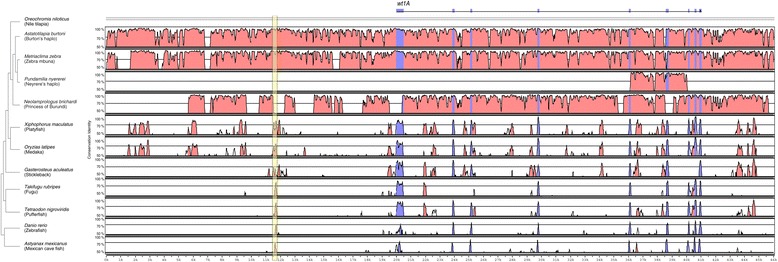
**Comparison of the** ***wt1A*** **upstream region.** Shuffle-LAGAN Vista plots [57,58] for *wt1A*. Peaks indicate conservation identity of sequences above 50% across the tested species. Blue stands for coding and pink for noncoding regions, respectively. The yellow block was investigated in the process of transcription factor binding site analysis (Table 2).

As the *wnt4B* and *wt1A* expression showed a peak early in development (7 – 15 dpf) and then decreased to a constantly low level, thus mimicking the expression of a potential sex determination gene, we decided to investigate these genes’ sequences in detail in *A. burtoni*. For *wnt4B,* we sequenced the entire genic region, whereas for *wt1A* we focused on the coding region only, due to the large size of the region (~ 20 kb). A sequence comparison of the coding region of males and females did not show any allelic differences between the sexes for both genes. Also the intronic sequences of *wnt4B* did not show any sex-specific differences. However, gene expression could still be differently regulated due to sex-specific changes in the promoter region of the genes. To identify the potential promoter regions of *wnt4B* and *wt1A* we compared the upstream sequences of the two genes in the accessible teleost fish genomes using Vista plots of nucleotide similarity [57,58] (Figures 4 and 5). The 5’ neighboring gene to *wnt4B* is *chd4b*, which is located ~13 kb upstream. We created VistaPlots comprising this entire region. The next annotated gene 5' of *wt1A* is more than 50 kb upstream. We thus decided to focus our analysis on the region 20 kb upstream to *wt1A*.

In an additional step, after *in silico* definition of a core conserved upstream region of *wnt4B* (see colored blocks in Figure 4), we sequenced ~ 7 kb of this promoter in *A. burtoni* males and females of our lab strain. We also obtained ~ 4 kb upstream sequence for *wt1A*. Again, no differences between the sexes were found in the upstream regions of *wnt4B* and *wt1A.* For *wt1A* we detected two alleles with one of them having a 223 bp deletion compared to the reference genome. However, neither the deletion nor any other detected heterozygous site segregated with sex.

### Transcription factor binding-sites in *wnt4B* and *wt1A* potential promoters

To identify genes regulating *wnt4B* and *wt1A* expression and, thereby, possibly being more upstream in the sex-determining cascade, we performed a transcription factor binding-site analysis of the two conserved regions in *wnt4B* (blocks 1 and 2 in Figure 4) and the one conserved region in *wt1A* (yellow block in Figure 5) using MatInspector. We focused on transcription factors with a described function in gonads, germ cells, brain and/or central nervous system and compared the putative binding sites of *A. burtoni* with the ones present in all other available fish genomes. Tables 1 and 2 show all putative binding-sites detected in the *A. burtoni* sequence and indicate, in which other species these sites have been detected (for a complete table with all putative transcription factor binding-sites including non-conserved sites in all tested species, see Additional files 4 and 5).

**Table 1:** **Predicted transcription factor binding sites in the** ***wnt4B*** **promoter region of** ***A. burtoni***

**Block 1**								**Block 2**						
***A. burtoni***	***X. maculatus***	***O. latipes***	***G. aculeatus***	***T. rubripes***	***T. nigroviridis***	***G. morhua***	***D. rerio***	***A. burtoni***	***X. maculatus***	***O. latipes***	***G. aculeatus***	***T. rubripes***	***T. nigroviridis***	***G. morhua***
AR								**AR**			x			
**E4BP4**	x		x	x	x	x	x	**Creb**	x	x	x	x	x	x
**Ets1**							x	**Creb1**	x	x				
**Foxa-1 and 2**	x	x	x		x	x	x	Dbp						
**Foxk2**	x			x			x	**Dec2**	x	x	x			x
Gsh2								**Egr1**		x	x			
Helt								**ESRRA**	x		x	x		x
Isl1								**Evi1**				x	x	x
**Meis1**	x	x	x	x	x	x	x	**FAC1**	x	x	x	x	x	x
**Myt1**	x	x	x	x		x	x	FoxC1						
Pax6								**Hmx2**					x	x
**Plag1**	x	x	x		x	x		**Hmx3**	x			x		
Satb1								**Hre**	x		x		x	x
**Sox6**	x	x	x			x		Ir1						
**Spi1**		x	x	x	x	x	x	**Irx5**	x	x				
**Tef**	x		x	x	x	x	x	**Mef3**	x					
								**Meis1**			x	x	x	x
								**MEL1**	x		x		x	x
								**Myf5**						x
								**Myf6**		x		x	x	x
								**Nanog**	x	x		x	x	x
								**NF1**	x	x	x	x		x
								**Nkx2-5**	x		x			x
								**Nkx6-1**						x
								**Nur-Family**			x	x		
								**Pax4**	x		x			x
								**Plag1**	x	x	x	x	x	x
								**Pou5f1**	x	x	x			x
								**Rfx4**	x		x			
								**Rfx7**	x					
								**SF1**			x			x
								**Sox3**	x	x	x	x	x	x
								**Sox30**		x			x	
								**Sox6**	x	x	x	x	x	x
								Ttf1						
								**Wt1**	x	x	x	x		x
								Ybx1						
								**Zfp67**	x	x				x
								**Zic2**					x	x

Blocks correspond to the green and yellow regions in Figure 4. Bold binding sites are shared with at least one other species. "x" denotes the detection of the binding site in the respective species.

**Table 2:** **Predicted transcription factor binding-sites in the** ***wt1A*** **promoter region of** ***A. burtoni***

***A. burtoni***	***M. zebra***	***N. brichardi***	***O. niloticus***	***X. maculatus***	***O. latipes***	***G. aculeatus***	***T. rubripes***	***T. nigroviridis***	***D. rerio***	***A. mexicanus***
**AP1**	x		x					x		
**ATF1**	x			x						
**ATF6**	x	x	x							
**Atoh1**	x		x	x	x				x	x
**Barx1**	x		x	x						
**Bcl6b**	x	x	x							
**Creb**	x	x	x		x	x				
**Creb1**	x		x	x						
**Dlx2**	x		x						x	
**Dlx3**	x		x	x					x	
**Dmrt2**	x		x						x	
**dre**	x		x							
**E2a**	x		x	x	x		x	x	x	x
**Elf3**	x		x	x	x		x			
**eng1a**	x		x							
**eng2a**	x		x	x						
**Evi1**	x	x	x	x					x	
**FAC1**	x		x							
**Fkhrl1**	x	x	x							
**Foxa1**	x		x							
**Foxp1**	x		x						x	
**foxp2**	x		x							
**gli3**	x		x	x						
**gr**	x		x	x		x				
**Gsh2**	x		x	x					x	
**Hif1**	x	x	x							
**hlf**	x									
**HOX/PBX binding sites**	x		x				x			
**hoxb9**	x		x	x						
**ISL LIM homeobox 2**	x		x	x					x	
**Isx**	x		x	x						
**lhx2b**	x		x							
**Meis1**	x	x	x	x	x			x		x
**Meis1b and Hoxa9 heterodimeric complexes**	x	x	x						x	
**MEL1**	x	x	x		x			x	x	x
**Myf5**	x		x				x			
**MyoD**	x		x							
**Nk2-3**	x		x			x	x		x	
**Nkx2-5**	x	x	x	x	x				x	
**Nkx2-9**	x		x			x	x		x	
**Nkx5-1**	x		x	x					x	x
**Nobox**	x		x	x					x	
**nr2c1**	x		x	x	x		x		x	
**nrf2**	x		x							
**nrsf**	x		x	x					x	
**Pax6**	x	x	x	x	x		x	x	x	x
**pce1**	x		x	x					x	
**Plag1**	x		x		x		x			
**Pou3f2**	x								x	
**S8**	x		x	x					x	
**six1b**	x		x							
**Sox6**	x	x	x	x		x	x	x		
**Tax/CREB complex**	x		x						x	
**Tgif**	x		x	x					x	
**Zfp67**	x		x	x	x		x			

Shown are binding sites in the conserved region marked in yellow in Figure 5. Bold binding sites are shared with at least one other species. "x" denotes the detection of the binding site in the respective species.

Interestingly, we identified several conserved binding sites for transcription factors that have been implicated with sexual development before. For *wnt4B* we found that six out of seven species show a conserved putative binding site for Wt1 in block 2 (Table 1). This fits well with our own expression data (Figure 3) as well as other studies in fish [59,60], which support an involvement of *wt1A* in early testis formation. Other promising upstream candidates of *wnt4B* are Sox30 and the androgen receptor (AR). Sox30 is expressed specifically in gonads of the Nile tilapia, with one isoform being even limited to the developing testis [61]. The androgen receptor can bind testosterone and dihydrotestosterone and thereby plays an important role in controlling male development [62]. Interestingly, *ar* is higher expressed in brains of dominant *A. burtoni* males than in subordinate males [63]. In the developing gonads of the Nile tilapia the expression levels of *ar* in males and females are similar [17].

Remarkably, we found putative transcription factor binding sites for two of our candidate genes: *wt1* (discussed above and Figure 3A) and *sf-1* (Additional file 1). However, the expression pattern of *sf-1* in developing testis (expression in trunks) does not support its putative role as a direct regulator of *wnt4B*, as it was expressed at low levels during the experimental time period (Additional file 1). The expression profiles in heads, on the other hand, showed high expression at the beginning (7 – 12 dpf), with a constant decrease afterwards (as in Figure 1C, dashed line; and Additional file 1). *Sf-1* might thus be an example of an early brain gene influencing sexual development via other factors than *wnt4B*.

In contrast to *wnt4B*, we could identify only one small conserved block upstream of *wt1A*. We did not find a binding-site for any of our candidate genes or an obvious transcription factor already known to play a role in sexual development or any binding site only present in *A. burtoni* in that block. However, we found a broad range of neuronal transcription factors and binding sites for members of the dm-domain family, here *dmrt2*, which might have a female sex-specific role in adult cichlids [64]. As for *wnt4B*, we also found a binding site for a Sox-family member, here Sox6.

Interestingly, we found binding sites for several members of the forkhead transcription factor family (Foxa1, Foxp1, Fkhrl1 alias Foxo3 and Foxp1), which are known as regulators of development and reproduction. Together with *foxl2* and *foxl3*, they were also among the candidate genes in our expression assay.

## Discussion

Here we provide first experimental proof for a male sex-determining (XX-XY) system in the haplochromine cichlid *Astatotilapia burtoni,* making use of hormonal sex-reversal and the subsequent generation of mono-sex broods. Offspring from male-only broods were investigated for gene expression patterns to define the window of sex determination in *A. burtoni*, which seems to take place at 11–12 dpf.

Throughout larval development, we decided to investigate gene expression in whole heads and trunks, including also other tissues than brains and gonads. Similar studies have been conducted in the Nile tilapia, which revealed that expression of sexual development genes in brains and testis is comparable to the one in heads and trunks, respectively [42,43].

We chose this approach in order to assess the individual gene expression level rather than pooling samples. Furthermore, the timing of morphological development, especially of gonads but also brain structures, is unknown in *A. burtoni* and no marker of gonad differentiation is available for this species, making an early single tissue dissection physiologically and technically impossible. By using whole trunks we made sure that we did have testis tissue in our samples starting from the onset of gonad formation. Another reason why it was important to test gene expression on an individual level is the possible occurrence of spurious XY-females in offspring derived from super-males, which has been described for other cichlids [41,65]. Furthermore, the sex determining gene(s) are not yet identified in *A. burtoni* and additional minor factors influencing sexual fate (environmental or genetic) cannot be ruled out.

After careful inspection of all raw and analyzed data, we did not find any evidence of females in the broods sired by the super-male, i.e., there was no individual with opposing expression patterns at a given sampling point. Especially the expression of the conserved testis factor *dmrt1* in the trunks is a good indicator for male gonad functioning, which is also evidenced by similar profiles in the Nile tilapia (increase in expression of *dmrt1* in testis [17]). A developing ovary would likely have contradicted this trend in gene expression.

Concerning the heads, we cannot rule out the possibility that the expression in other tissues than the brain is picked up by our experiment. For example, if the expression level of a gene is higher in eyes than in testis and higher in testis than in brains (corresponding to: “eyes > testis > brains”), then the overall head expression would be higher compared to trunks (and hence lead to the wrong classification into a brain gene). Having a closer look at the 12 "brain genes" identified by our approach, they either still show a higher expression in the brain than the testis in adult *A. burtoni* (eight genes) or have the reversed expression pattern in adults (four genes) [11]. Thus, the expression levels that we measured in the heads for these four genes (*dax1A*, *foxl2B*, *sf1* and *cyp19a1B*) might not be truly brain specific. Alternatively, the expression pattern may change later in development with an up-regulation in testis and/or down regulation in brain. We think that the latter is more likely, since *foxl2B*, *sf1* and *cyp19a1B* indeed showed a late increase in trunk/testis expression in our experiment, which might further increase beyond the period tested here.

Comparing between gene expression patterns within our experiment, we can show, once more, that paralogous gene copies derived from the fish specific whole genome duplication can evolve different functions, reflected by differences in tissue specificity. In our dataset this is true for *wnt4A* and *B* and *cyp19a1A* and *B*, with each of them having one copy being over-expressed in the heads and one in the trunks. However, we also observed a retention of the same (and hence probably ancestral) expression pattern in both gene copies, for example with very similar expression patterns for *nanos1A* and *B* and *sox9A* and *B*, which is also true in the adult stage [11].

Our main goal was to identify genetic markers for the time window of sex determination in *A. burtoni*. This critical time period, in which the decision if the bipotential embryonic gonad develops towards ovaries or testes is made, has so far been characterized in only one cichlid, the Nile tilapia, where it takes place at 9 to 15 dpf [17,34]. The trunk expression peaks of *wt1A* and *wnt4B* at 11 and 12 dpf suggested that also in *A. burtoni* the time window of sex determination takes place early in development, before any major signs of differentiated gonads become visible. In addition, the narrowness of the expression peak indicated that this time window is rather short. Note that our initial hormone treatment roughly started at the same time point likely accounting for the successful 100% sex reversal.

From the two genes with this early expression peak, especially *wnt4* received some attention in the research of sex determination. Female up-regulation or male down-regulation of *wnt4* expression have been described to be important for promoting ovarian development and function in mammals [66-68]. Also in the developing male gonad *wnt4* is needed for Sertoli cell differentiation, a crucial step for testis determination [69]. Still, data from teleost fish are largely lacking for *wnt4* and especially for the two teleost paralogs.

*Wt1* plays a role in testis differentiation and sex determination in mammals [70,71]. In the medaka, both genes, *wt1a* and *b,* are important for primordial germ cell maintenance, a crucial regulatory mechanism in gonad differentiation in fish [72]. In the Nile tilapia, *wt1a* is up-regulated in the developing male gonad [59]. Hence, *wt1* might act early in gonad differentiation also in other species.

Our sequence analysis of coding and promoter sequence of *wnt4B* and *wt1A* did not reveal any nucleotide difference associated with sex and thus ruled out the two genes as initial genetic regulator of sex determination in *A. burtoni*. However, it is very likely that they represent one of the first members of the sex determination network to be activated during the critical time point of sex determination. Interestingly, the promoter sequence of *wnt4B* contains a potential binding site for *wt1*, meaning that the two genes might functionally interact. Our promoter analysis further suggested that the *androgen receptor* (*ar*), *steroidogenic factor 1* (*sf1*) and *sox3*, three genes with a well-described function in male specific processes [70,73], might regulate *wnt4B* expression. Note that *ar* has two predicted binding-sites in the *wnt4B* promoter, with one being species-specific to *A. burtoni,* and that *sox3* has been co-opted as a master sex-determining gene in another fish species [6]. We did not detect any such obvious candidate among the possible transcriptional regulators of *wt1A*.

## Conclusion

In this study, we investigated the expression profiles of sexual development genes in the East African cichlid fish *Astatotilapia burtoni* during early male development. Based on hormonal treatment and subsequent crossing experiments we provided evidence that a male master determiner defines sex in *A. burtoni*. We identified early testis genes, late testis genes and male brain genes (Figures 1 and 3). The earliest testis markers *wnt4B* and *wt1A* were investigated in more detail, as they are strong candidates for the role of the sex-determining gene in *A. burtoni*, due to their expression pattern. Genomic sequences of males and females showed no differences, neither in the coding nor in their promoter region, ruling them out as an initial genetic male determiner. Nonetheless, we suggest that both have an important function early in the sexual development cascade and might even be one of the first targets of the still unknown sex determination factor. A transcription factor binding site analysis revealed possible candidates for master regulators of sexual development in *A. burtoni* such as *sox30*, *ar* and *sf-1*. Future investigations of these candidates, including sequence and expression analyses, together with similar gene expression experiments in female *A. burtoni* should shed more light on the complex cascade of sexual development to finally uncover the master sex-determining gene in this model cichlid species.

## Methods

All experiments involving animals were performed in accordance with public regulations under the permits no. 2317 and no. 2620 issued by the cantonal veterinary office of the canton Basel-Stadt (Switzerland).

### Estrogen treatment

Animals used in this study were derived from a lab strain of the species *A. burtoni*, an East African cichlid fish from Lake Tanganyika and its surrounding affluent rivers, reared at the fish facility of the Zoological Institute of the University of Basel at 24°C with a 12 hours dark–light cycle.

We treated four clutches of *A. burtoni* with 17α-EthynylEstradiol (E-4876, Sigma) for feminization (protocol kindly provided by H. D’Cotta; see also [1]). 15 mg 17α-EthynylEstradiol were dissolved in 100 ml of 100% ethanol, poured onto 100 g flake food (sera vipan®) and dried at 37°C. From 14 dpf (which is the date when the first fish in the clutches started feeding after the yolk had been absorbed) fish were fed three times a day during four weeks with the hormone treated food. Feeding with 17α-EthynylEstradiol treated food resulted in 100% morphological females in all broods. Amongst these morphological females, we expected (assuming an XX-XY sex determination system) that roughly half of the individuals would have an XX (female) and the other half an XY (male) genotype. Treated fish were subsequently crossed with untreated, normal males. Among the offspring of these individual crossings, several broods showed a 1: 3 (female : male) sex ratio indicative of an XY genotype of the mother (feminized genetic male). Of these crosses, all male offspring was further crossed to normal females. One of these crosses resulted in all male offspring, suggesting that the father was a YY-supermale. For an overview of the crossing design see Figure 2.

### Tissue sampling

The potential YY-male resulting from the above mentioned experiment was crossed to untreated females of the lab strain to produce all-male broods. The resulting eggs were collected within an hour after fertilization from the female’s mouth and incubated in an Erlenmeyer at 24°C with constant airflow in a 12 hours dark–light cycle. Four individuals were sampled at each of the sampling points at the following days post fertilization: 7, 8, 9, 10, 11, 12, 13, 14, 15, 16, 17, 18, 19, 20, 23, 26, 29, 32, 35, 38, 43 and 48. This sampling scheme, with a denser sampling early in development, was chosen because development progresses faster in early stages compared to later stages [36]. Eight clutches were needed to obtain a total of 88 fish. Individuals were photographed for length measurements with a Leica DFC 310 FX (Leica Microsystems). At these early developmental time points, the sampled fish are too small (~ 5 mm standard length) to dissect single organs. To guarantee sufficient RNA material, we thus separated embryos into heads and trunks as proxies for developing brain and gonad tissue, an approach widely used in other fish species [5,7,42-47]. Dissected tissues were stored in Trizol at −80°C until further proceeding.

### RNA extraction, DNase treatment and cDNA synthesis

Thawed samples were homogenized using a FastPrep®24 beat beater (MP Biomedicals Europe). Total RNA was extracted following the Trizol protocol. RNA quality and concentration were measured using a NanoDrop 1000 spectrophotometer (ThermoScientific). The RNA was stored at −80°C until further use. RNA samples were treated with DNA-*free*™ Kit (LifeTechnologies) as recommended by the manufacturer. DNase-treated RNA was reverse transcribed using the High Capacity RNA-to-cDNA™ Kit (LifeTechnologies) according to the manufacturer’s protocol and diluted to a concentration of 5 ng/μl of cDNA for further procedure.

### qRT-PCR expression experiments

In addition to 24 primers (23 candidate genes and *rpl7* as a reference gene) described in [11], and to the primers for *ef1a* and *rpsA3* (used as further reference genes, described in [74]) a primer pair for *gsdf* (as a candidate gene, Forward 5’- CCACCATGGCCTTTGCATTC -3’ and Reverse 5’- TCACAGGTGCCAAGGTGAGT -3’) was designed and validated for *A. burtoni* following the procedure described in [11] *Rpl7*, *rpsA3* and *ef1a* were tested as possible reference genes. *RpsA3* and *rpl7* showed high stability over all samples (whereas *ef1a* showed slightly more variation). Subsequently, *rpl7* was chosen as a reference gene in the analysis of the qRT-PCR experiments.

Prior to the qRT-PCR experiment, a specific target amplification (multiplex-amplification to increase the amount of targets of interest) was carried out as follows: 2.6 μl TaqMan PreAmp Master Mix (LifeTechnologies), 1.3 μl of a 200 nM mix of all primer pairs and 1.3 μl cDNA were pre-amplified in a thermo cycler (LifeTechnologies) (cycling conditions: 1 × 95°C for 10 minutes, 14 × 95°C for 15 seconds and 58°C for 4 minutes) and diluted 1 : 5 with Low EDTA buffer. The sample premix [2.5 μl TaqMan Gene Expression Mastermix (LifeTechnologies), 0.25 μl DNA Binding Dye Loading Reagent (Fluidigm), 0.25 μl Eva Green (Biotium), 0.75 μl Low EDTA buffer, 1.25 μl of cDNA] and the Assay mix [2.5 μl Assay Loading Reagent (Fluidigm), 0.25 μl Low EDTA buffer, 2.25 μl of 20 μM primer pair] were pipetted on a primed 96 × 96 chip and the plate was loaded in the IFC controller both according to Fluidigm protocols. Expression profiles of the candidate genes in heads and trunks of *A. burtoni* were measured using a Fluidigm BioMark™ assay (HD Systems) at the Genetic Diversity Centre (GDC) of the ETH Zurich with the following cycling conditions: 95°C for 10 minutes, 40 cycles of 95°C for 15 seconds and 58°C for 1 minute. All reactions were followed by a melt curve step to ensure primer specificity and detect possible erroneous amplification. The experiment included three technical replicates of all samples and four biological replicates of all the juvenile samples. Expression data was first analyzed using the Fluidigm Real-Time PCR analysis software to detect technical outliers and for the inspection of melt curves. As outliers we identified samples that showed a deviation from the other samples over all genes, what could easily be seen in the heat map generated by the software. This can happen if an integrated fluidic circuit on the Fuidigm system is blocked by an air bubble. The fold change in expression of the candidate genes in the samples was then calculated with the delta-delta-CT method [50] using custom R scripts. For normalization, the CT values of the reference gene *rpl7* and the mean CT value of a juvenile tissue mix were used. In an additional analysis the fold change was calculated and plotted relative to the mean of the four technical replicates at the first sampling point at 7 dpf (Additional file 2).

### *Wnt4B* and *wt1A* sequencing

DNA from adult males and females of *A. burtoni* labstrain individuals was extracted from fin clip samples by applying a Proteinase K digestion followed by sodium chloride extraction and ethanol precipitation as described in [75]. To sequence the coding and promoter region of *wnt4B*, nine primer pairs (one of them with two different reverse primers) were designed based on the *A. burtoni* genome [39] using GenScript*.* The genomic region of *wt1a* spans more than 20 kb in the Nile tilapia genome (over www.ensembl.org) here used as reference for annotation of the *wt1a* coding sequence in the non-annotated *A. burtoni* genome. We thus decided to focus on the coding region for sequencing and constructed primer pairs to amplify each of the nine exons. To sequence the potential promoter region of *wt1A*, six additional primer pairs were constructed covering ~ 4 kb upstream of *wt1a*. The adjacent annotated gene, *depdc7*, is located ~55 kb upstream of *wt1A* in the Nile tilapia genome. PCR reactions were carried out on nine individuals per sex for *wnt4A* and eight individuals per sex for *wt1A* using REDTaq DNA Polymerase (Sigma-Aldrich) and Phusion Master Mix (New England Biolabs) (for primer sequences and cycling conditions see Additional file 6). PCR products were visualized with GELRed (Biotium) on 1.5% agarose gels. Fragments were sequenced on a 3130xl capillary sequencer (Applied Biosystems) and alignments were performed with CodonCodeAligner (CodonCode Corporation), manually inspected and compared to the corresponding region in the *A. burtoni* genome.

### *Wnt4B* and *wt1A* promoter analysis

Promoter analysis was carried out on the upstream regions of *wnt4B* and the *wt1A* sequences of all the available teleost genomes over www.ensembl.org (release 62) and on the cichlid genome sequences of *A. burtoni*, *Neolamprologus brichardi*, *Orechromis niloticus*, *Pundamilia nyererei* and *Metriaclima zebra* [39]. For *wnt4B* we extracted ~13 kb upstream region until it's next neighboring gene, *chd4*. For *wt1A* we analyzed ~20 kb upstream sequence. Alignments were done with mVISTA [57,58] using Shuffle-LAGAN as alignment algorithm. The Nile tilapia sequence was used as a reference. Putative transcription factor binding sites for *A. burtoni* and the sequenced teleost genomes were identified using MatInspecor (Genomatix Software GmbH). We selected transcription factors that showed a matrix similarity > 0.9 and that belonged to one of the following categories: testis, ovary, germ cell, brain and/or central nervous system. Abbreviated names of transcription factors were taken from Genbank. Tables 1 and 2 show all factors detected in *A. burtoni* and their conservation in the other investigated teleost genomes (indicated by an "x" in Table 1). The complete list with all detected binding sites in all species is shown in Additional files 4 and 5.

## Additional files

Additional file 1:
**Expression data of additional sexual development genes during development of** ***A. burtoni*** **.** Gene expression as fold change (Livak) ± SE in heads (light green) and trunks (dark blue) from 7 – 48 dpf using *rpl7* as reference gene and a juvenile tissue mix as reference tissue. For details on sample size see Additional file 3. Additional file 2:
**Expression data of all candidate genes during development of** ***A. burtoni*** **.** Gene expression as fold change (Livak) ± SE in heads and trunks relative to the first sampling point at 7 dpf. For details on sample size see Additional file 3. Additional file 3:
**Sample sizes for qRT-PCR experiment if other than four.** Sample size of trunks at 8, 10 and 11 dpf is three for all genes (and two for *wnt4B*) and therefore not depicted here. Besides *sf-1* (trunk tissue of three individuals at 12 and 13 dpf) and *gata4* (head tissue of three individuals at 38 dpf) all the missing data can be accounted to not detectable expression of *dmrt1* and *gsdf* in heads. Additional file 4:
**Putative transcription factor binding sites in the conserved promoter regions (block 1 and block 2 as in Figure** 4 **) of** ***wnt4B*** **in teleost genomes.** We chose transcription factors with a Matrix similarity > 0.9 and described in the tissues testis, ovary, germ cells, brain and/or central nervous system. Included species are *A. burtoni*, *Xiphophorus maculatus*, *Oryzias latipes*, *Gasterosteus aculeatus*, *Takifugu rubripes*, *Tetraodon nigroviridis*, *Gadus morhua* and *Danio rerio* for block 1 and without *Danio rerio* for block 2. Abbreviated names of transcription factors were taken from Genbank. Additional file 5:
**Putative transcription factor binding sites in the conserved promoter region (yellow block in Figure** 5 **) within 20 kb upstream of** ***wt1A*** **in teleost genomes.** We chose transcription factors with a Matrix similarity > 0.9 and described in the tissues testis, ovary, germ cells, brain and/or central nervous system. Included species are *A. burtoni*, *Metriaclima zebra*, *Neolamprologus brichardi*, *Oreochromis niloticus*, *Xiphophorus maculatus*, *Oryzias latipes, Gasterosteus aculeatus*, *Takifugu rubripes*, *Tetraodon nigroviridis*, *Danio rerio* and *Astyanax mexicanus.* Abbreviated names of transcription factors were taken from Genbank. Additional file 6:
**Primer sequences and cycling conditions used for sequencing of** ***wnt4B*** **and** ***wt1A*** **coding and promoter sequence.** For amplicon six of *wnt4B* a second reverse primer (reverse 6_2) was designed closer towards forward 6 to ensure complete sequencing of this DNA stretch.

## Abbreviations

CT: Threshold cycle
dpf: days post fertilization
qRT-PCR: quantitative real-time polymerase chain reaction
RT: Room temperature
UTR: Untranslated region
