# Supplementary material for: Genetics and timing of sex determination in the East African cichlid fish Astatotilapia burtoni
Source: BMC Genet. 2014 Dec 14;15:140. doi: 10.1186/s12863-014-0140-5 (PMC4278230; doi:10.1186/s12863-014-0140-5)
Supplement: Additional file 1: — Expression data of additional sexual development genes during development of A. burtoni . Gene expression as fold change (Livak) ± SE in heads (light green) and trunks (dark blue) from 7 – 48 dpf using rpl7 as reference gene and a juvenile tissue mix as reference tissue. For details on sample size see Additional file 3. [file 12863_2014_140_MOESM1_ESM.pdf]

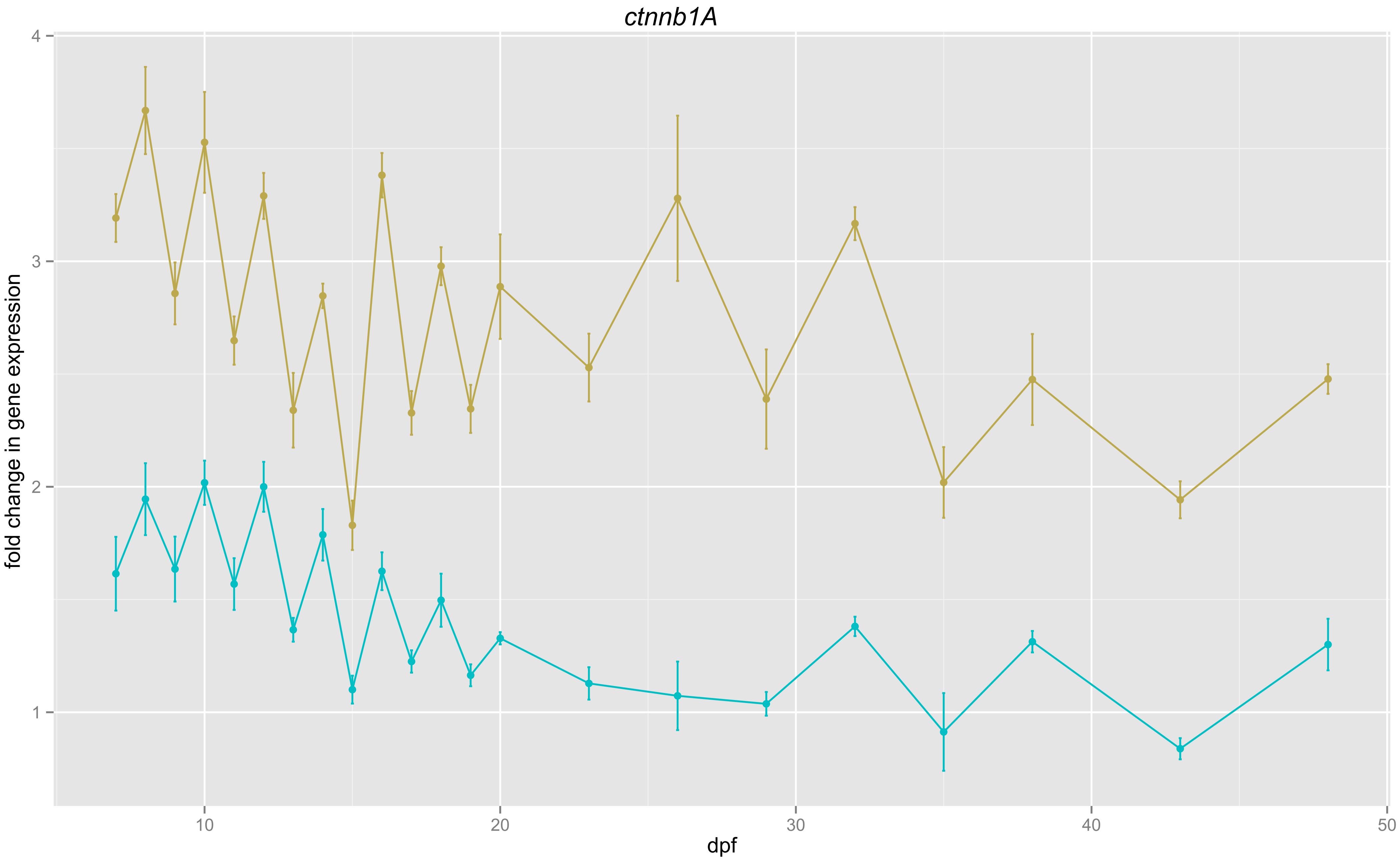
ctnnb1A
fold change in gene expression
dpf
1
2
3
4
10
20
30
40
50

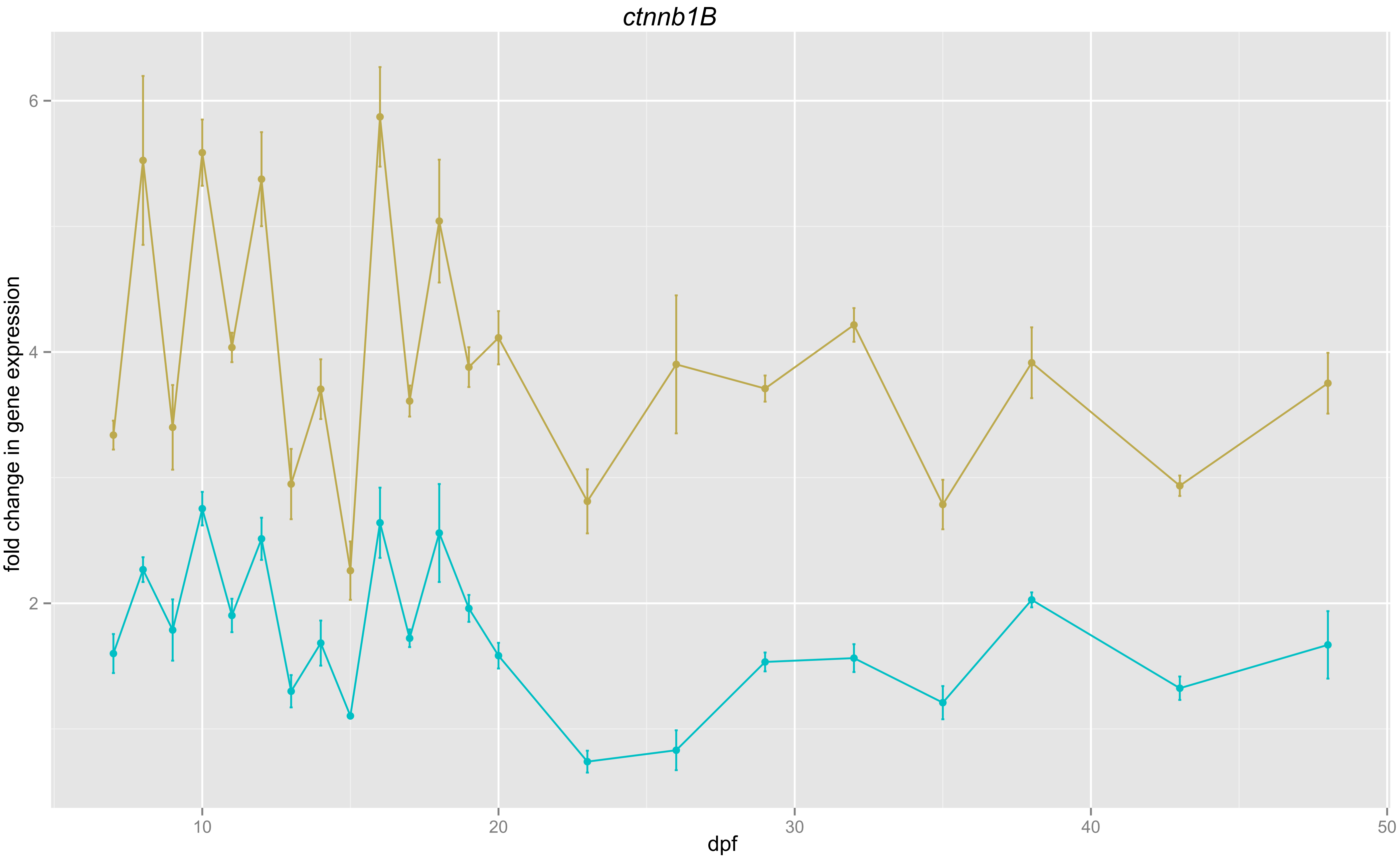

ctnnb1B
6
4
2
10
20
30
40
50
dpf
fold change in gene expression

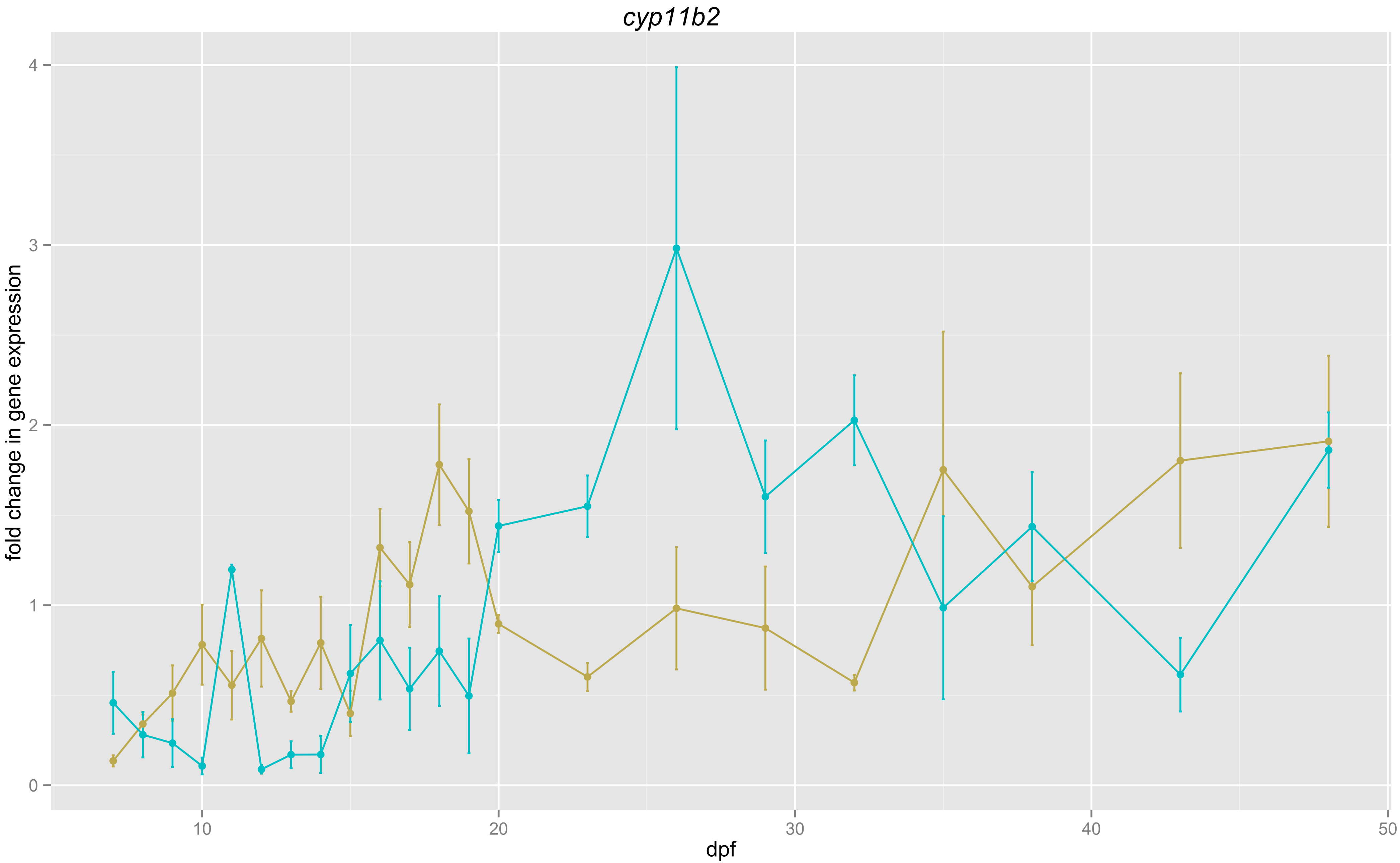

cyp11b2
fold change in gene expression
0
1
2
3
4
10
20
30
40
50
dpf

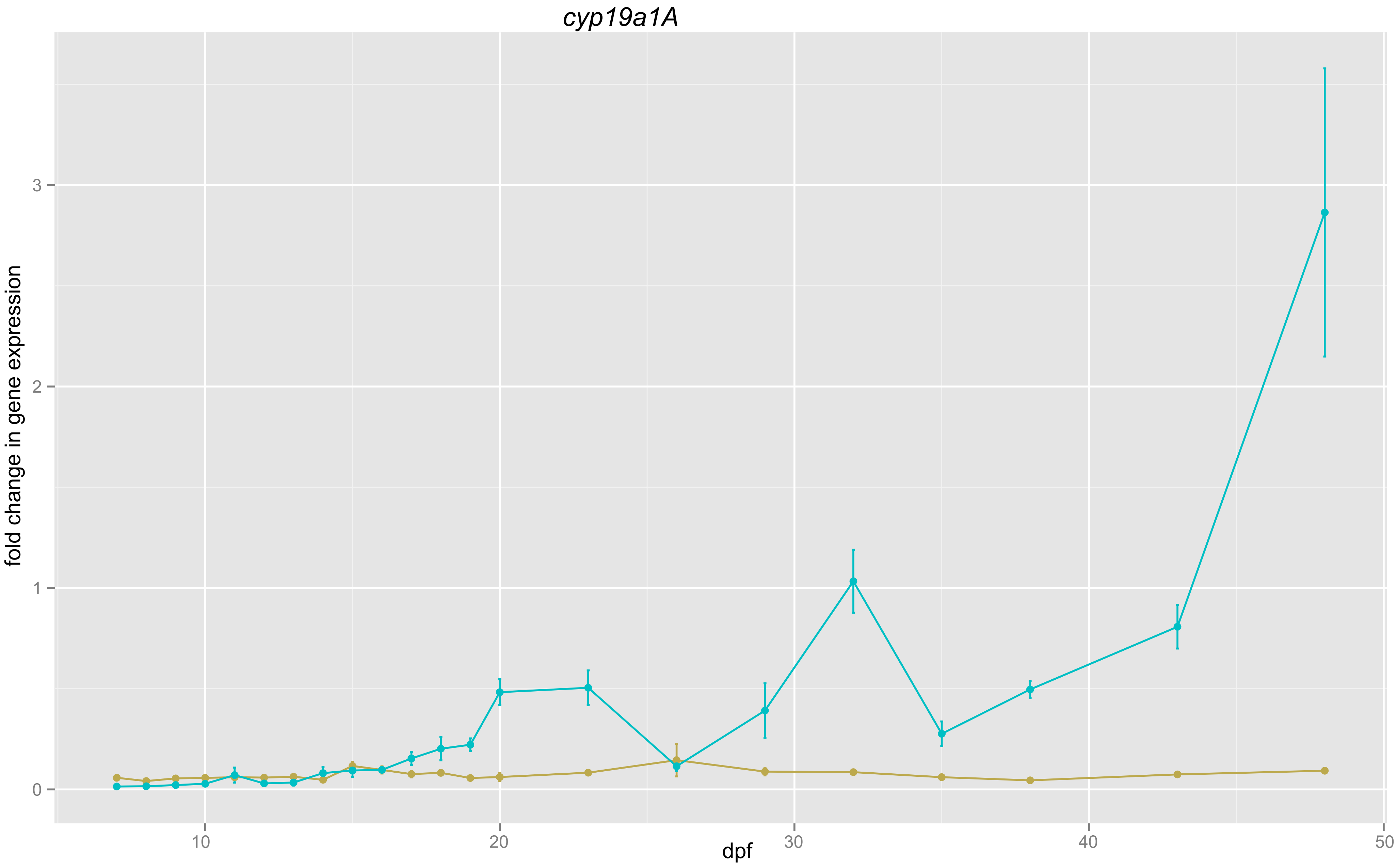
cyp19a1A
fold change in gene expression
dpf
0
1
2
3
10
20
30
40
50

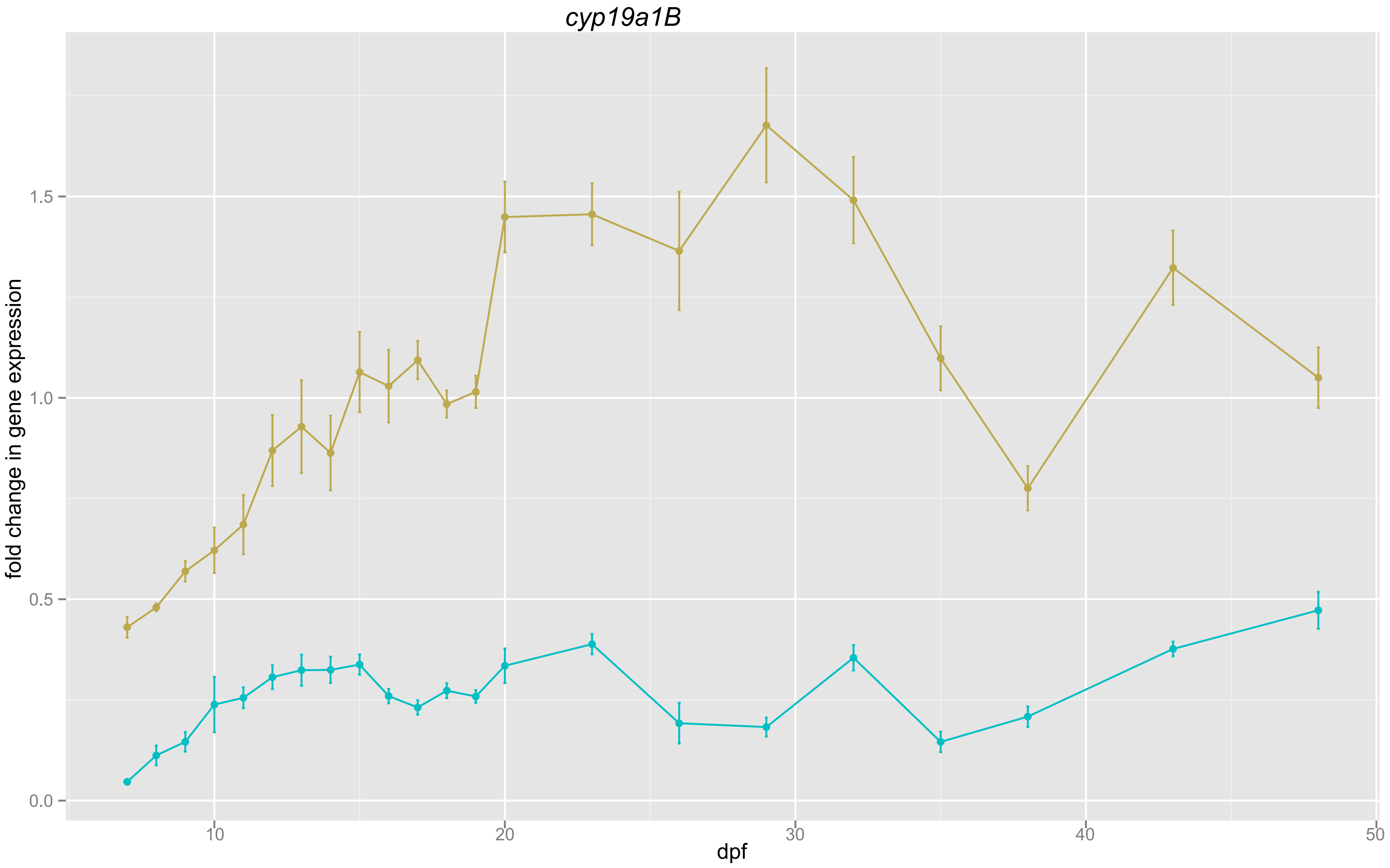

cyp19a1B
fold change in gene expression
1.5
1.0
0.5
0.0
10
20
30
40
50
dpf

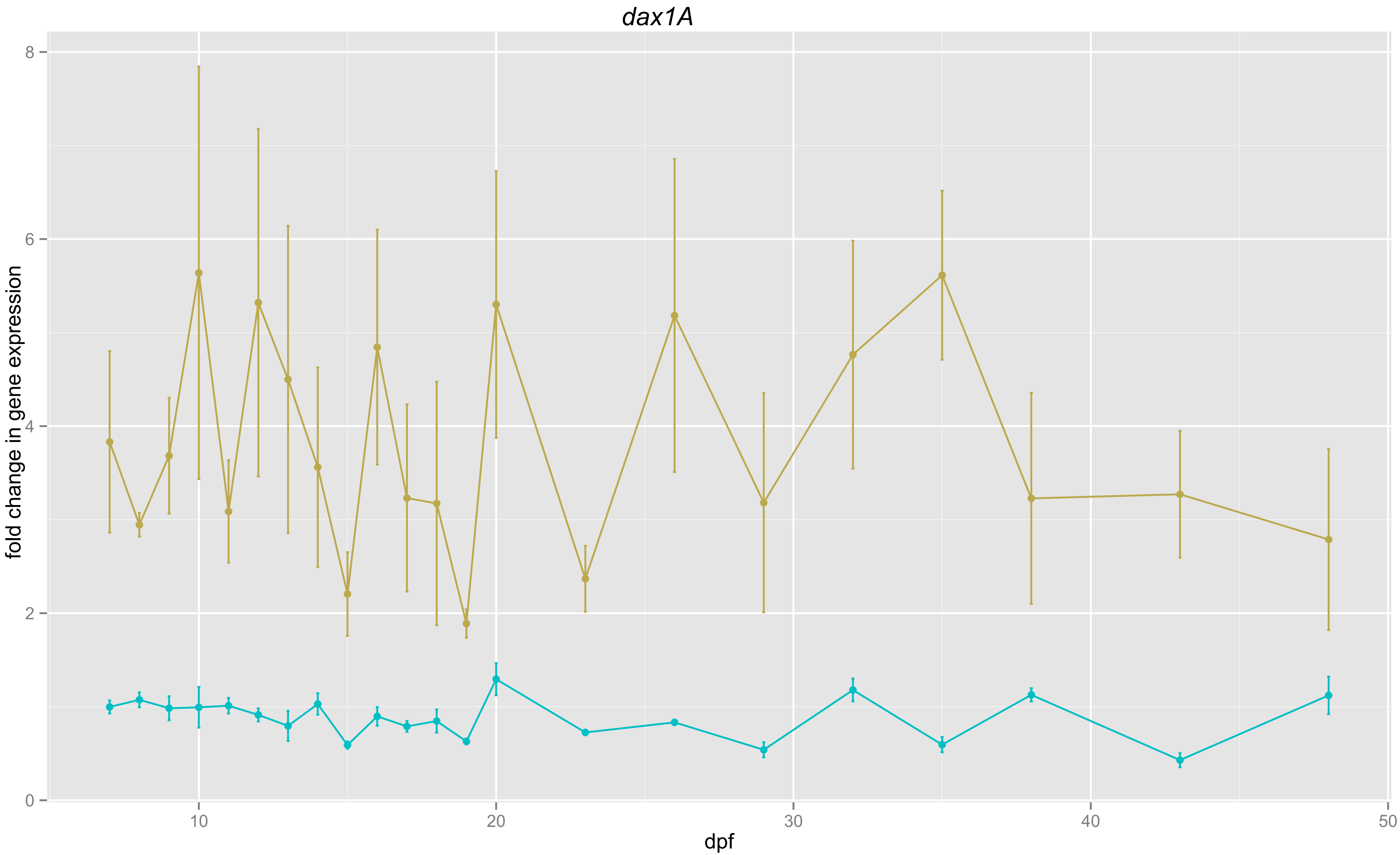

dax1A
fold change in gene expression
0
2
4
6
8
10
20
30
40
50
dpf

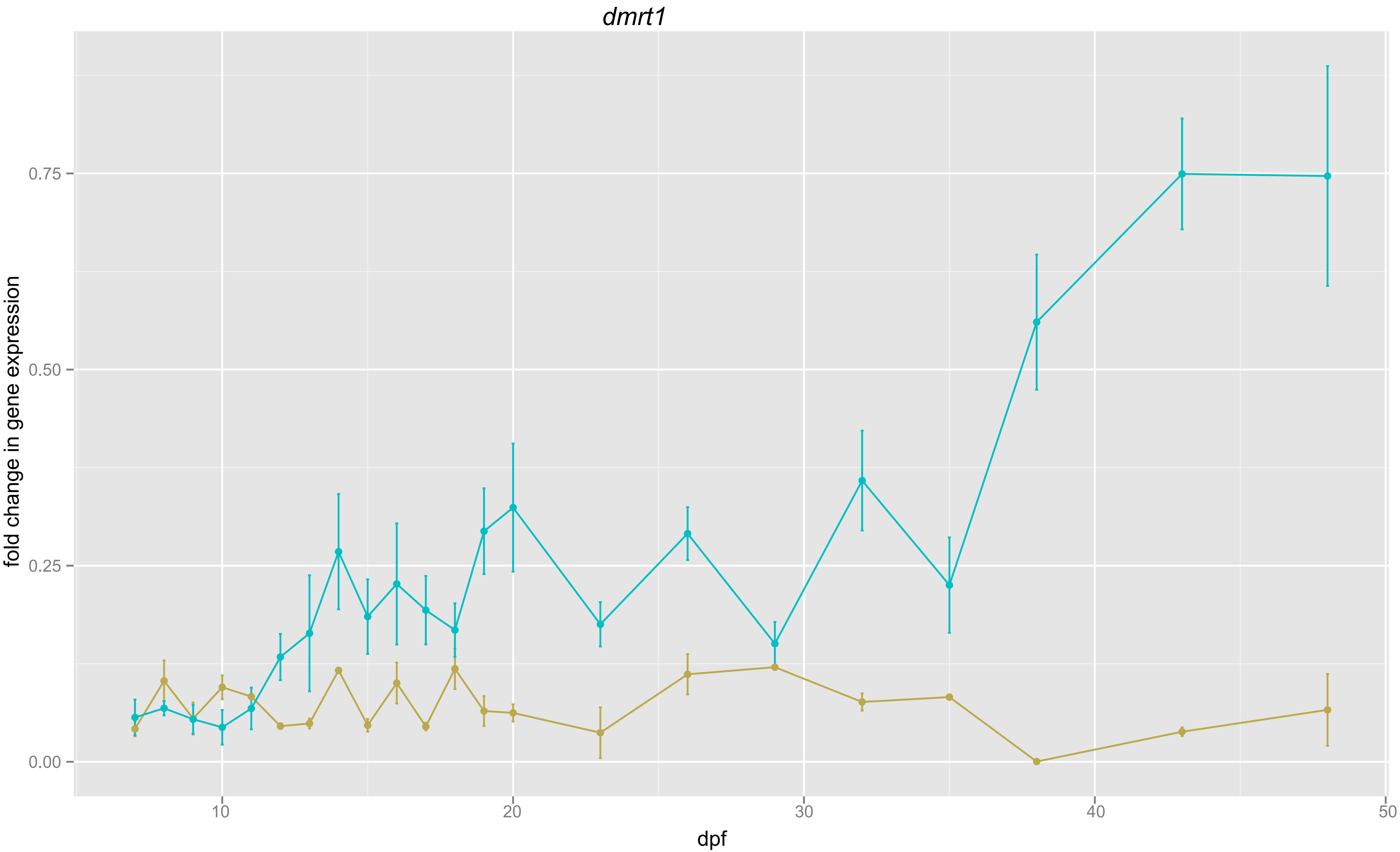
dmrt1
fold change in gene expression
0.00
0.25
0.50
0.75
10
20
30
40
50
dpf

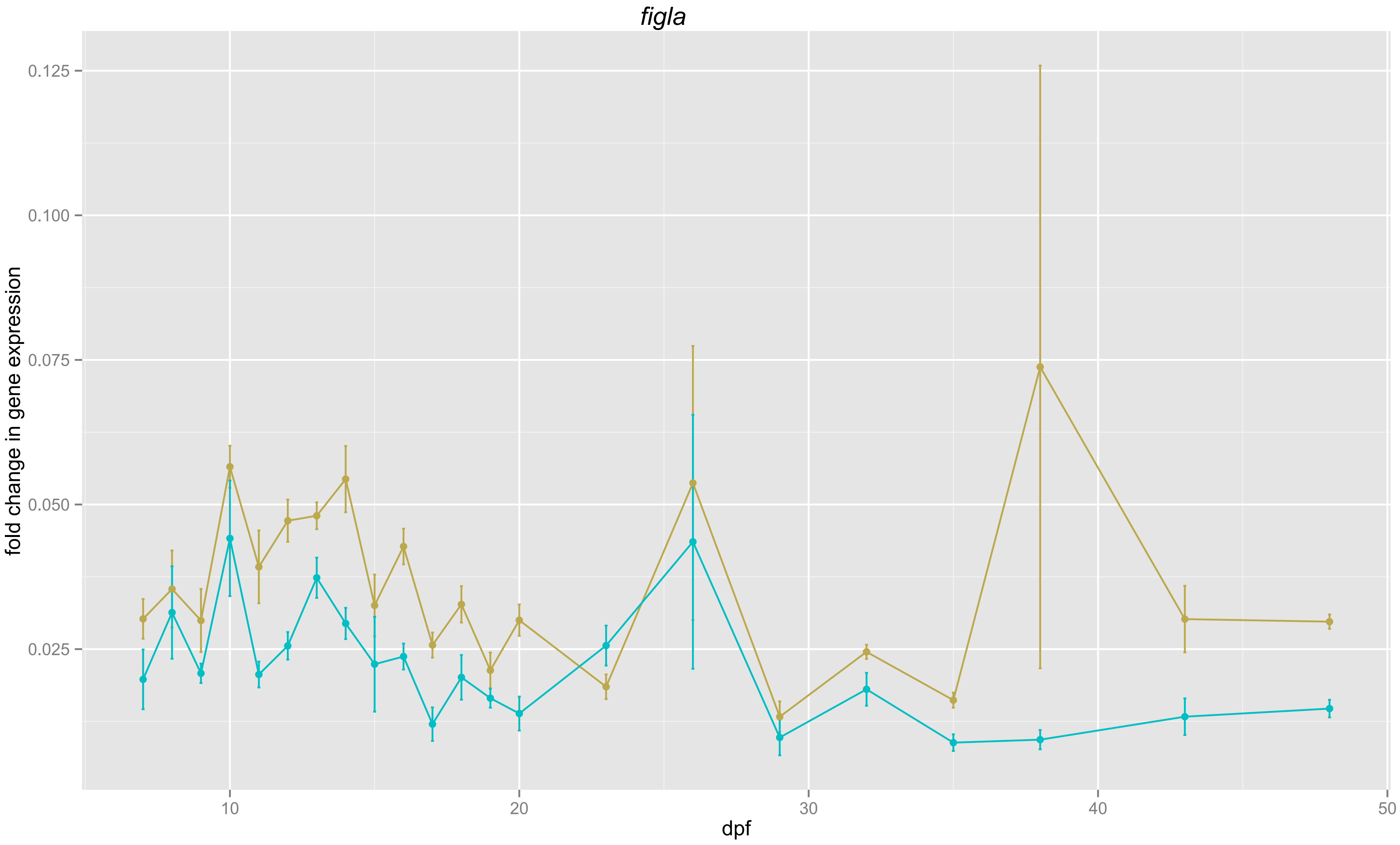
figla
fold change in gene expression
0.125
0.100
0.075
0.050
0.025
10
20
30
40
50
dpf

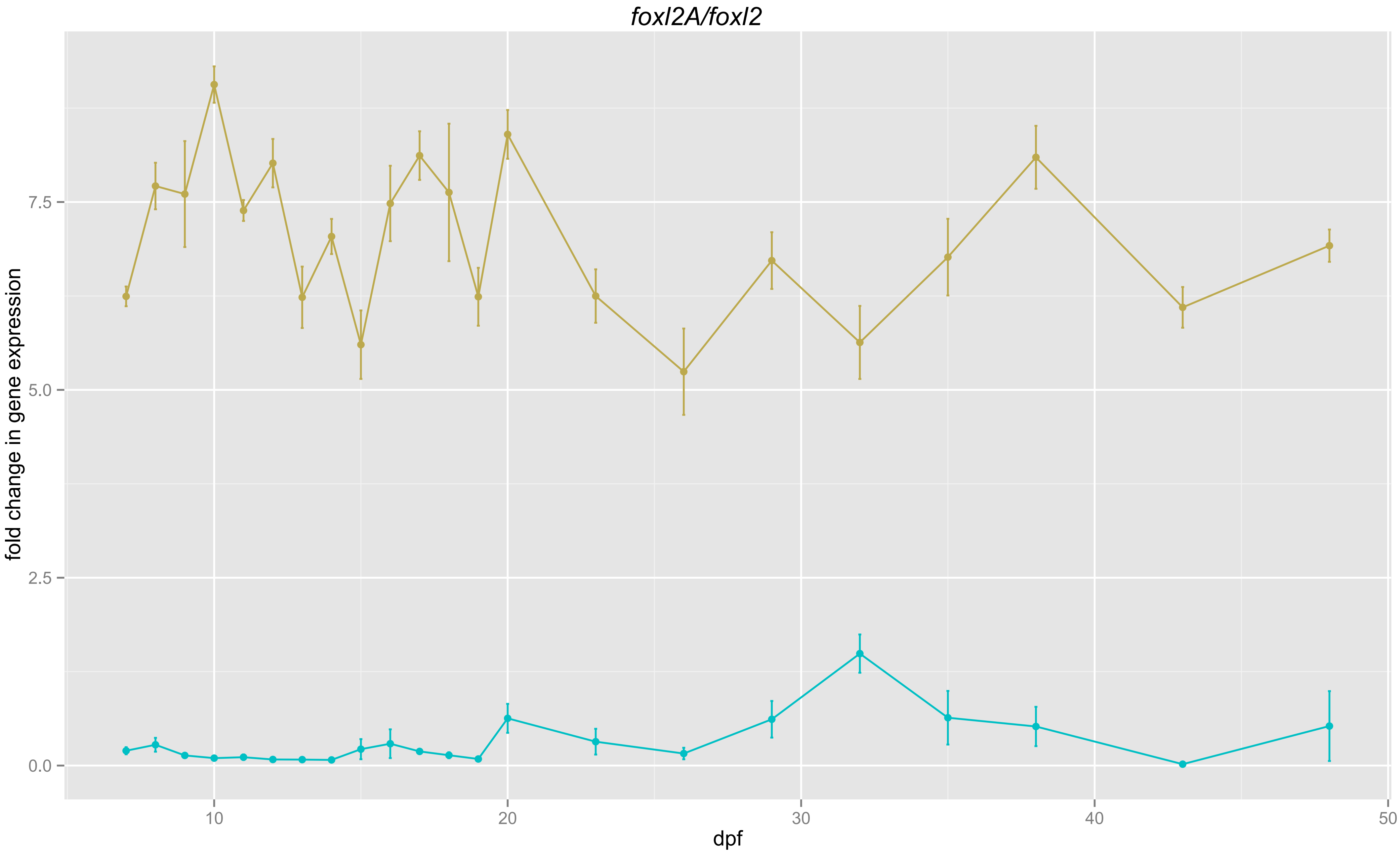

foxl2A/foxl2
fold change in gene expression
7.5
5.0
2.5
0.0
10
20
30
40
50
dpf

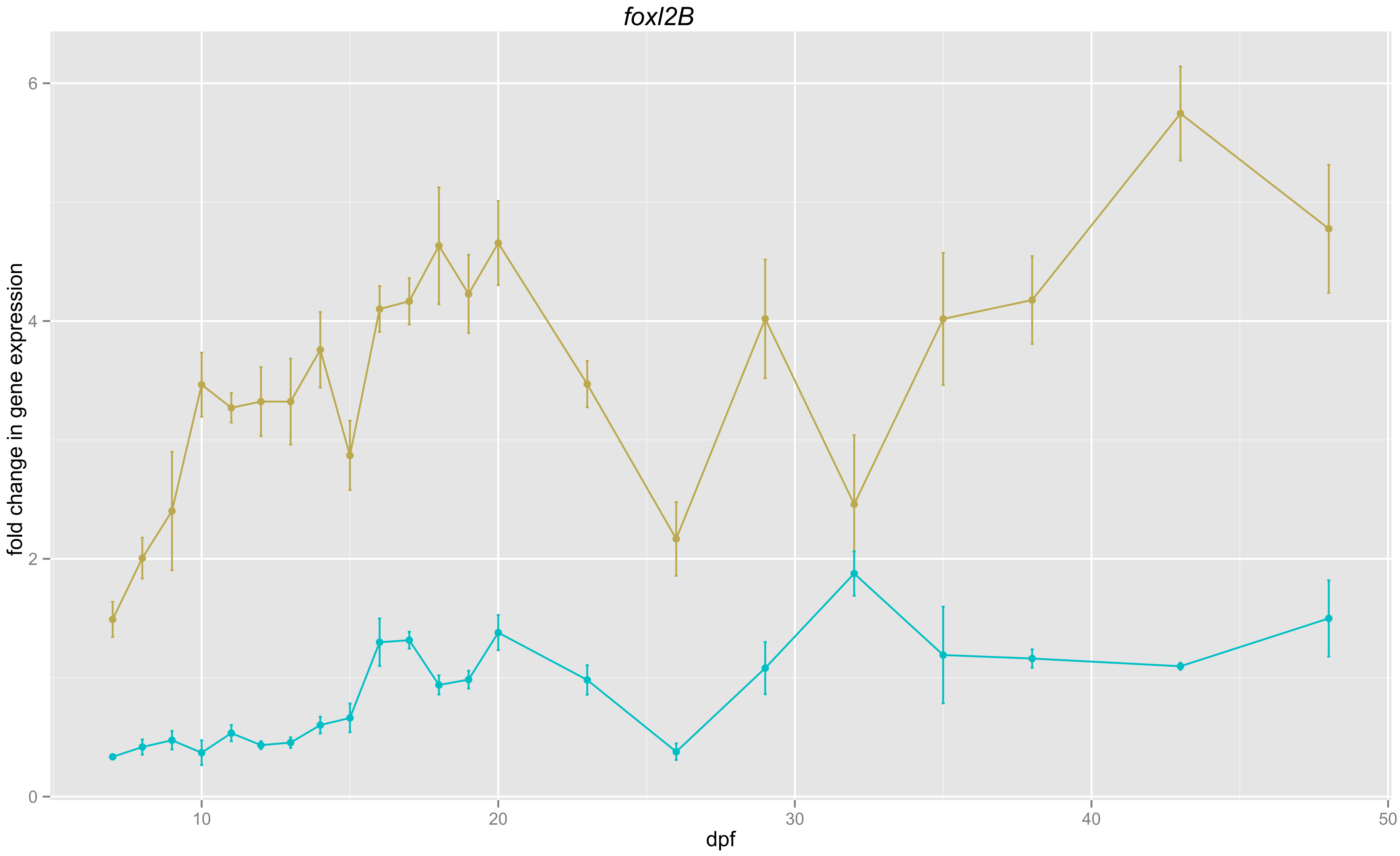

foxl2B
fold change in gene expression
dpf
0
2
4
6
10
20
30
40
50

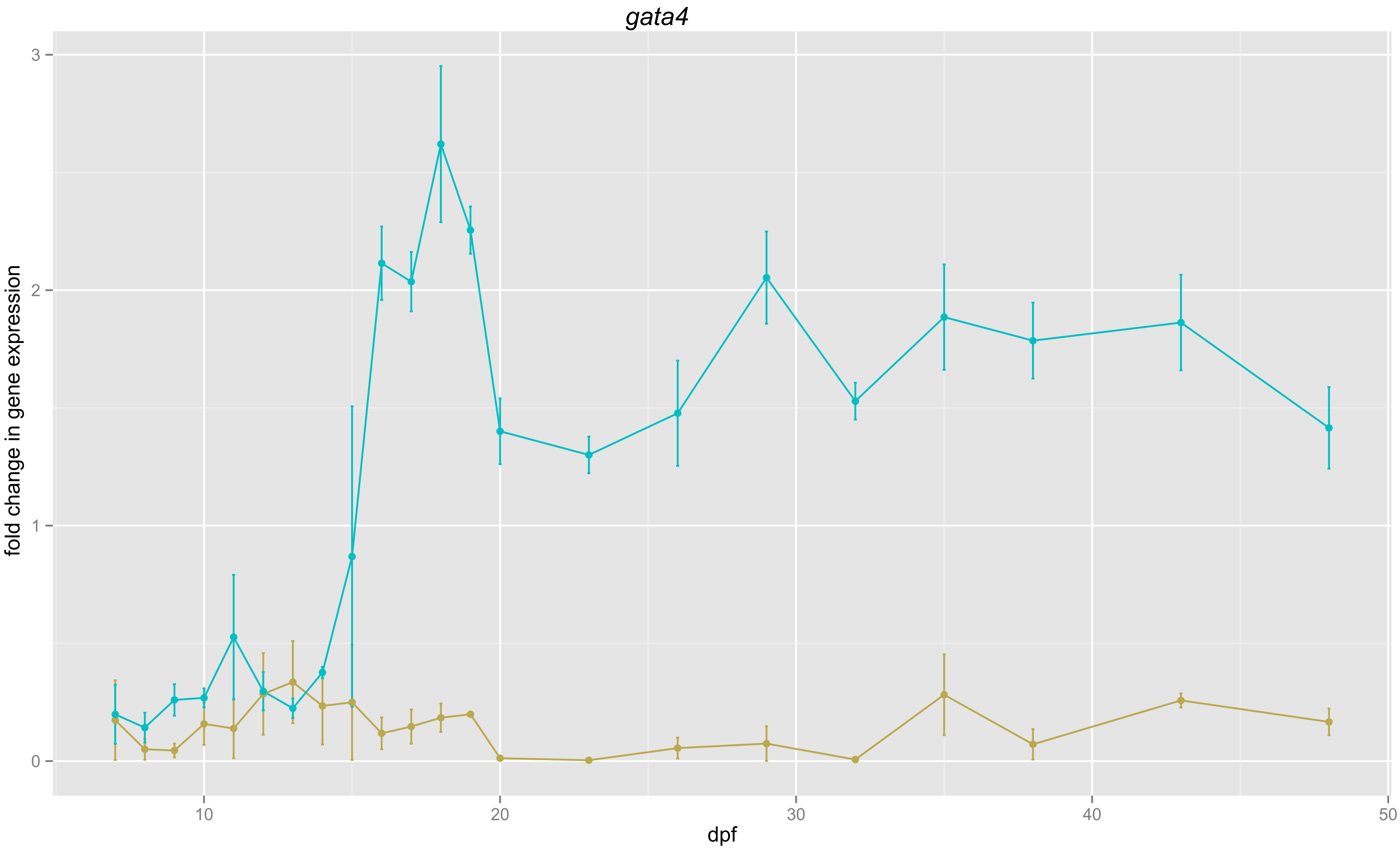

gata4
fold change in gene expression
0
1
2
3
10
20
30
40
50
dpf

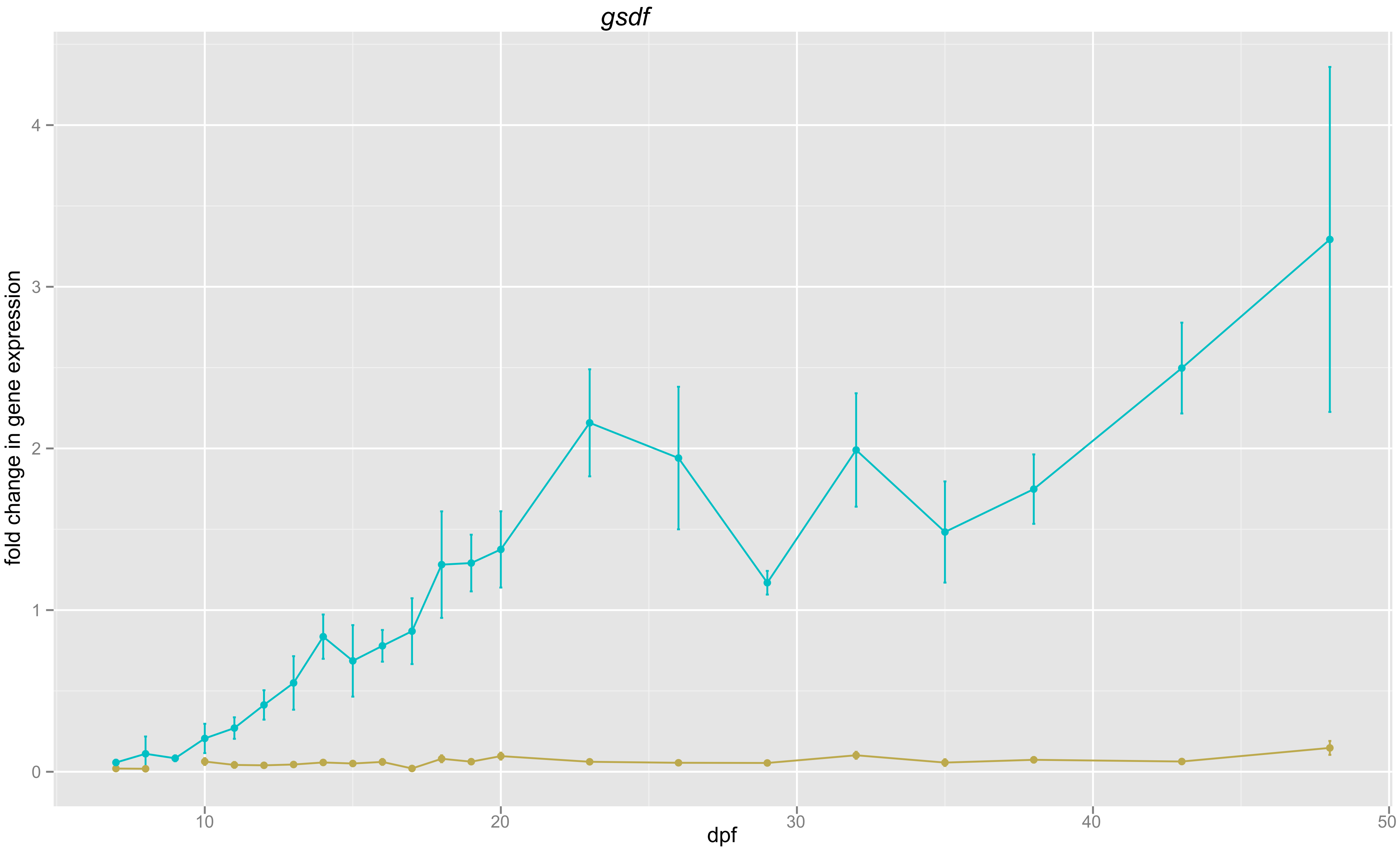

gsdf
fold change in gene expression
0
1
2
3
4
10
20
30
40
50
dpf

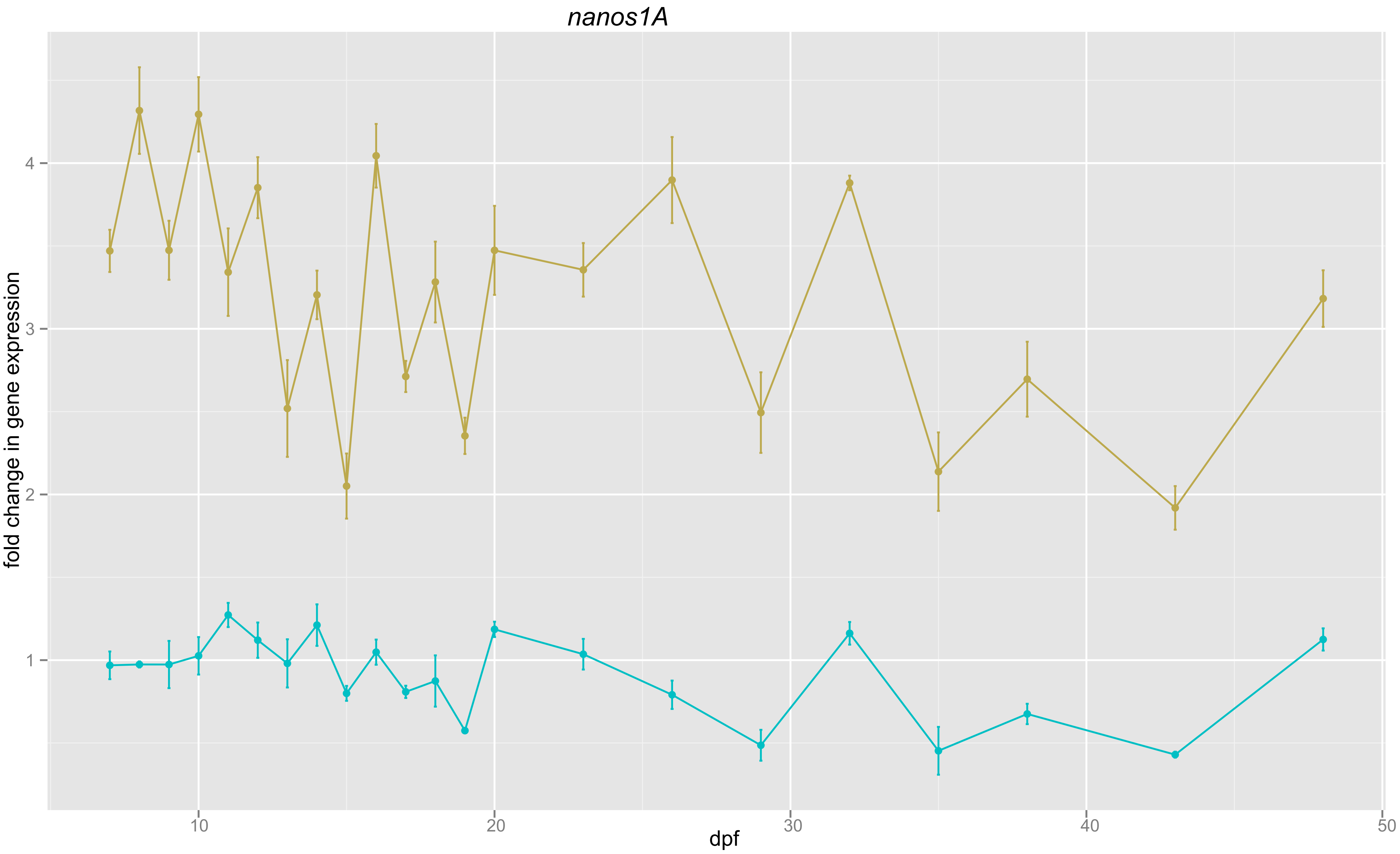

nanos1A
fold change in gene expression
1
2
3
4
dpf
10
20
30
40
50

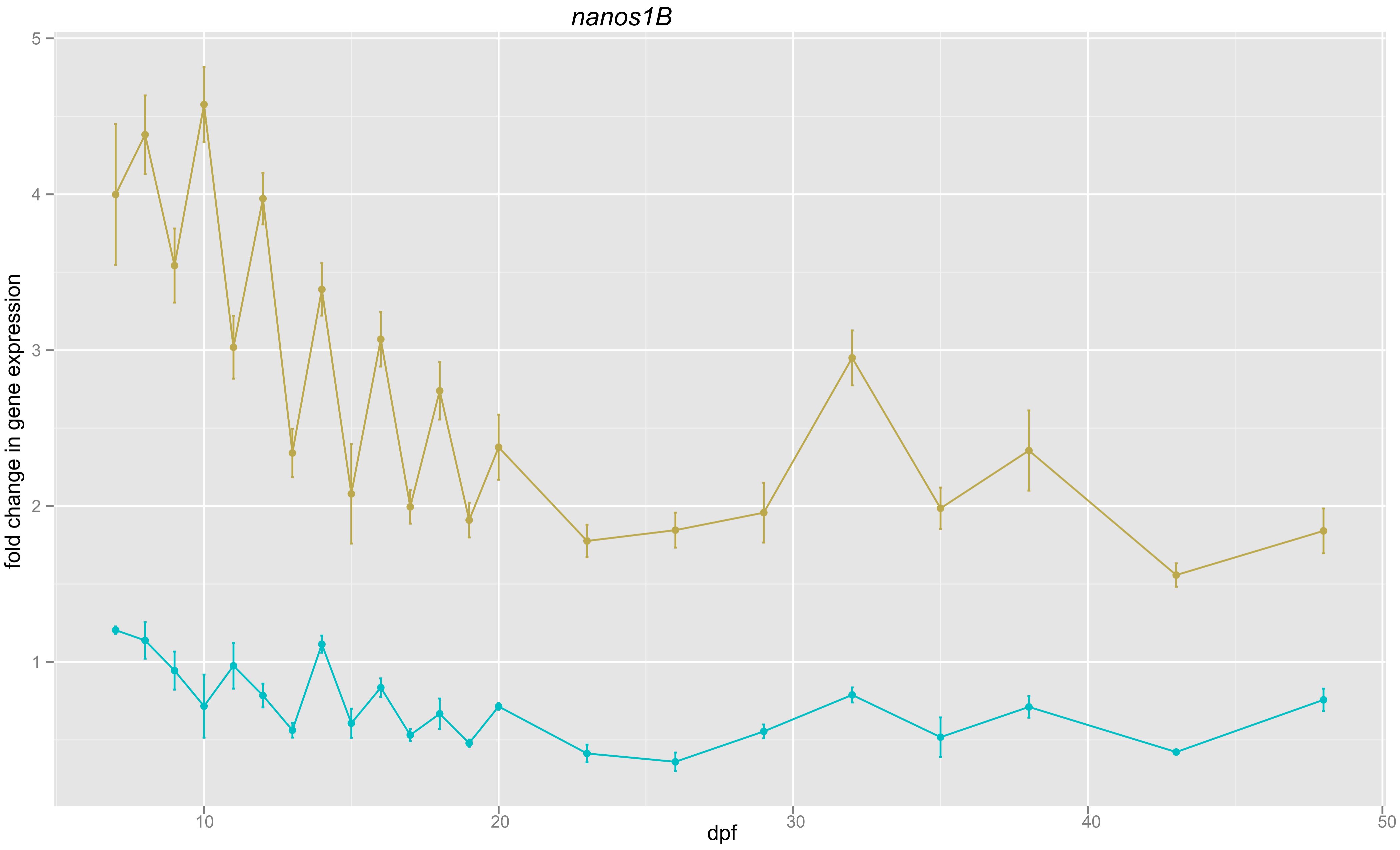
nanos1B
fold change in gene expression
dpf
1
2
3
4
5
10
20
30
40
50

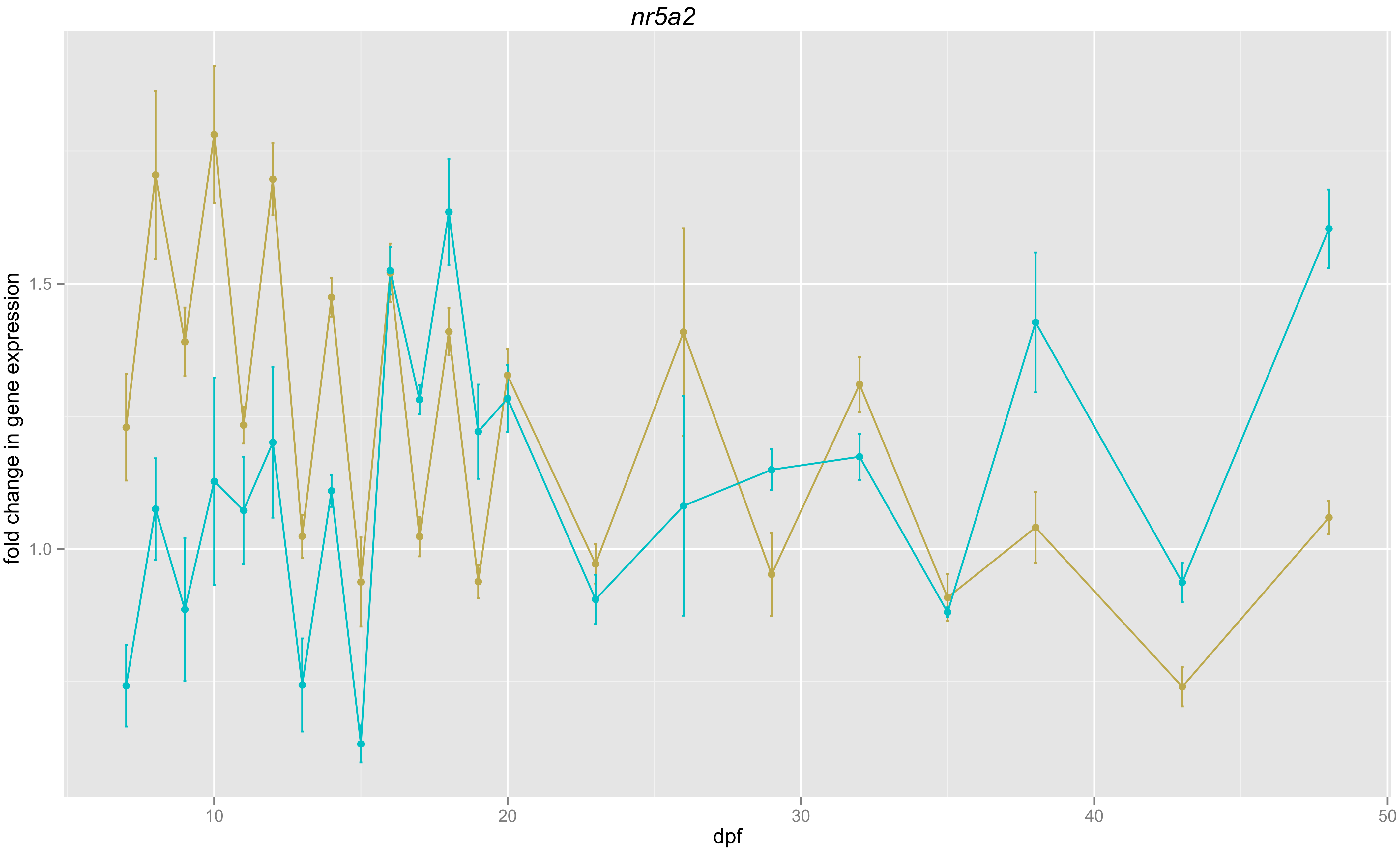
nr5a2
fold change in gene expression
1.5
1.0
10
20
30
40
50
dpf

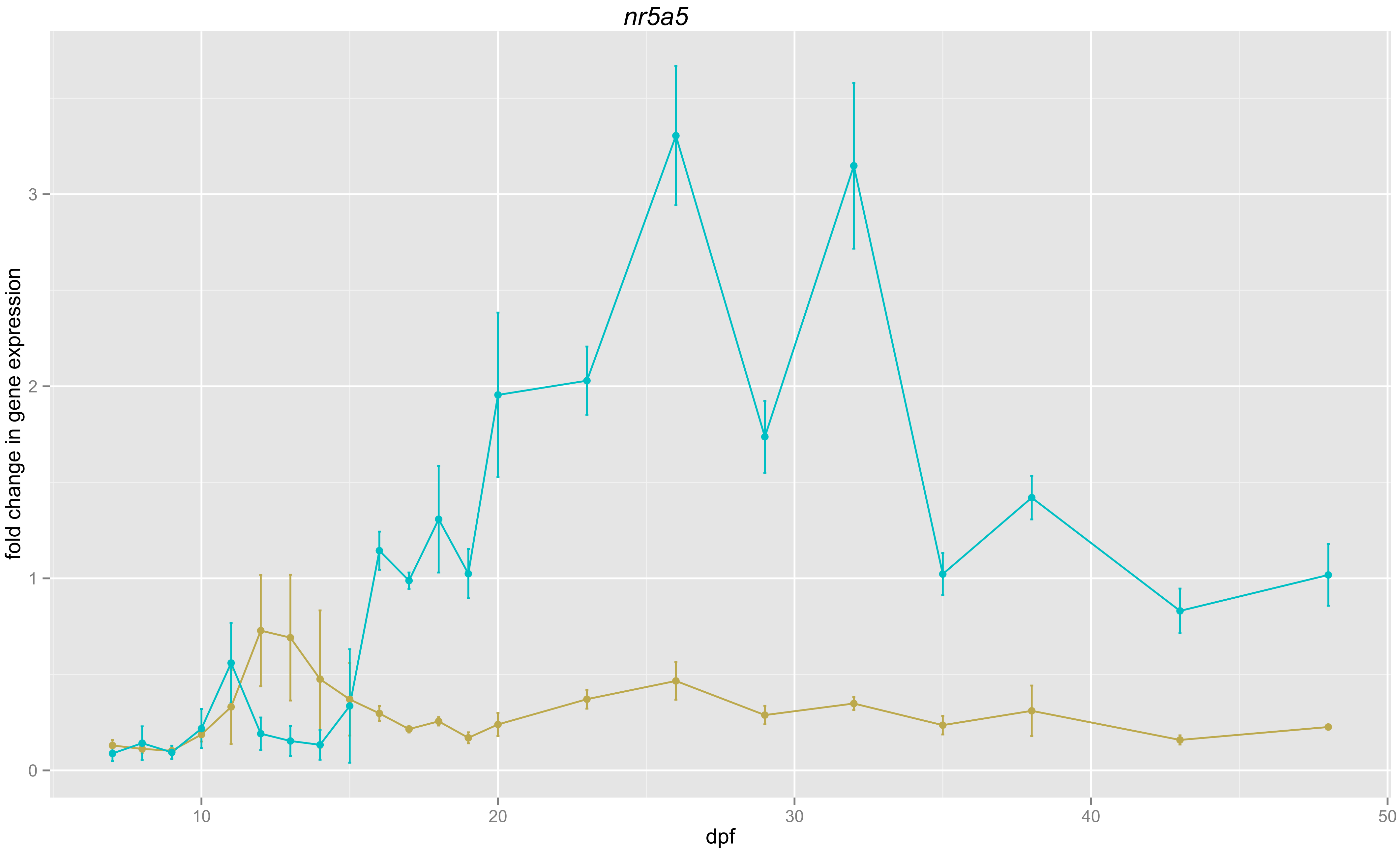

nr5a5
fold change in gene expression
0
1
2
3
10
20
30
40
50
dpf

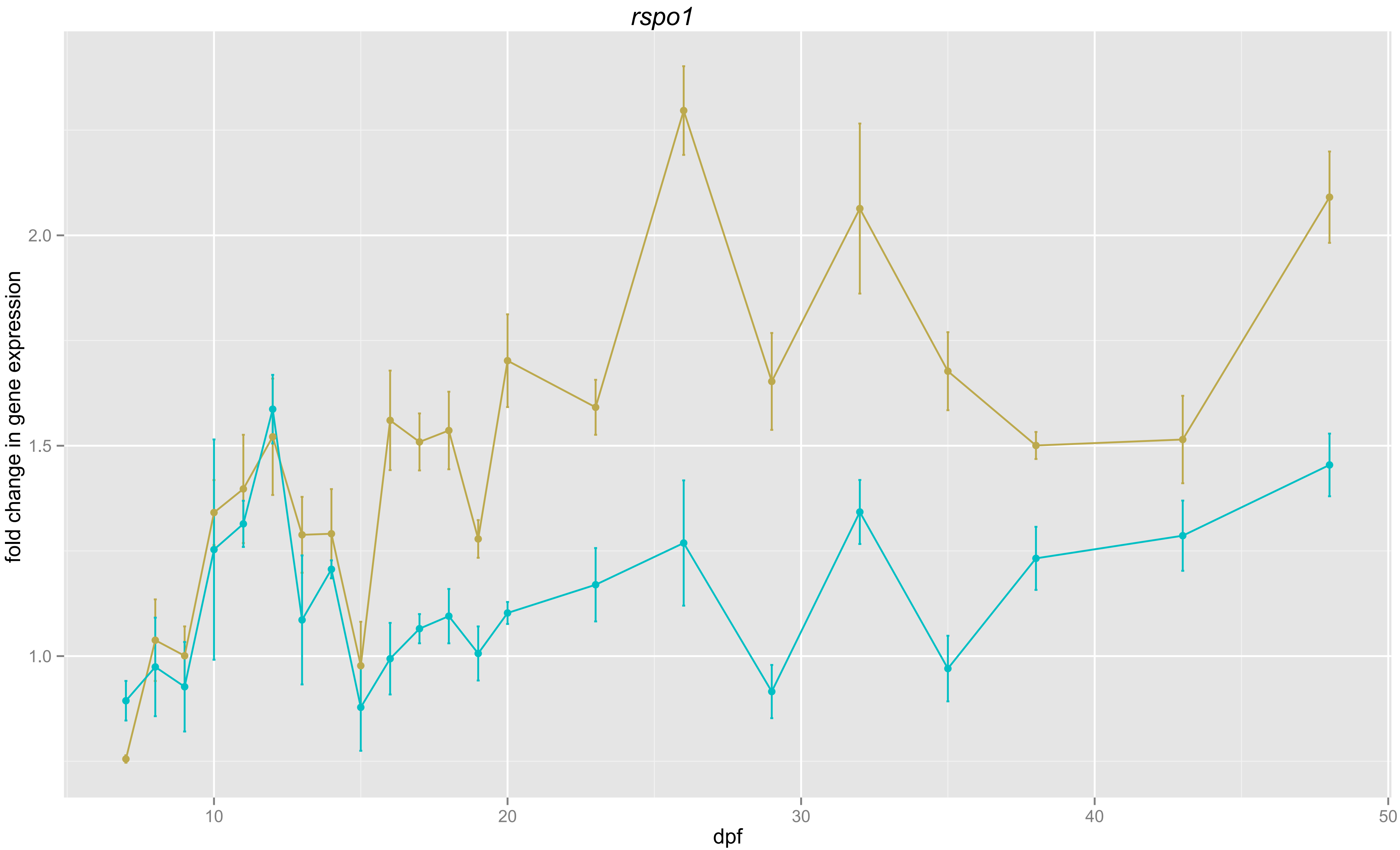

rspo1
fold change in gene expression
2.0
1.5
1.0
10
20
30
40
50
dpf

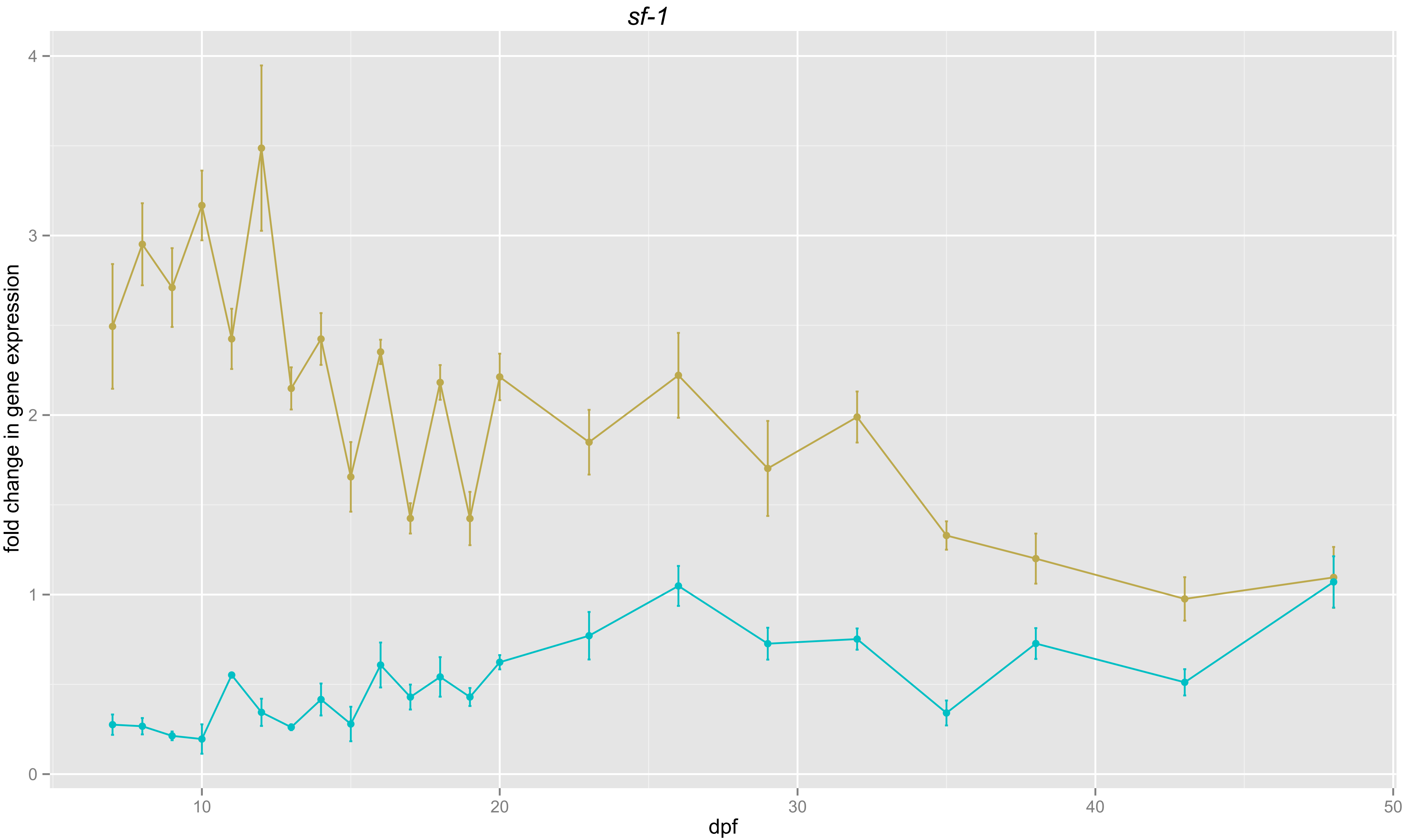

sf-1
fold change in gene expression
dpf
0
1
2
3
4
10
20
30
40
50

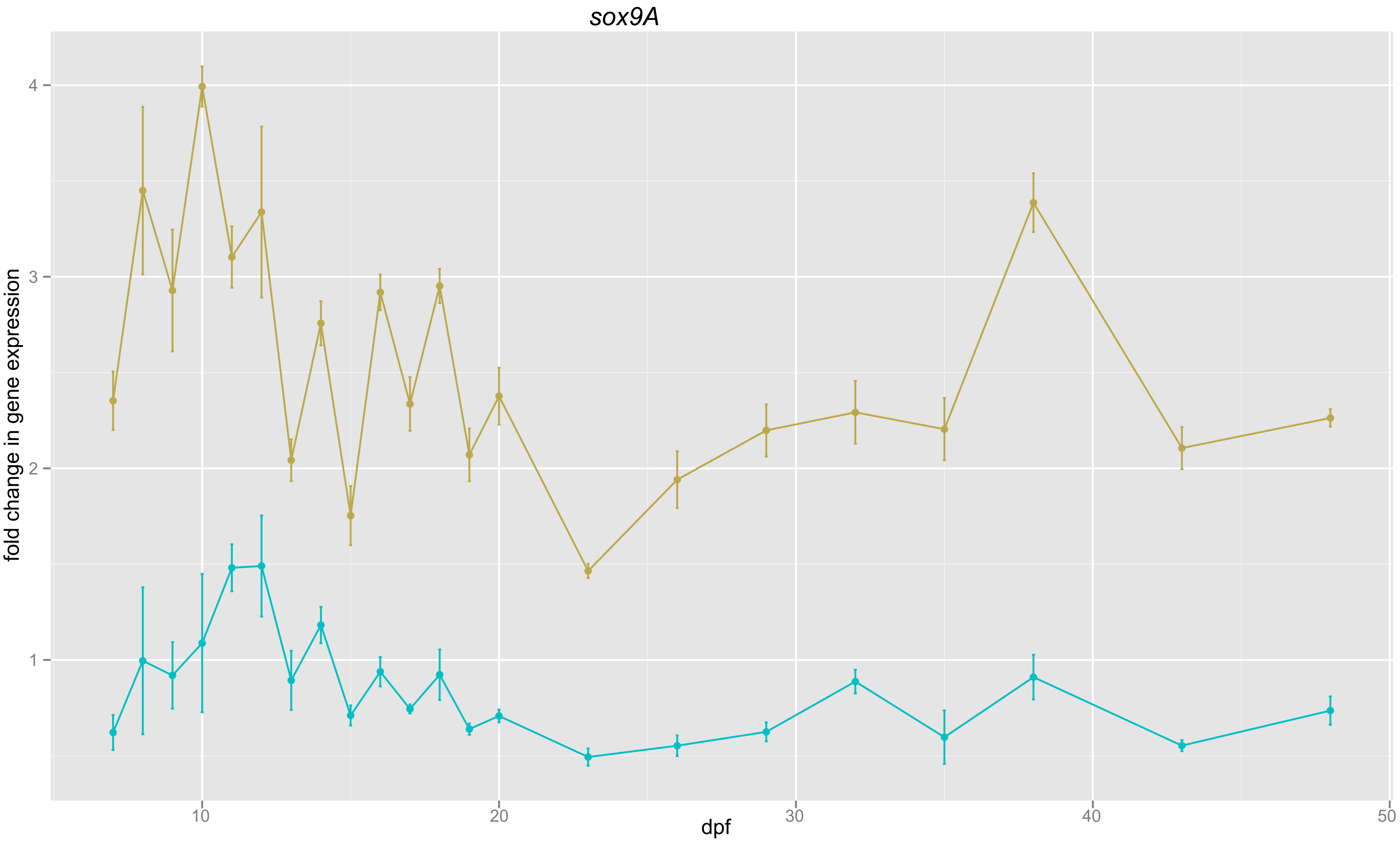

sox9A
fold change in gene expression
1
2
3
4
10
20
30
40
50
dpf

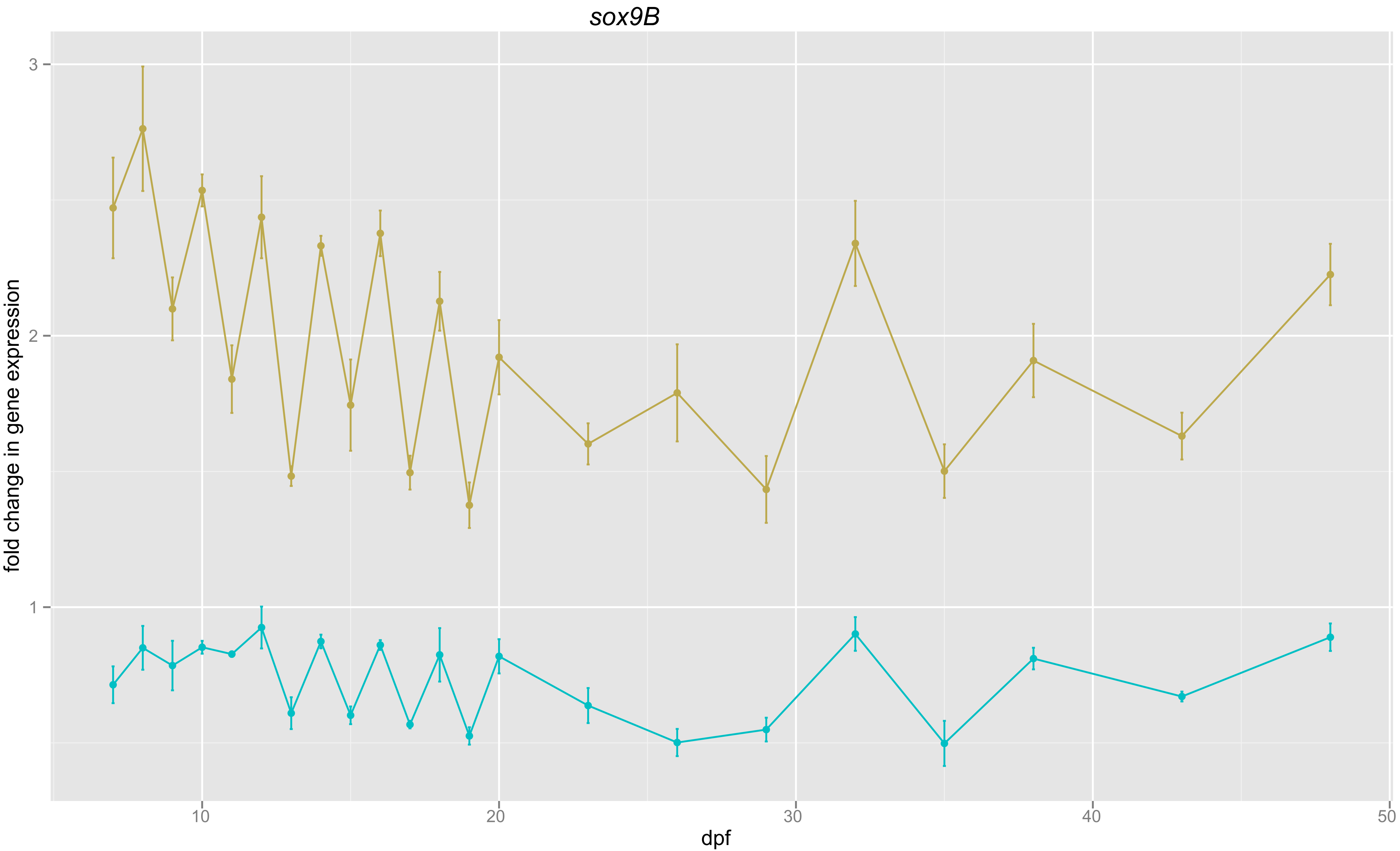

sox9B
fold change in gene expression
3
2
1
10
20
30
40
50
dpf

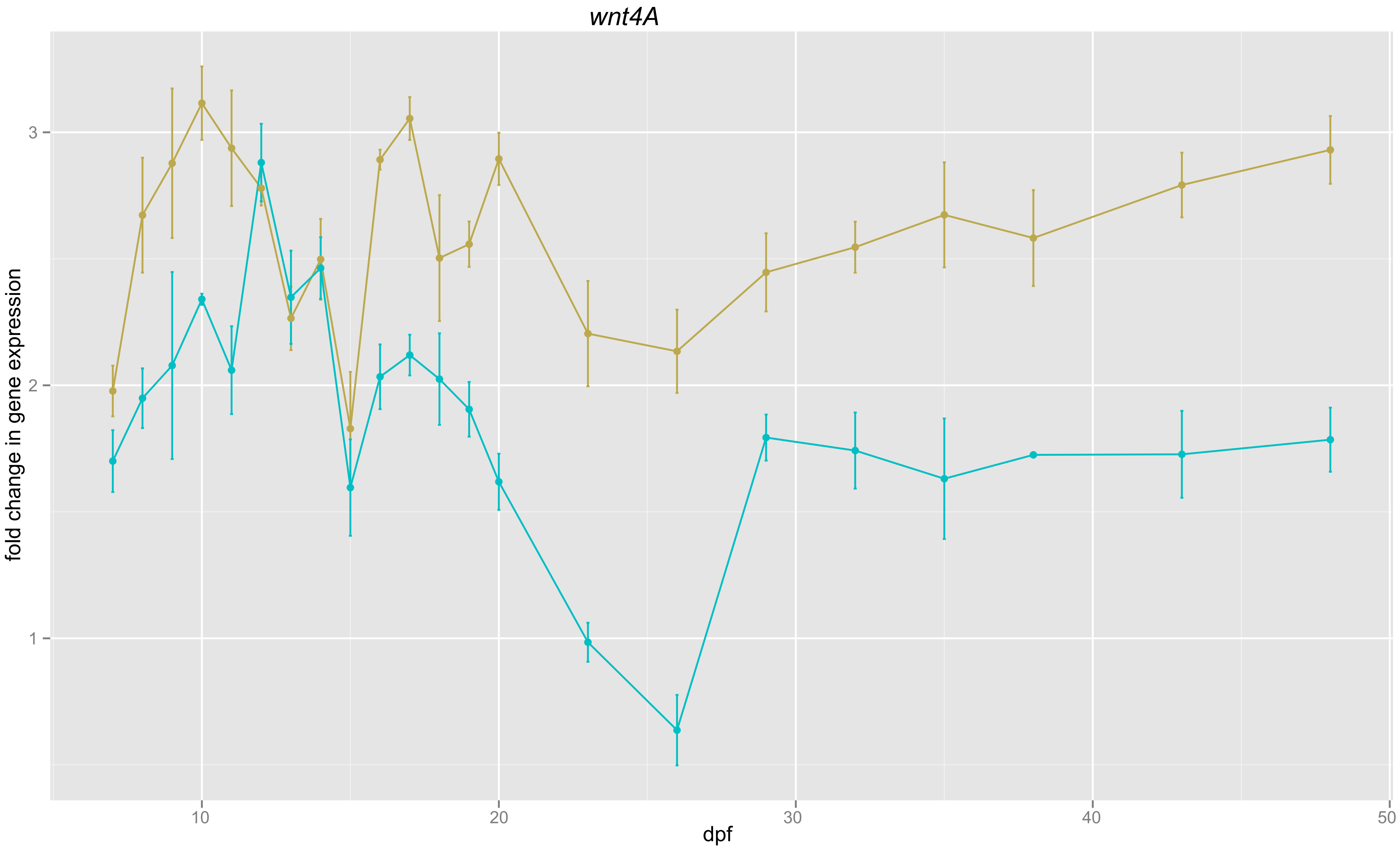

wnt4A
fold change in gene expression
3
2
1
10
20
30
40
50
dpf

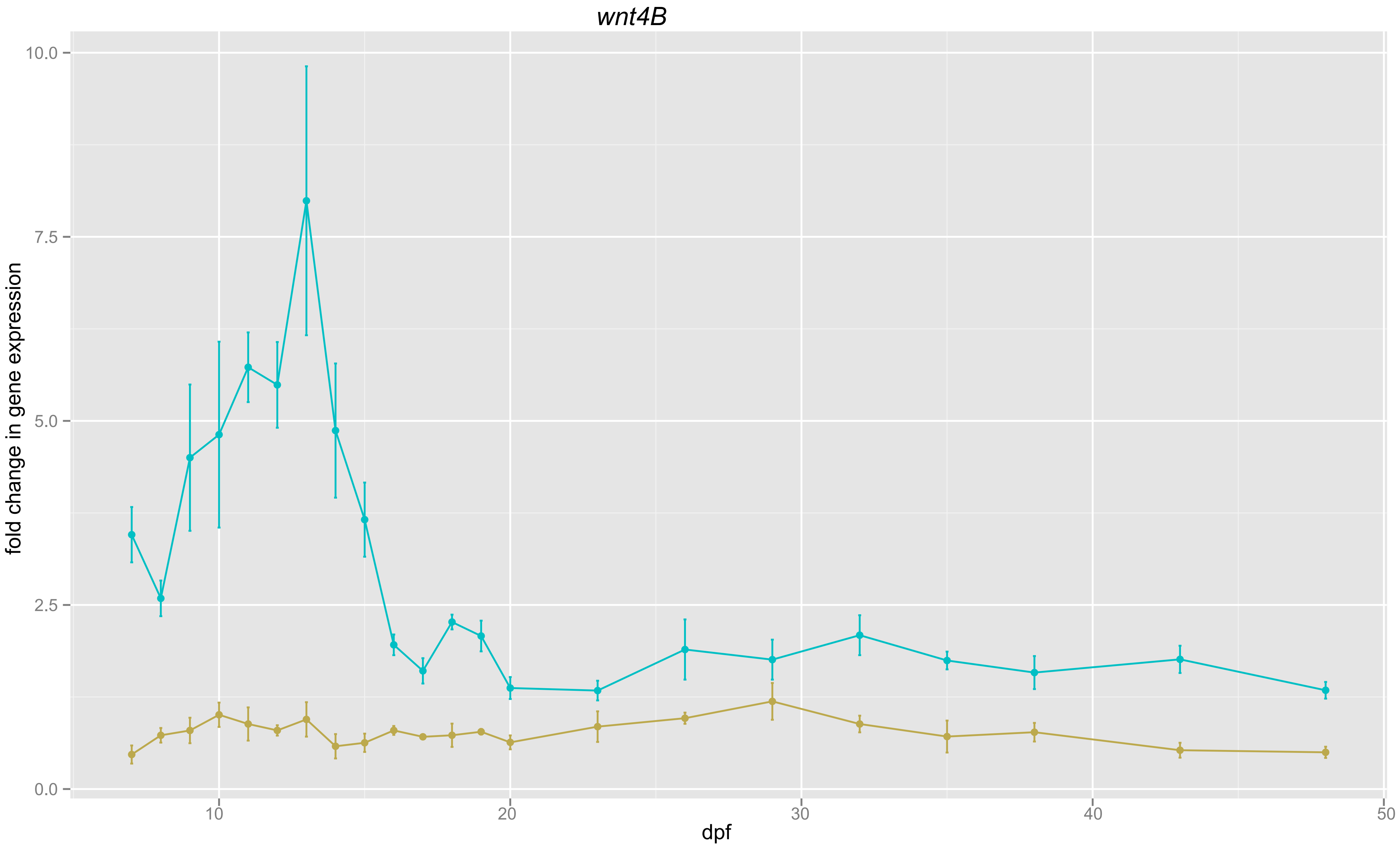

wnt4B
fold change in gene expression
10.0
7.5
5.0
2.5
0.0
10
20
30
40
50
dpf

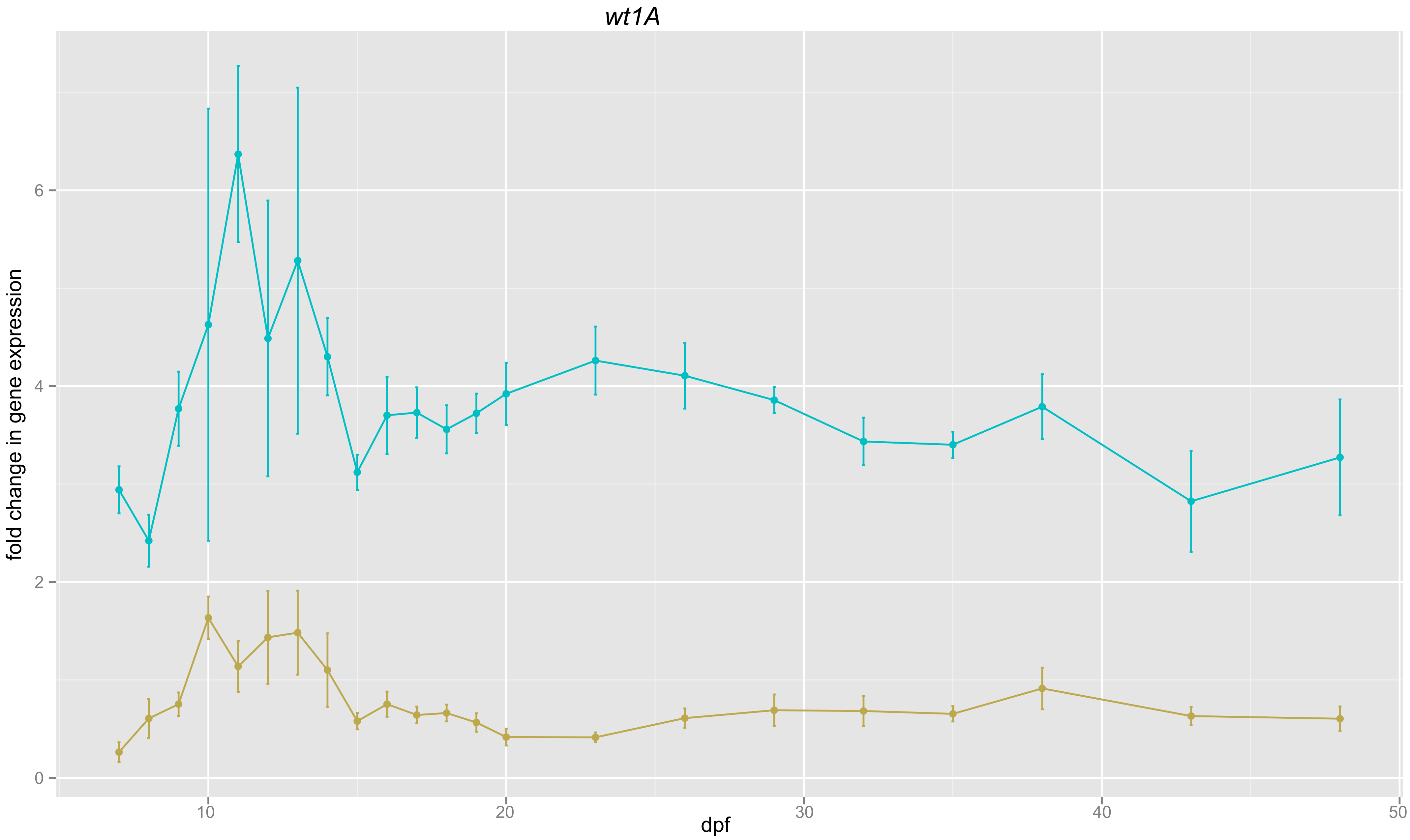

wt1A
fold change in gene expression
dpf
0
2
4
6
10
20
30
40
50

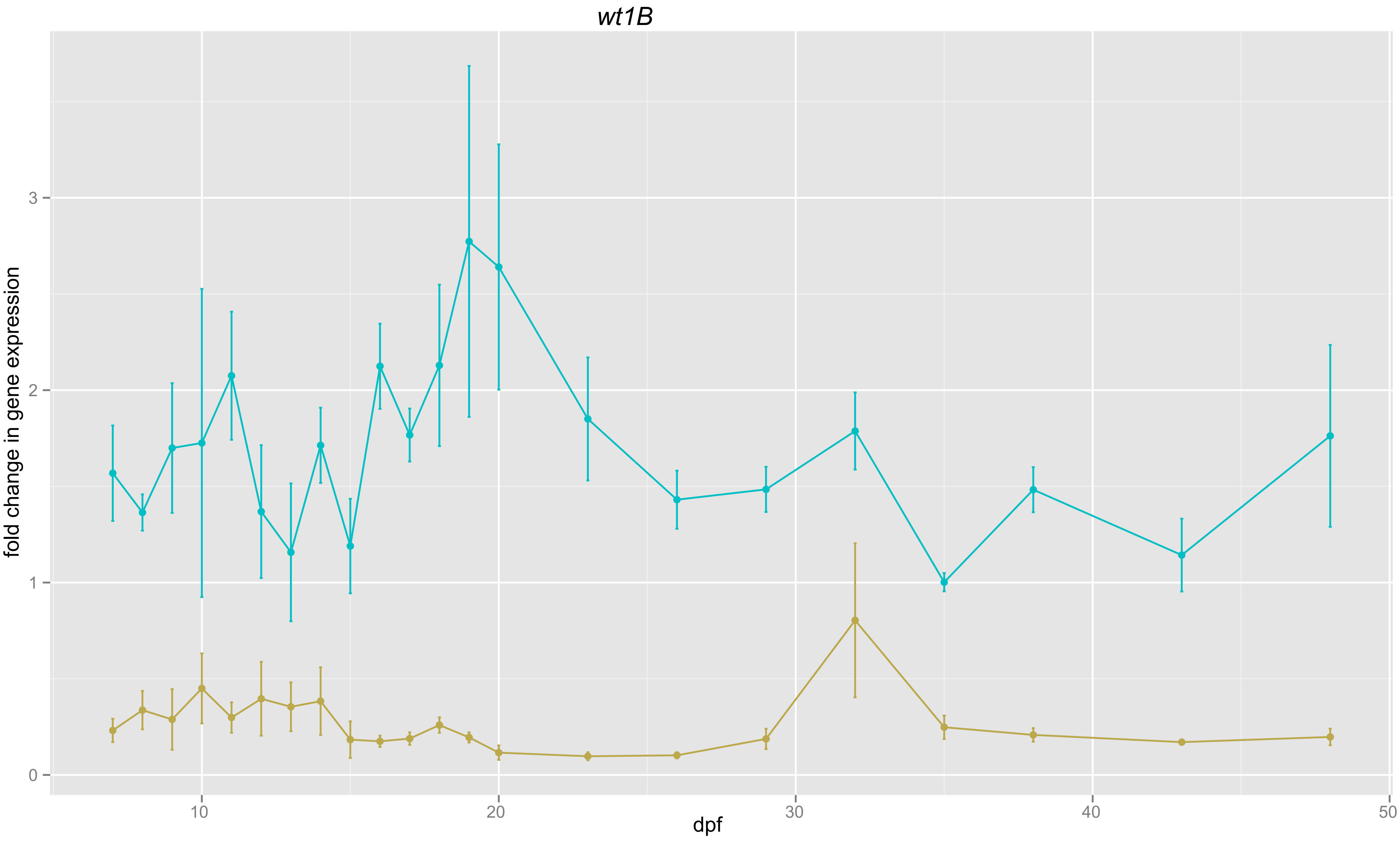

wt1B
fold change in gene expression
0
1
2
3
10
20
30
40
50
dpf
