# Supplementary material for: Genetics and timing of sex determination in the East African cichlid fish Astatotilapia burtoni
Source: BMC Genet. 2014 Dec 14;15:140. doi: 10.1186/s12863-014-0140-5 (PMC4278230; doi:10.1186/s12863-014-0140-5)
Supplement: Additional file 2: — Expression data of all candidate genes during development of A. burtoni . Gene expression as fold change (Livak) ± SE in heads and trunks relative to the first sampling point at 7 dpf. For details on sample size see Additional file 3. [file 12863_2014_140_MOESM2_ESM.pdf]

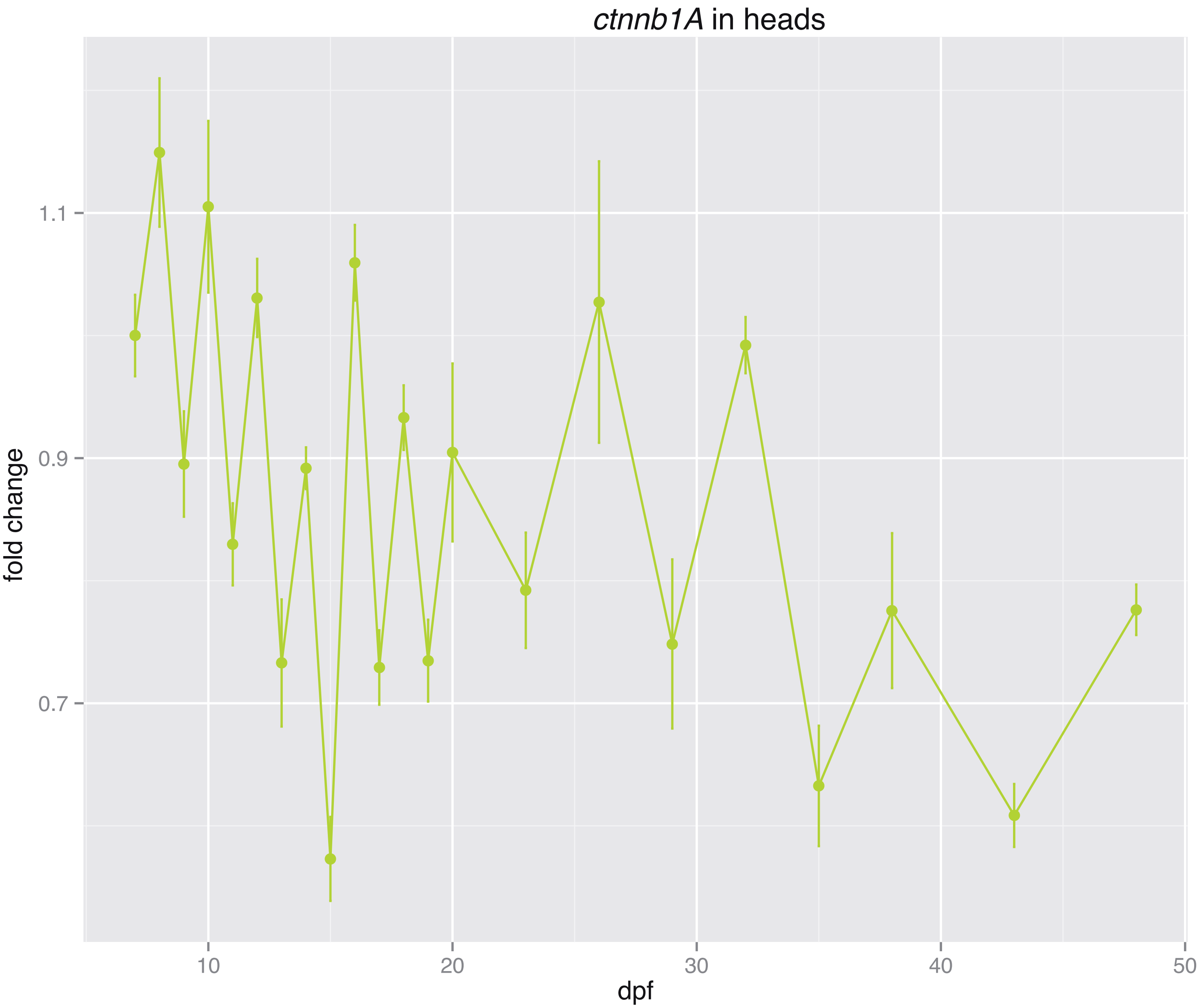

ctnnb1A in heads
fold change
1.1
0.9
0.7
dpf
10
20
30
40
50

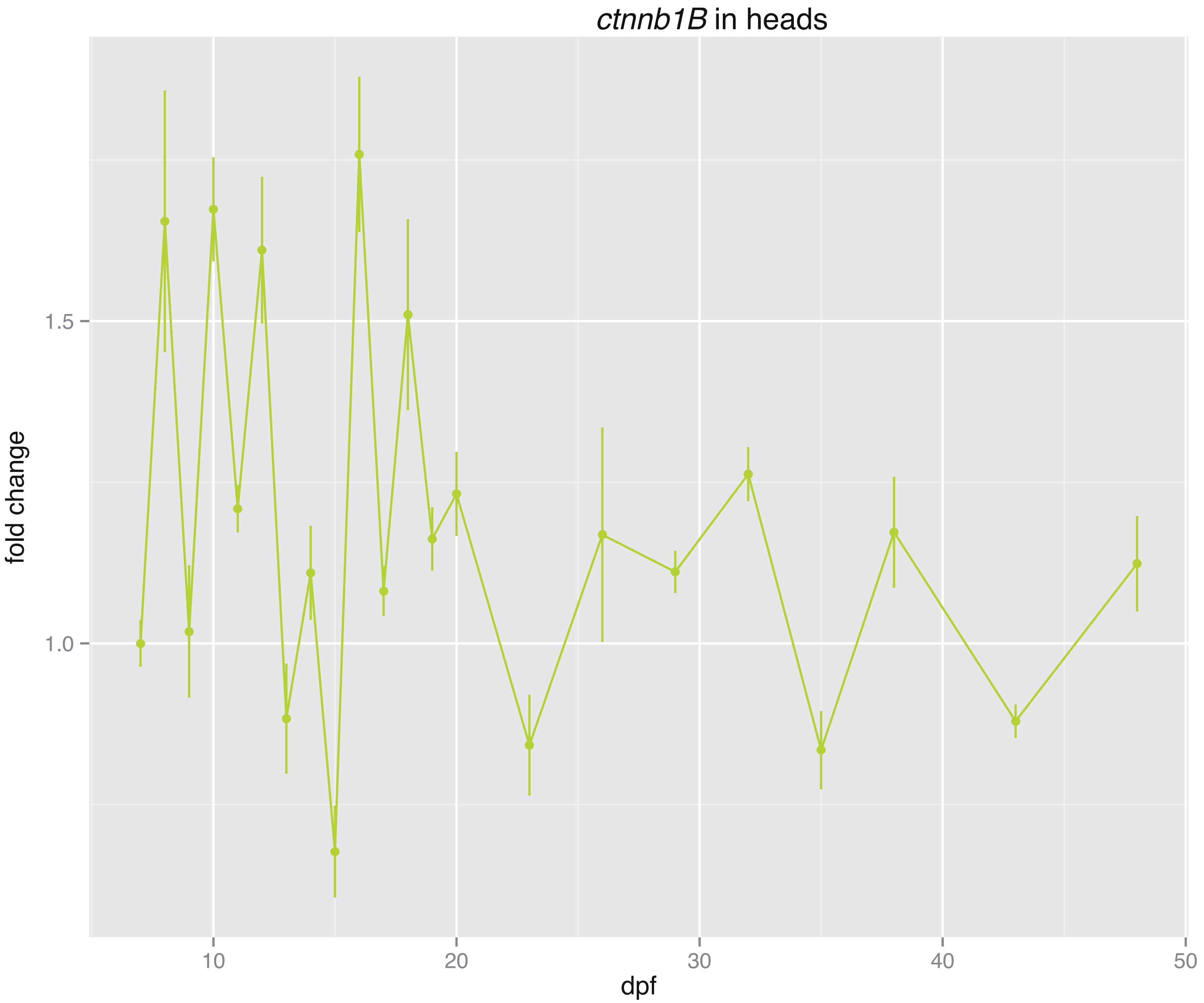
ctnnb1B in heads
fold change
1.5
1.0
10
20
30
40
50
dpf

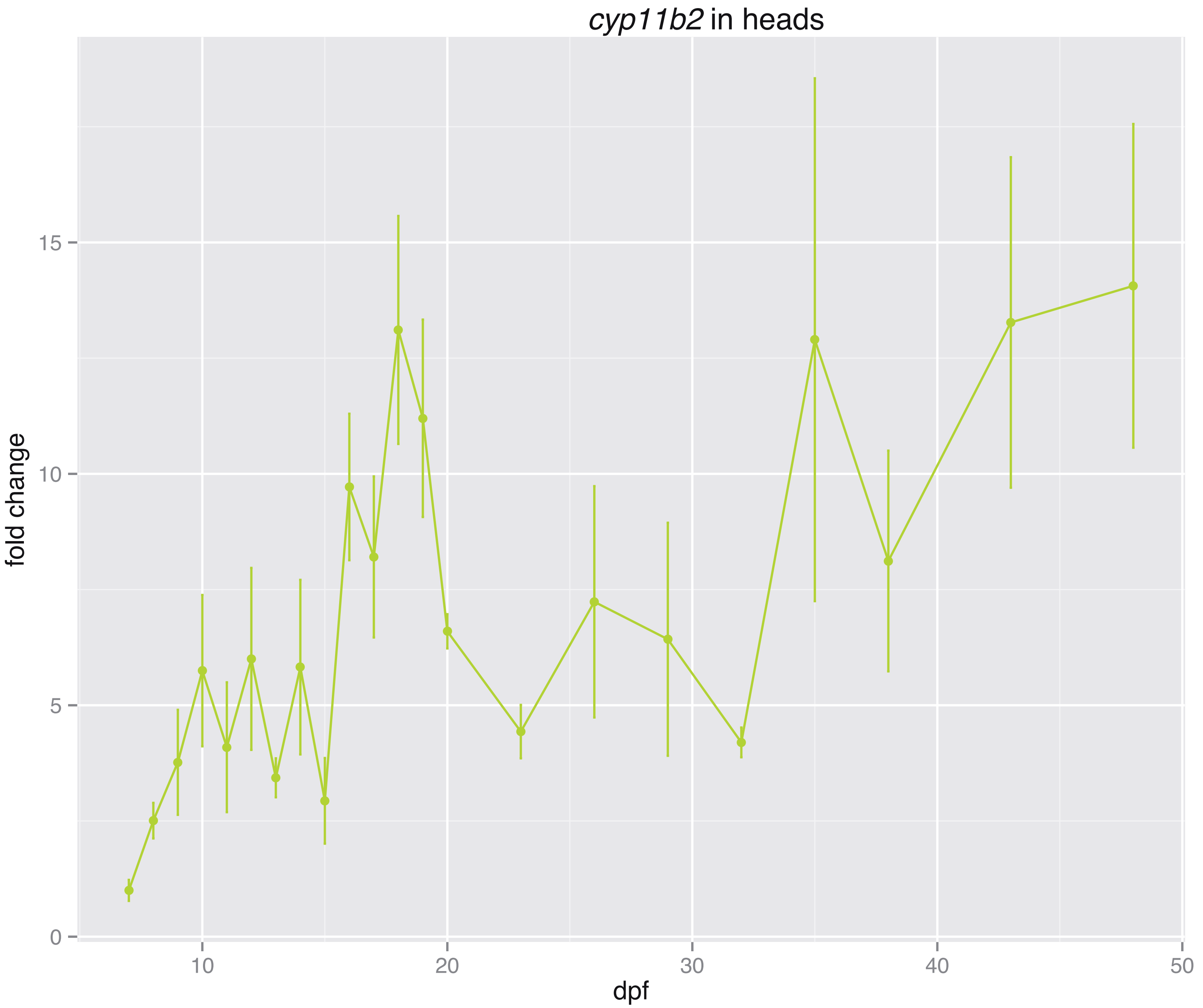

cyp11b2 in heads
fold change
dpf
0
5
10
15
10
20
30
40
50

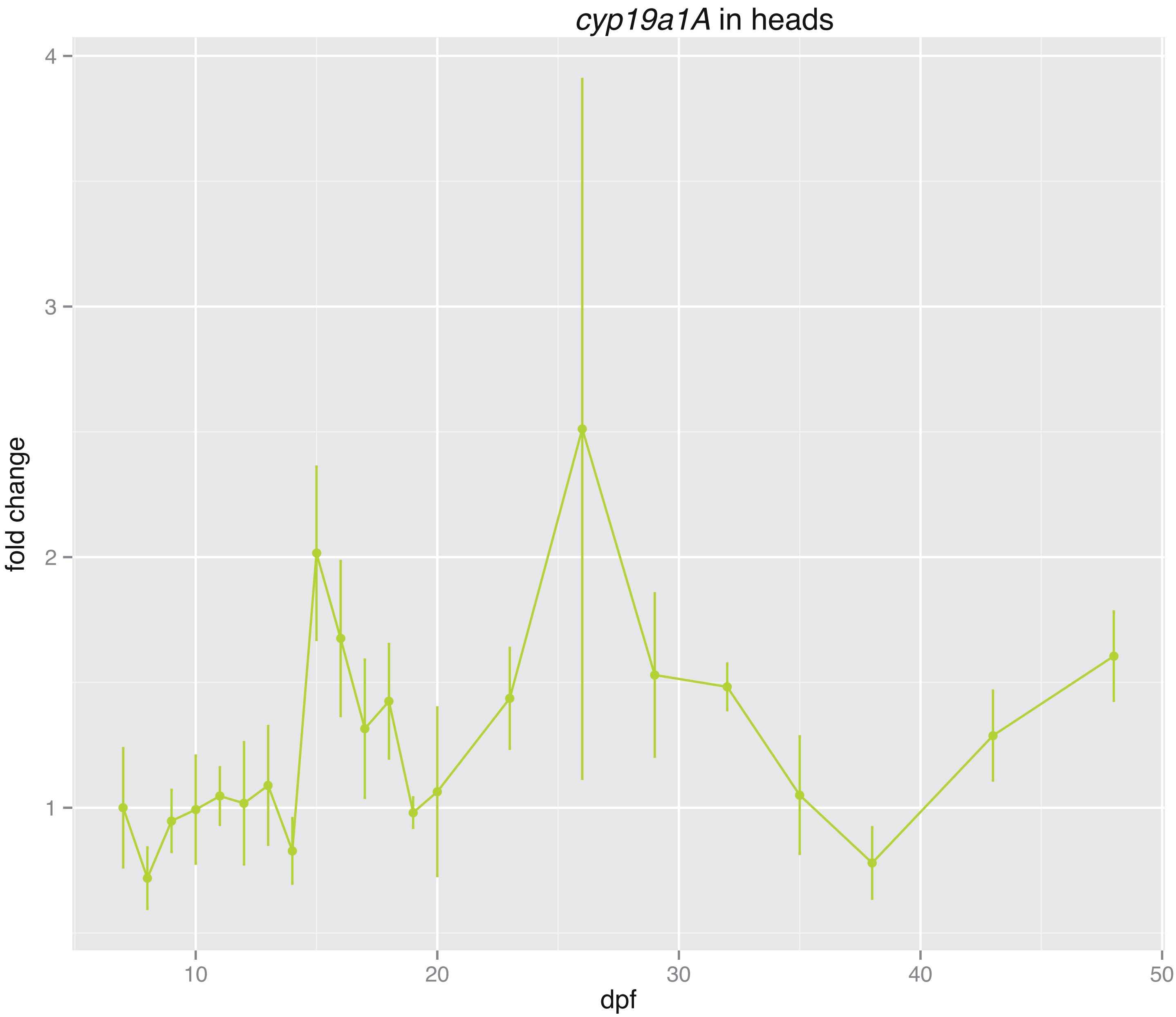

cyp19a1A in heads
fold change
4
3
2
1
10
20
30
40
50
dpf

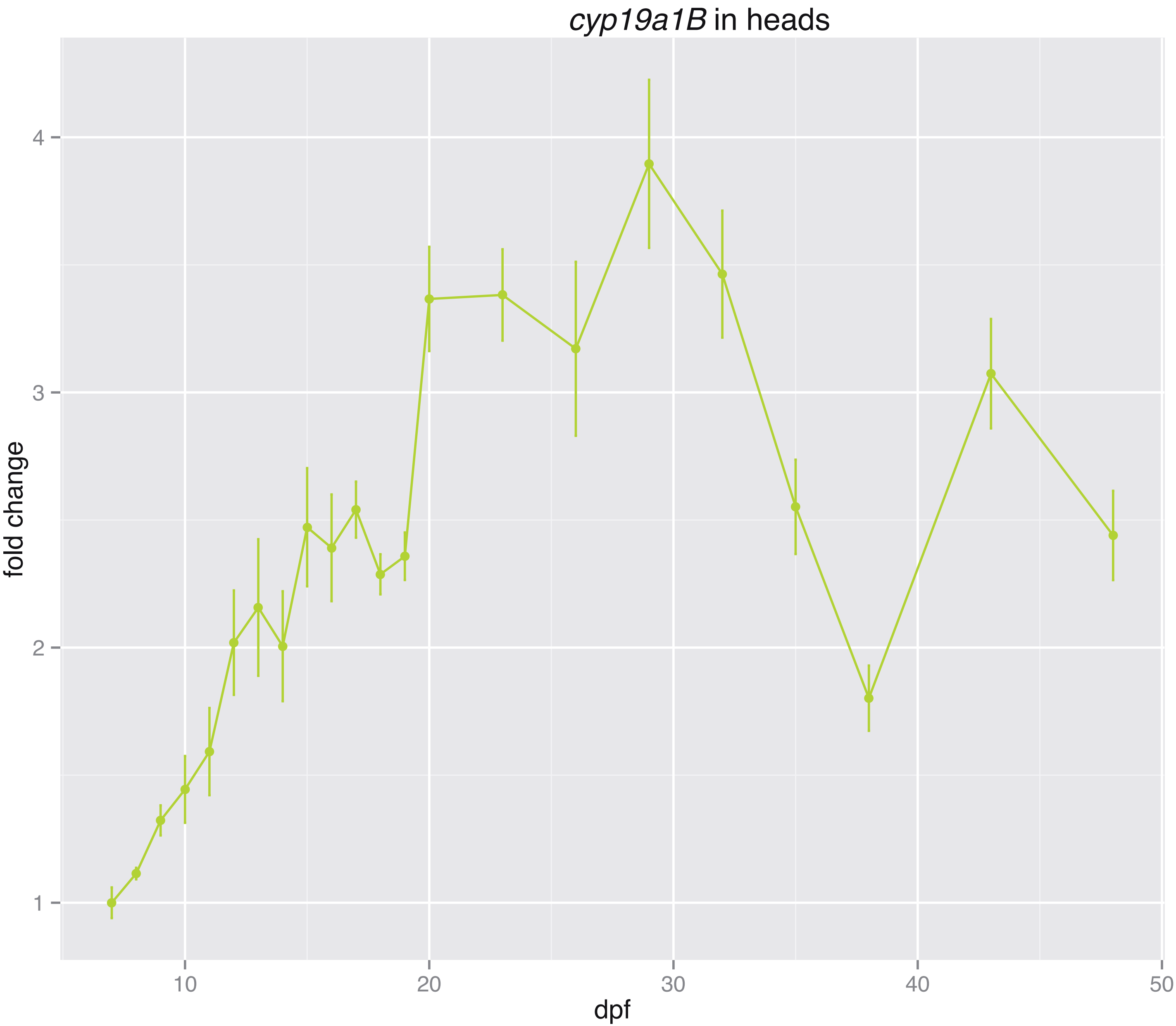

cyp19a1B in heads
fold change
dpf
1
2
3
4
10
20
30
40
50

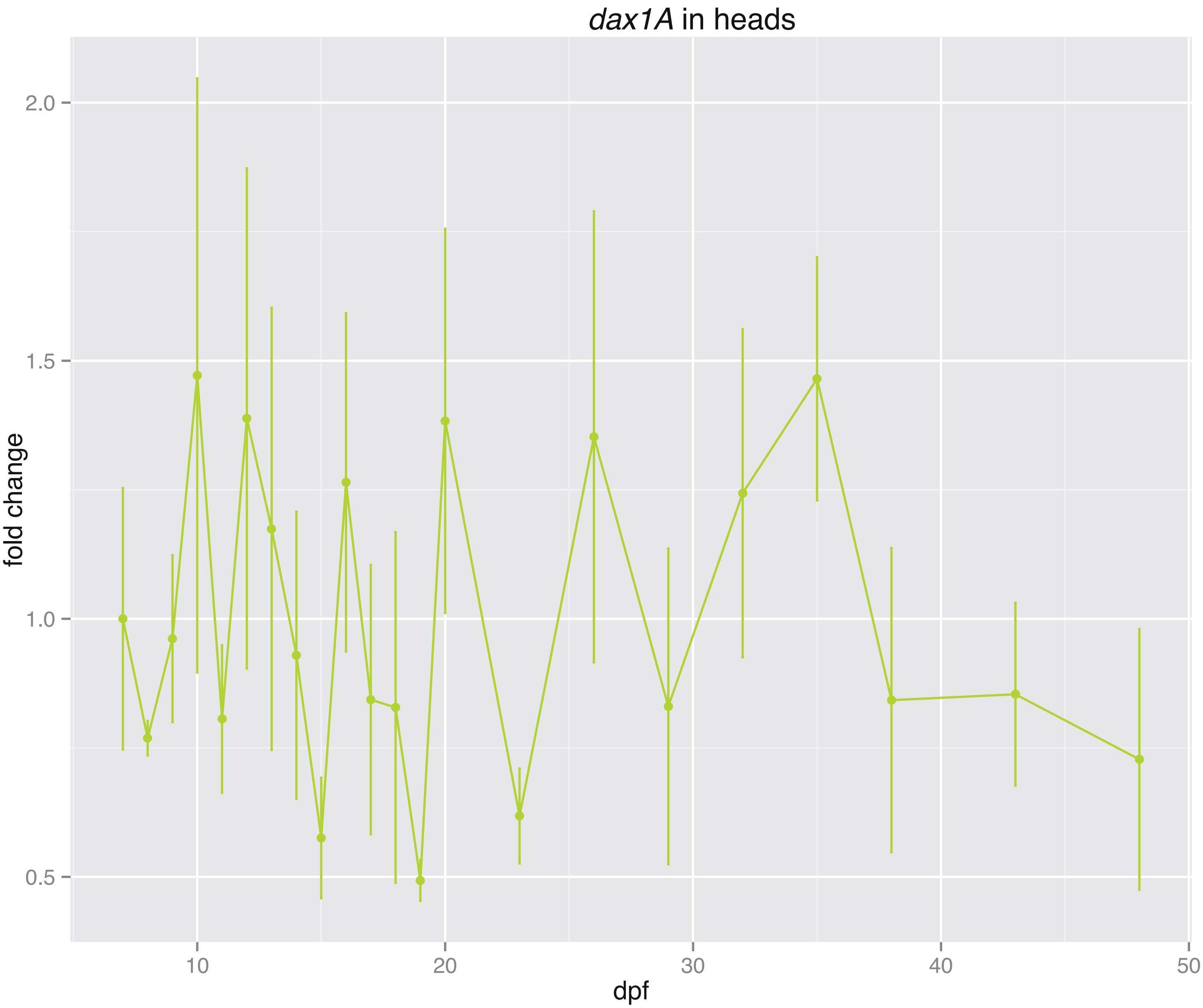

dax1A in heads
fold change
2.0
1.5
1.0
0.5
10
20
30
40
50
dpf

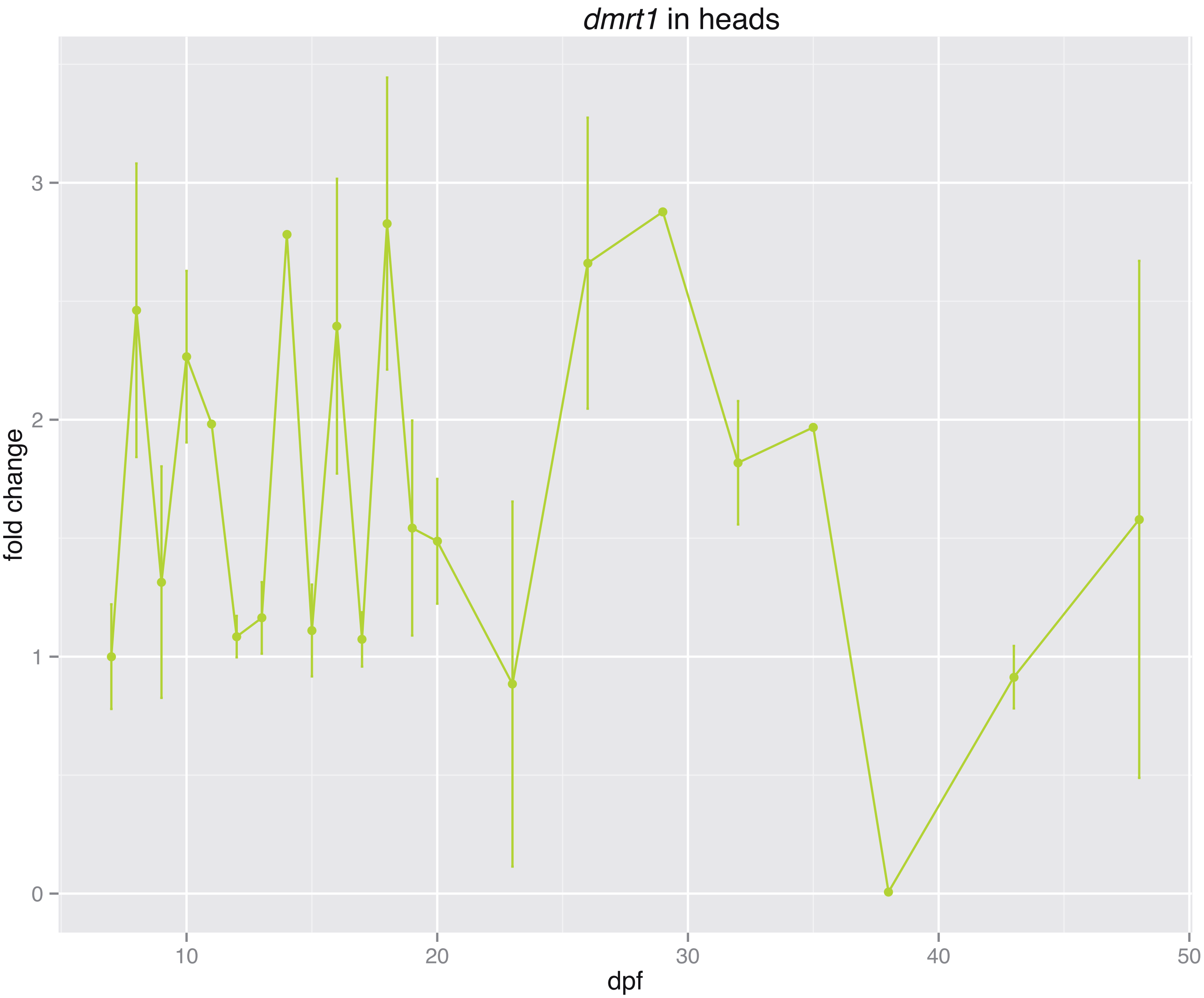

dmrt1 in heads
fold change
dpf
0
1
2
3
10
20
30
40
50

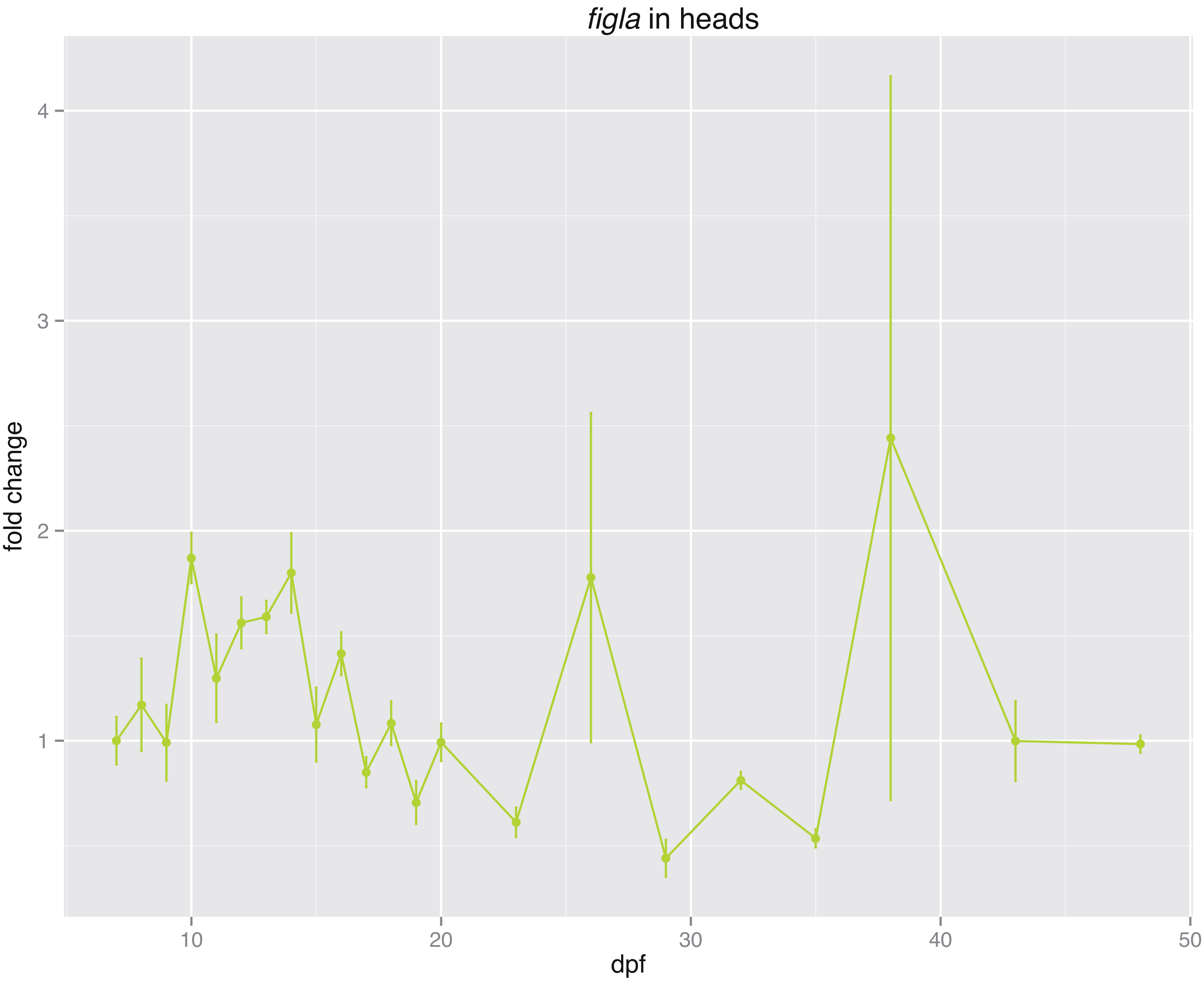

figla in heads
fold change
4
3
2
1
10
20
30
40
50
dpf

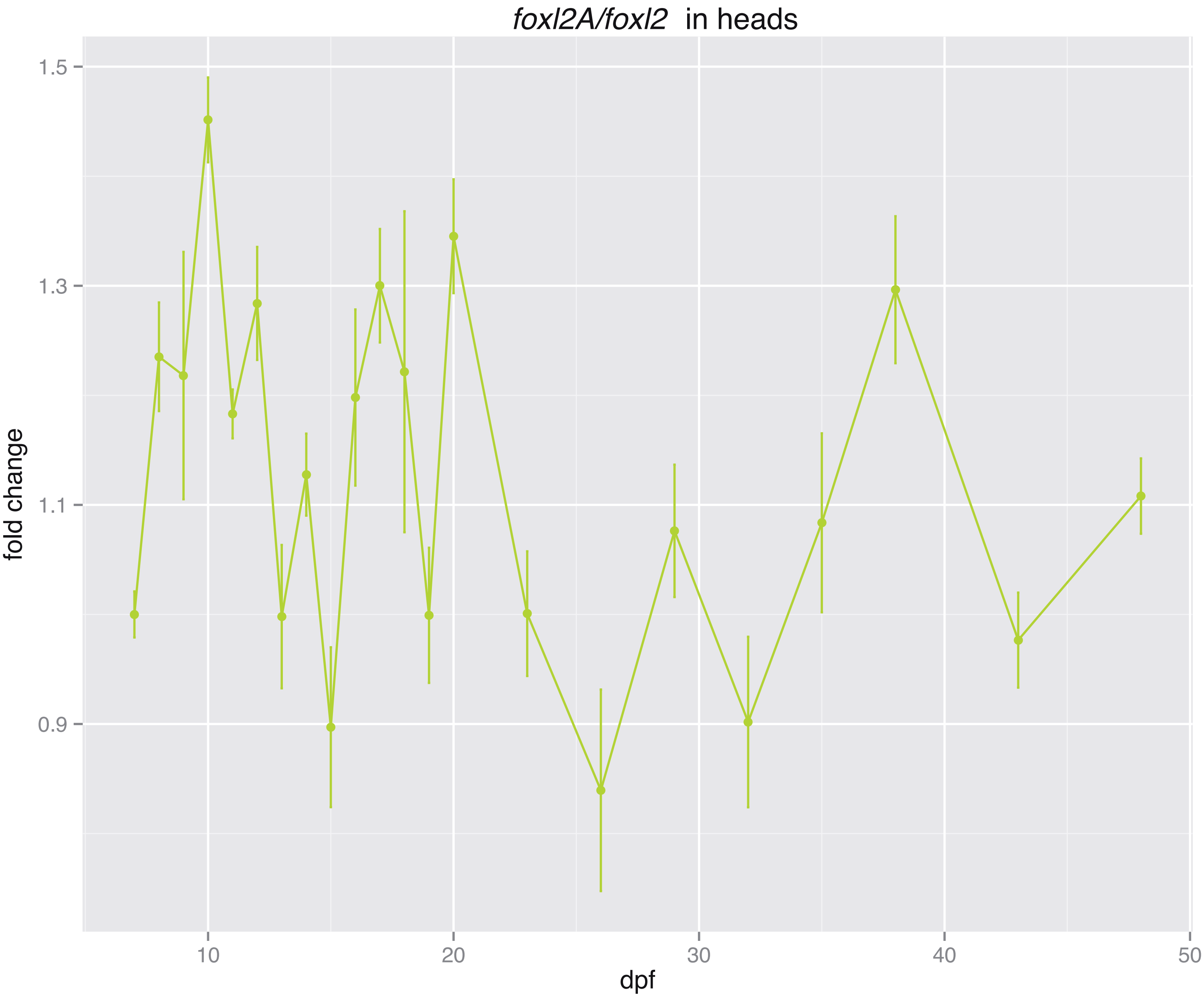

foxl2A/foxl2 in heads
fold change
dpf
1.5
1.3
1.1
0.9
10
20
30
40
50

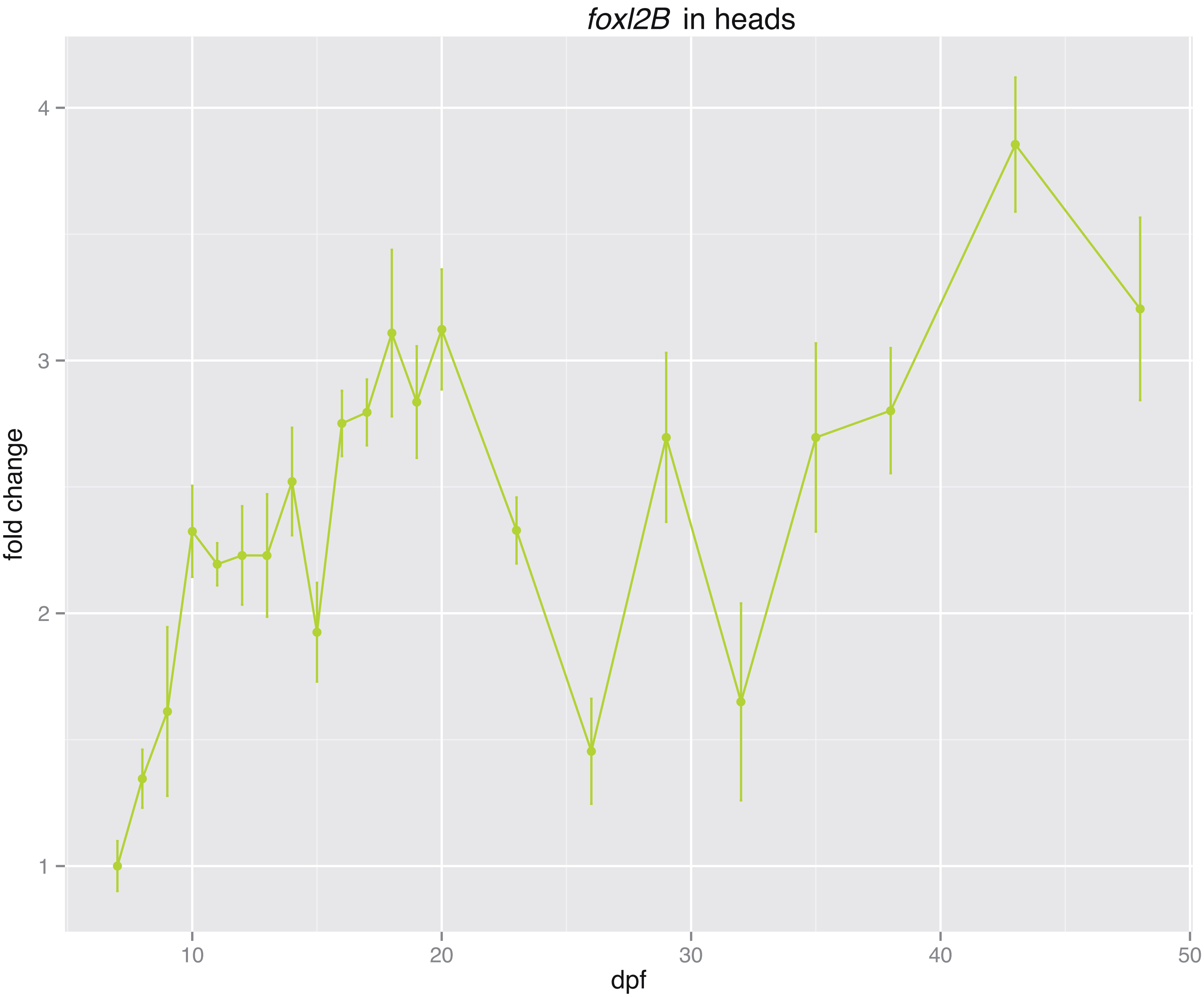

foxl2B in heads
fold change
4
3
2
1
10
20
30
40
50
dpf

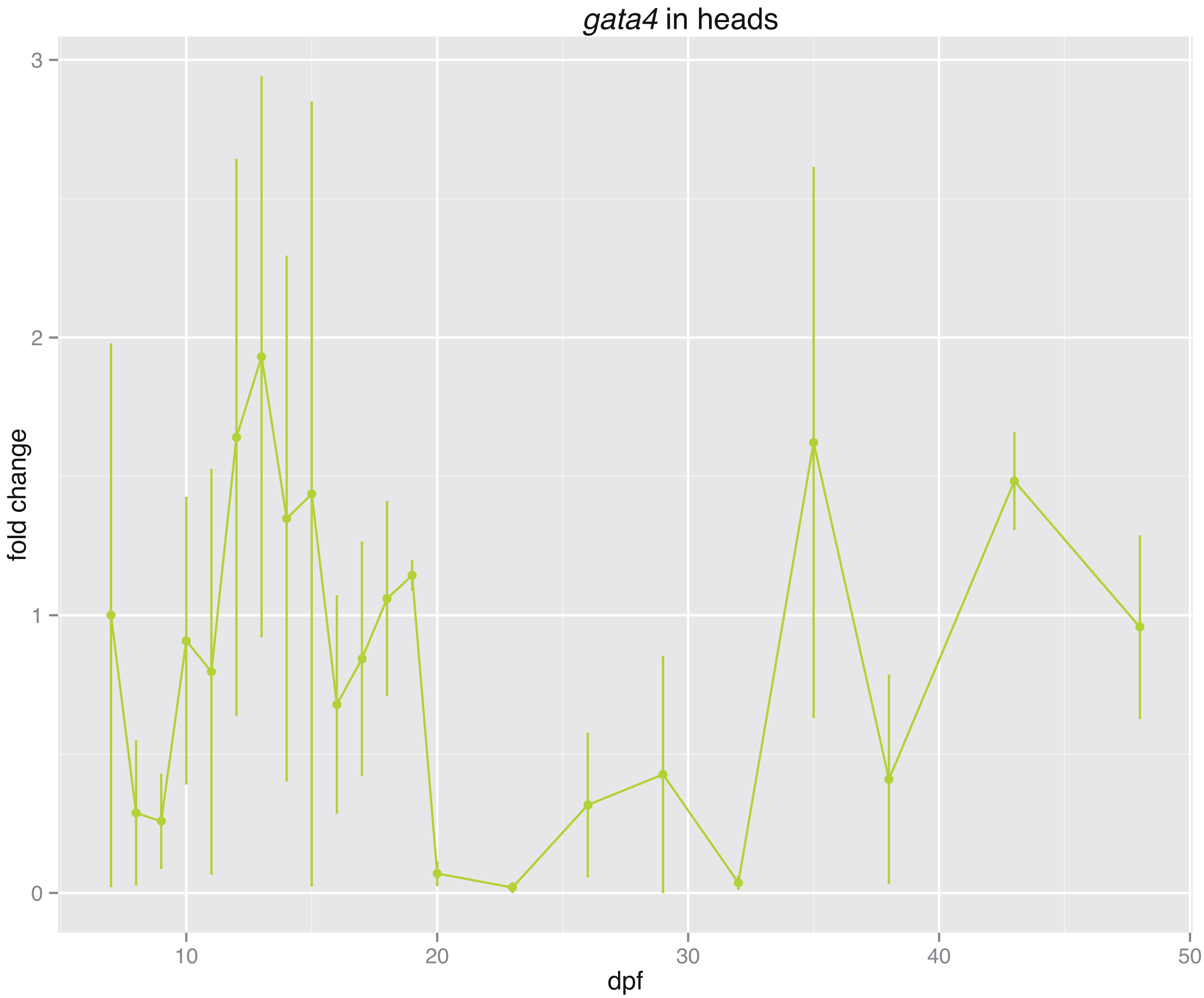
gata4 in heads
fold change
0
1
2
3
dpf
10
20
30
40
50

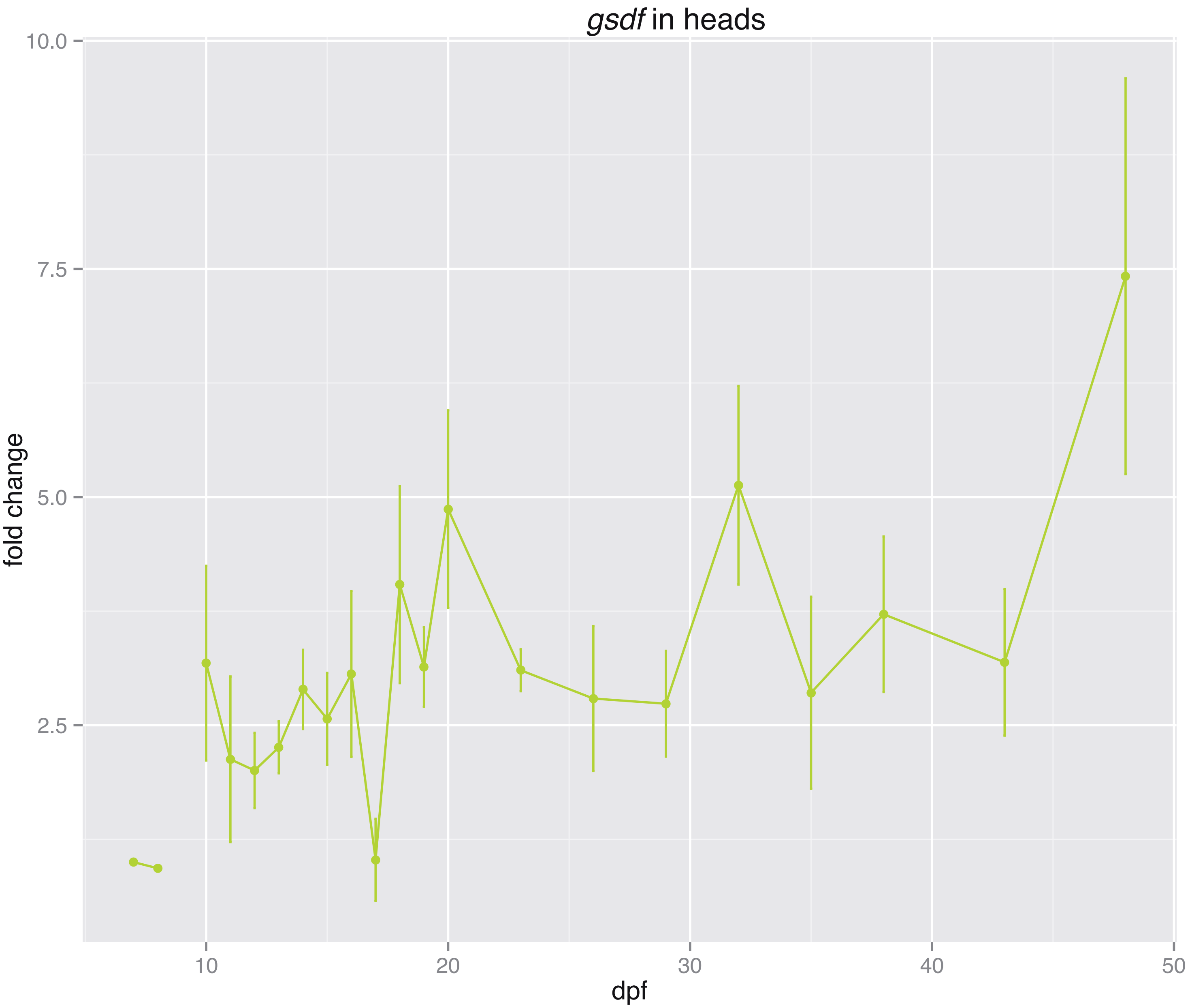
gsdf in heads
fold change
dpf
10.0
7.5
5.0
2.5
10
20
30
40
50

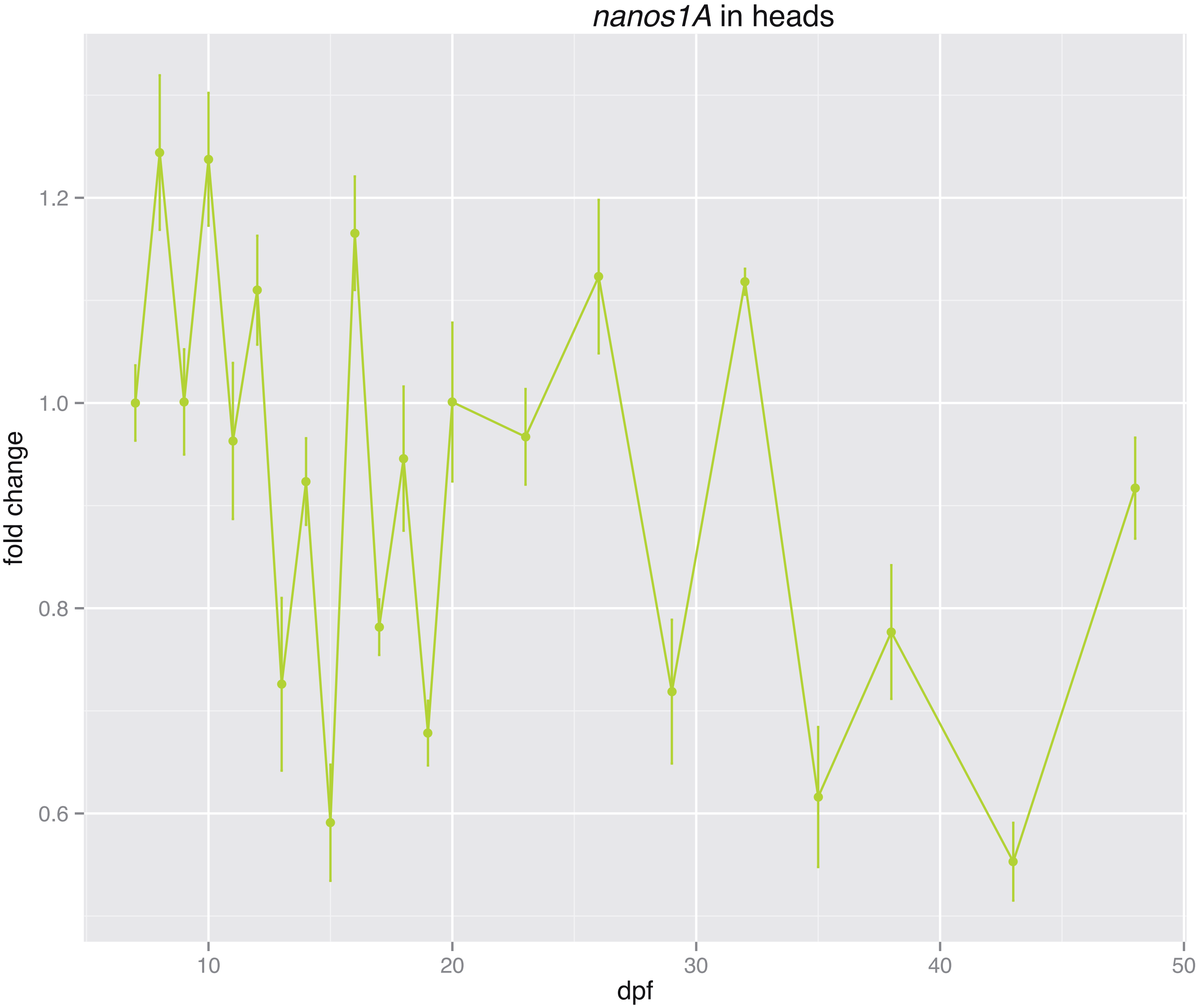

nanos1A in heads
fold change
1.2
1.0
0.8
0.6
10
20
30
40
50
dpf

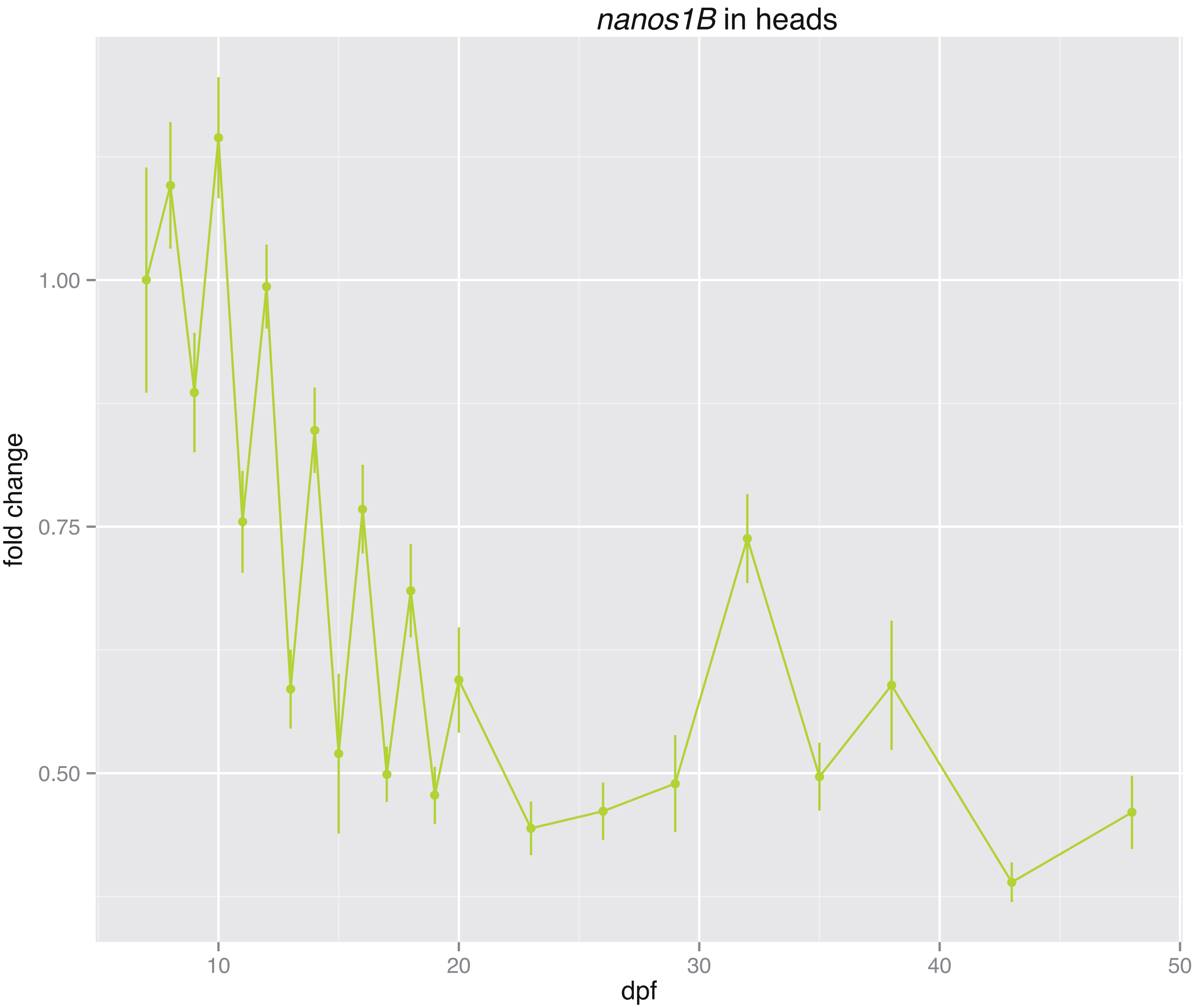

nanos1B in heads
fold change
1.00
0.75
0.50
10
20
30
40
50
dpf

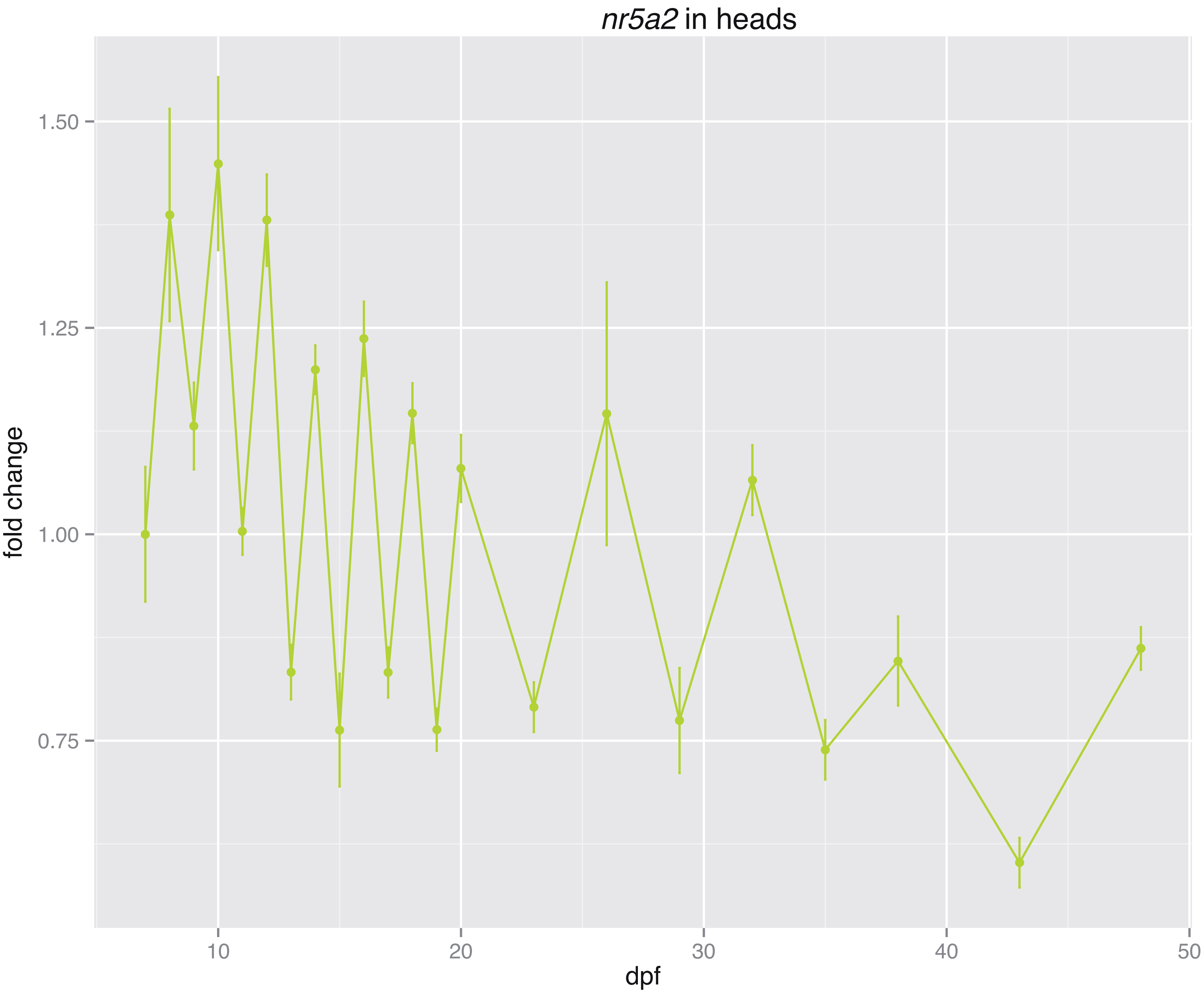

nr5a2 in heads
fold change
1.50
1.25
1.00
0.75
dpf
10
20
30
40
50

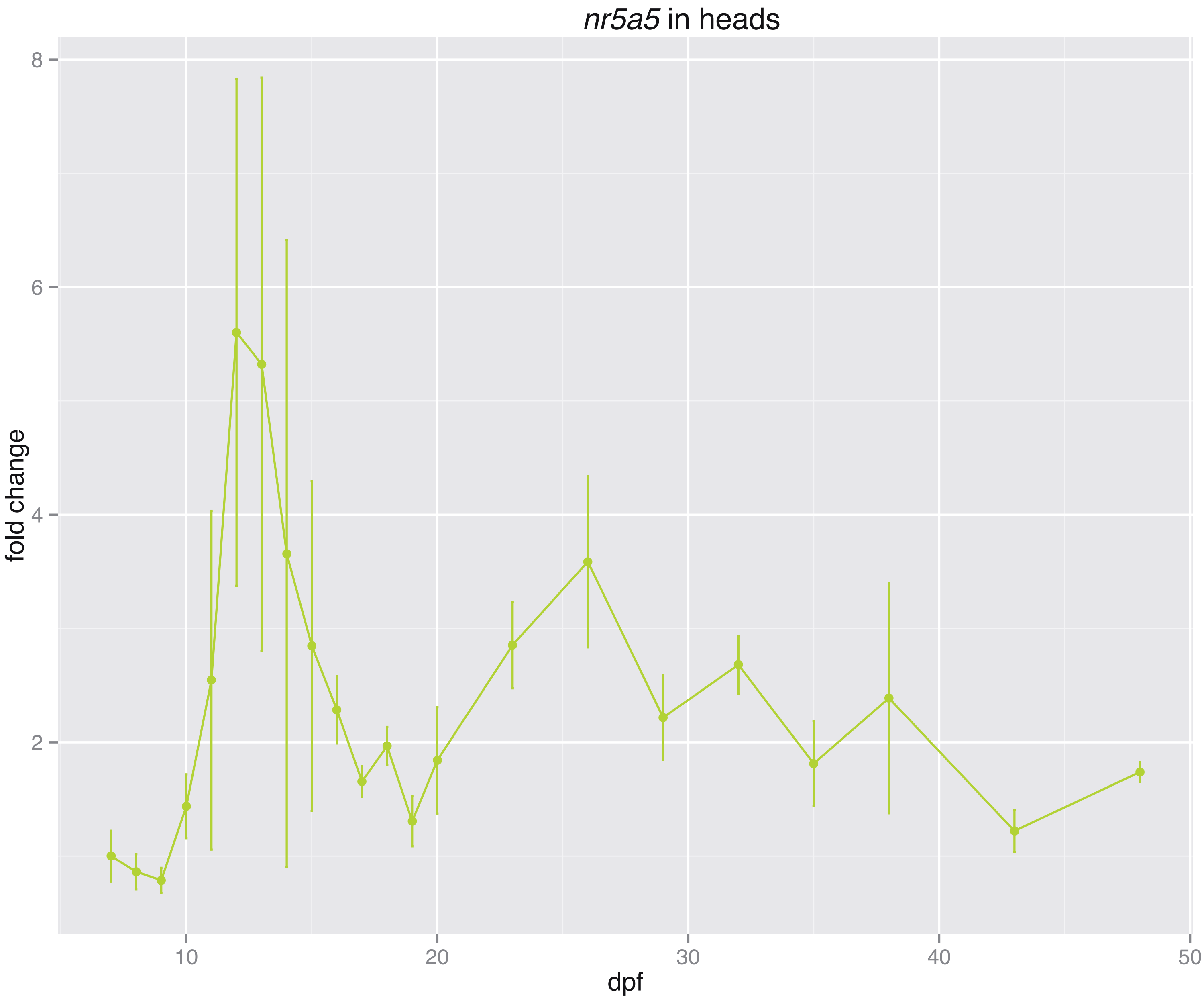
nr5a5 in heads
fold change
dpf
8
6
4
2
10
20
30
40
50

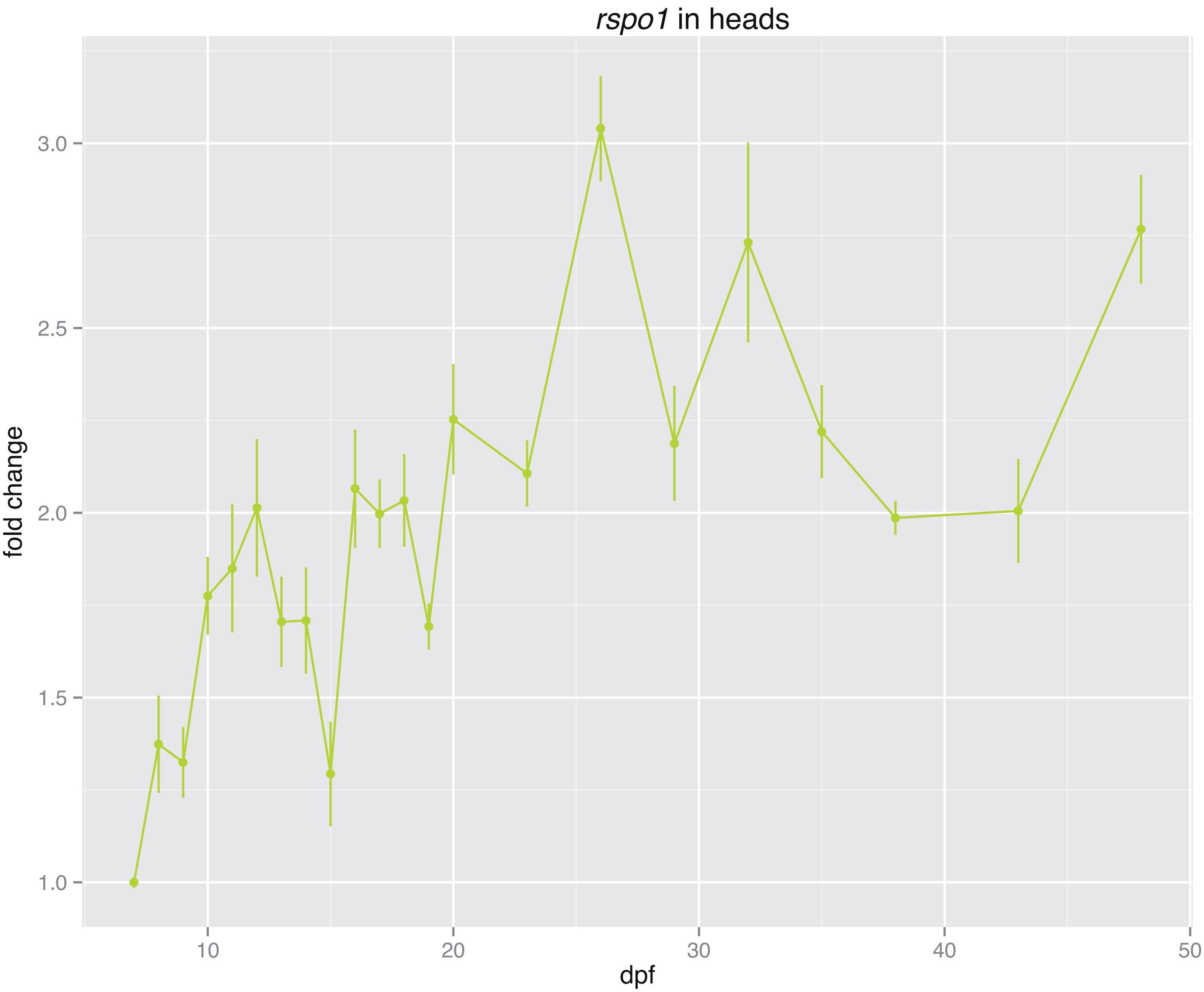

rspo1 in heads
fold change
dpf
1.0
1.5
2.0
2.5
3.0
10
20
30
40
50

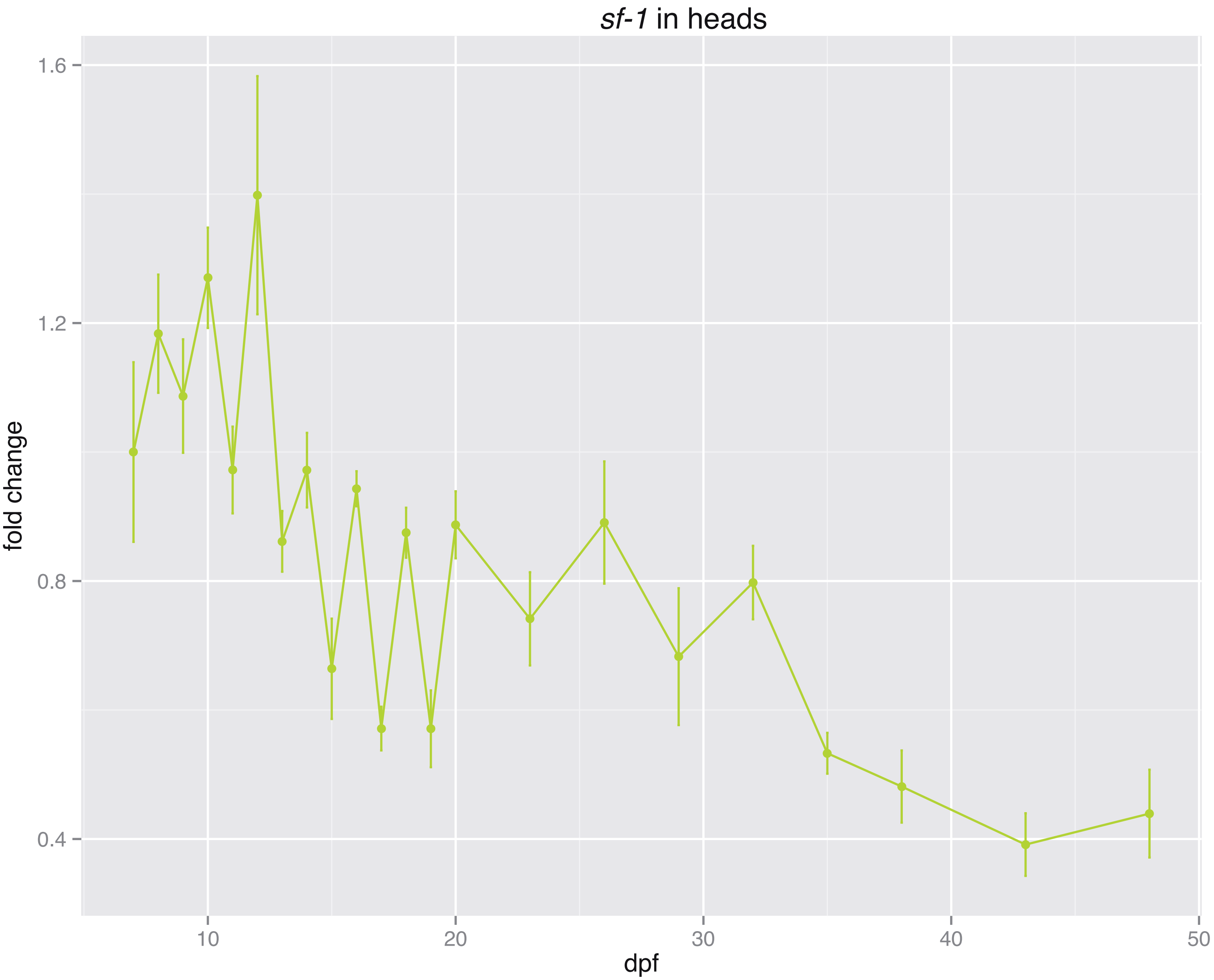

sf-1 in heads
fold change
1.6
1.2
0.8
0.4
10
20
30
40
50
dpf

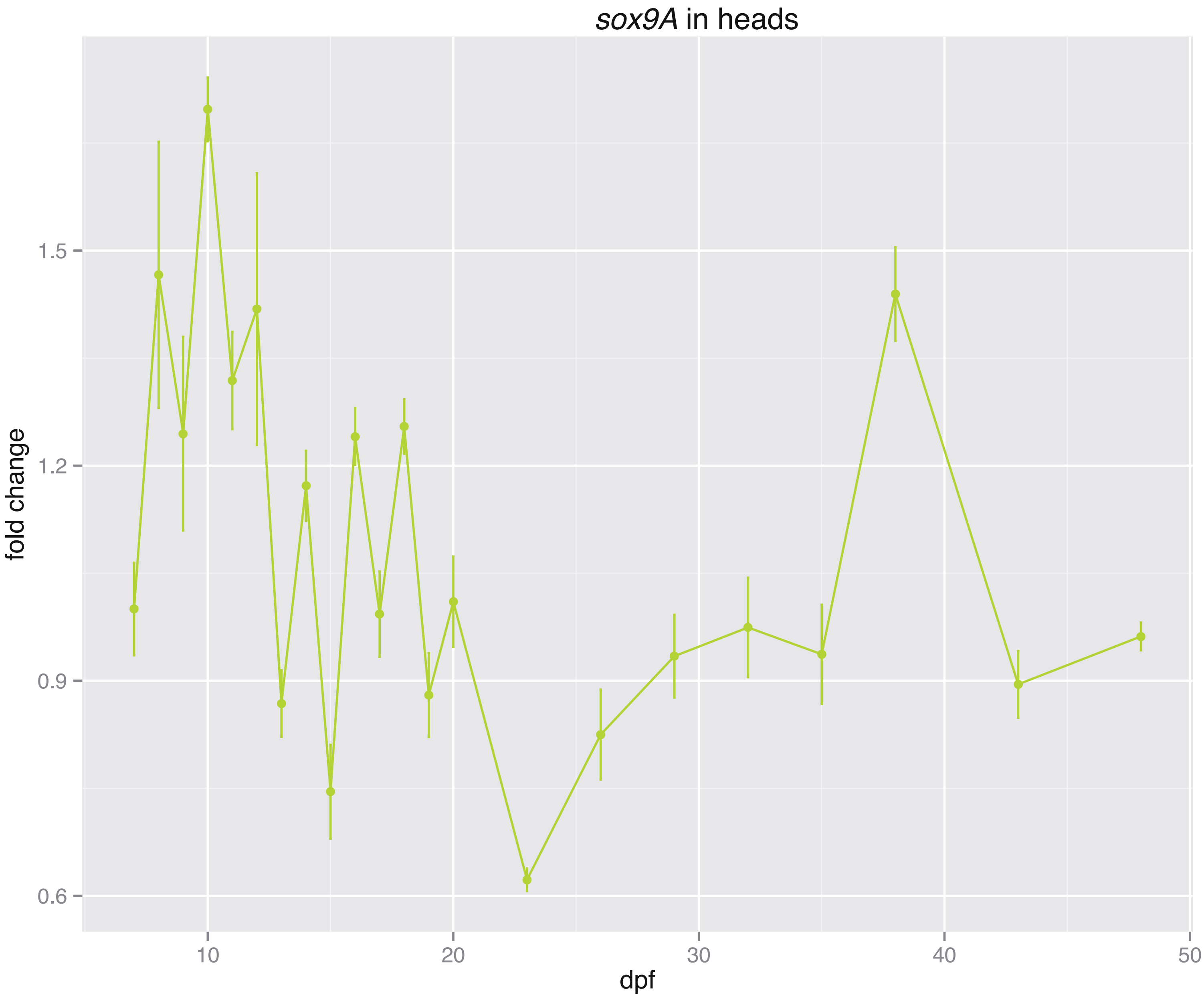
sox9A in heads
fold change
1.5
1.2
0.9
0.6
10
20
30
40
50
dpf

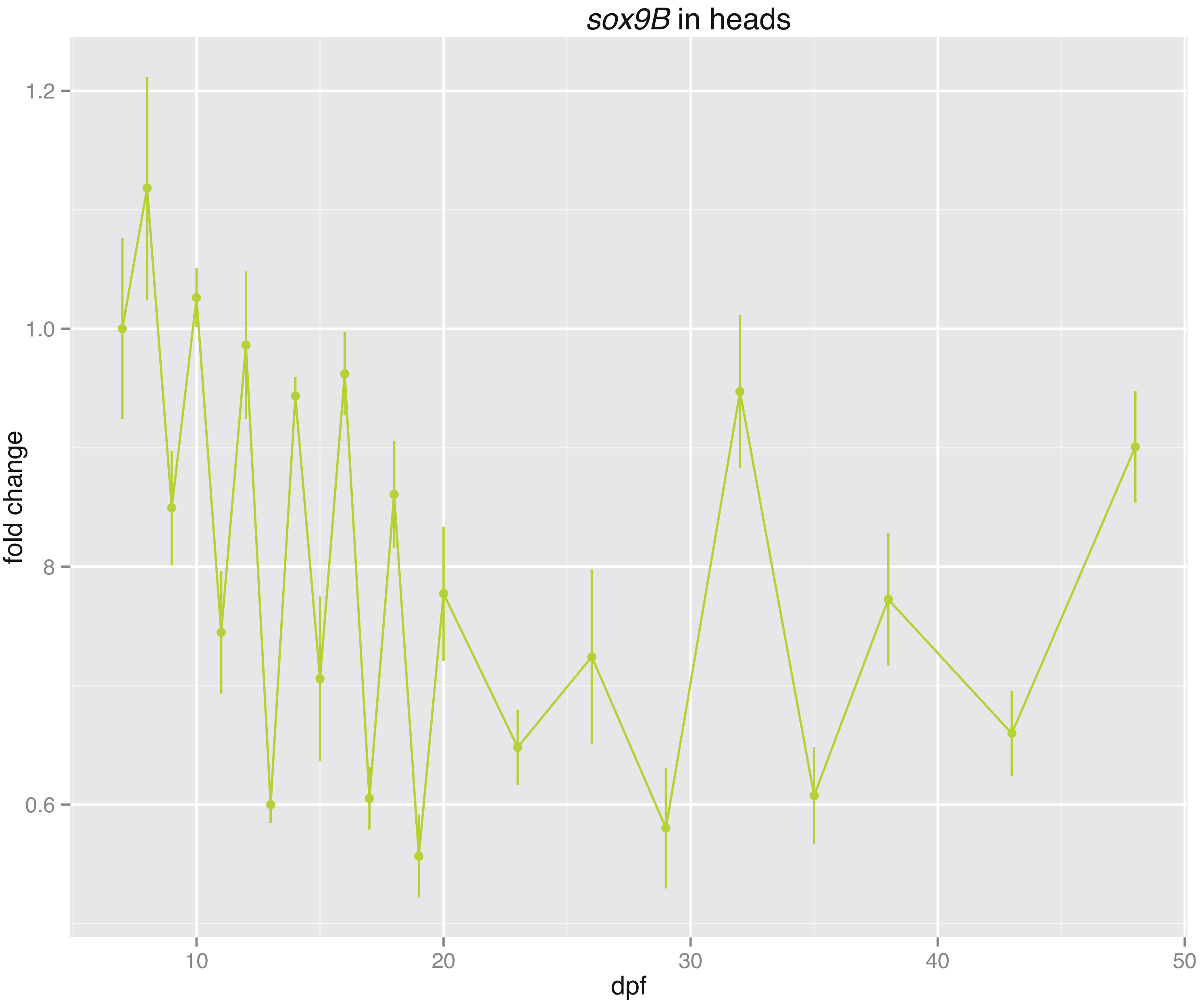

sox9B in heads
fold change
1.2
1.0
8
0.6
dpf
10
20
30
40
50

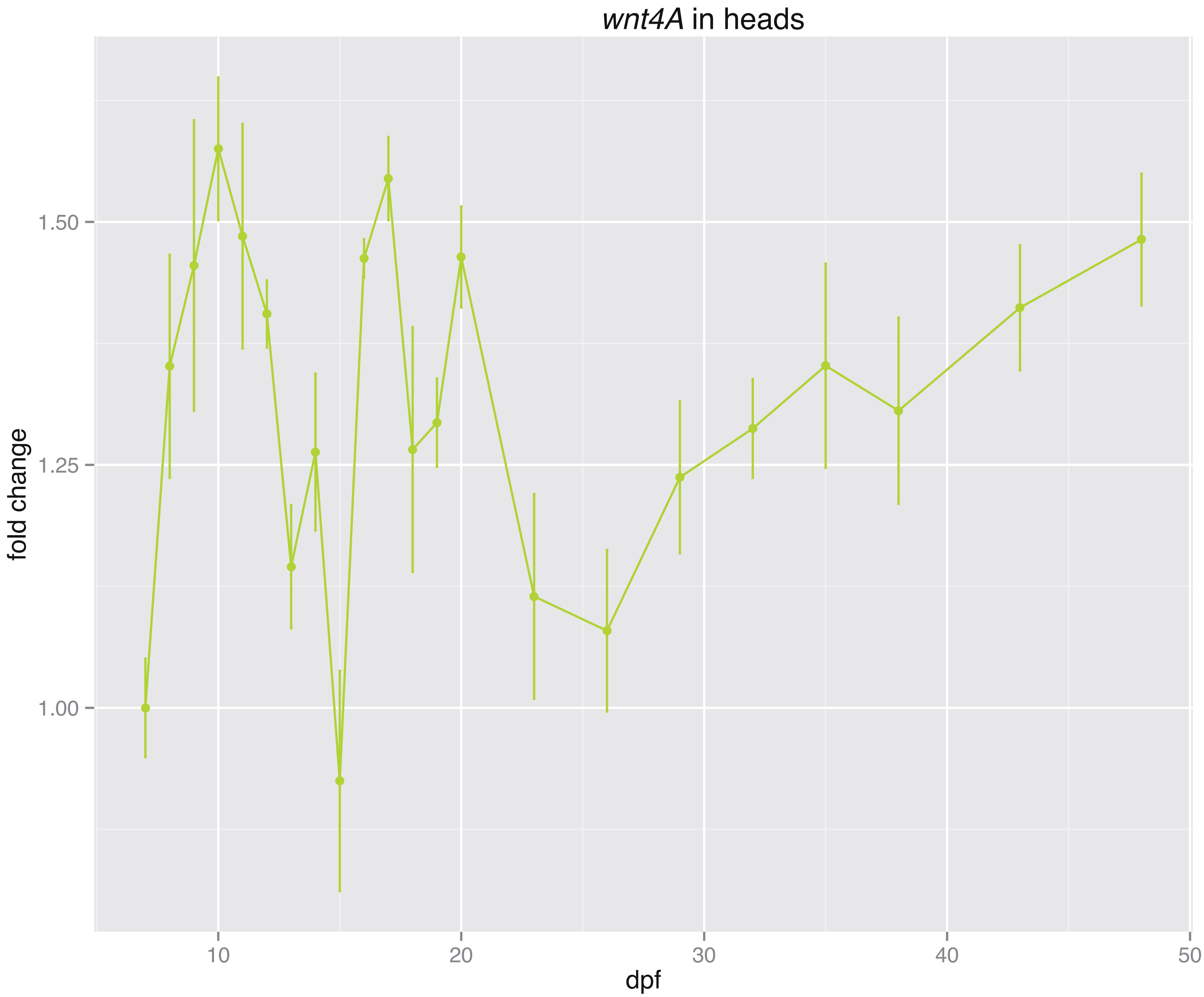

wnt4A in heads
fold change
1.50
1.25
1.00
dpf
10
20
30
40
50

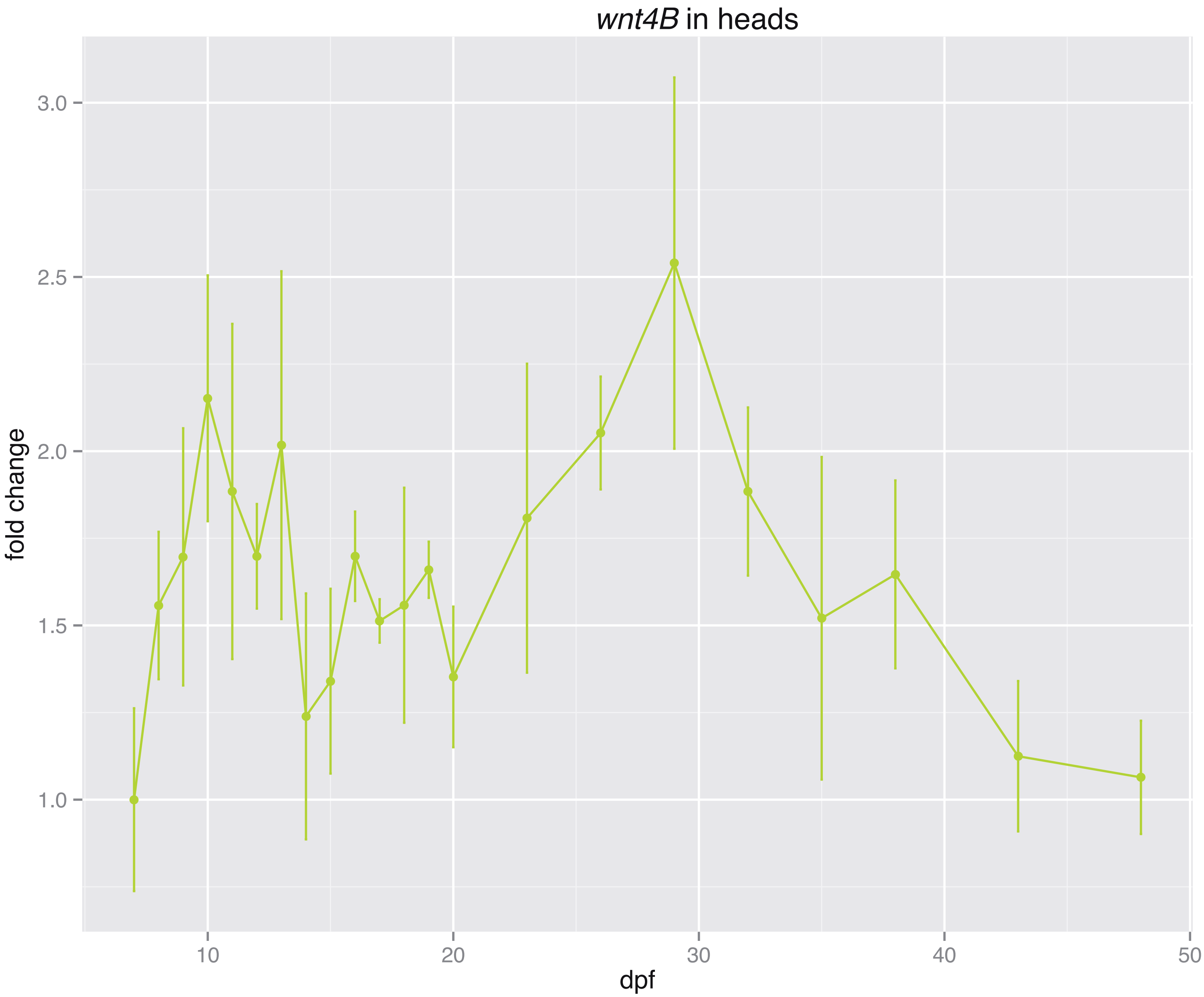

wnt4B in heads
3.0
2.5
2.0
1.5
1.0
fold change
10
20
30
40
50
dpf

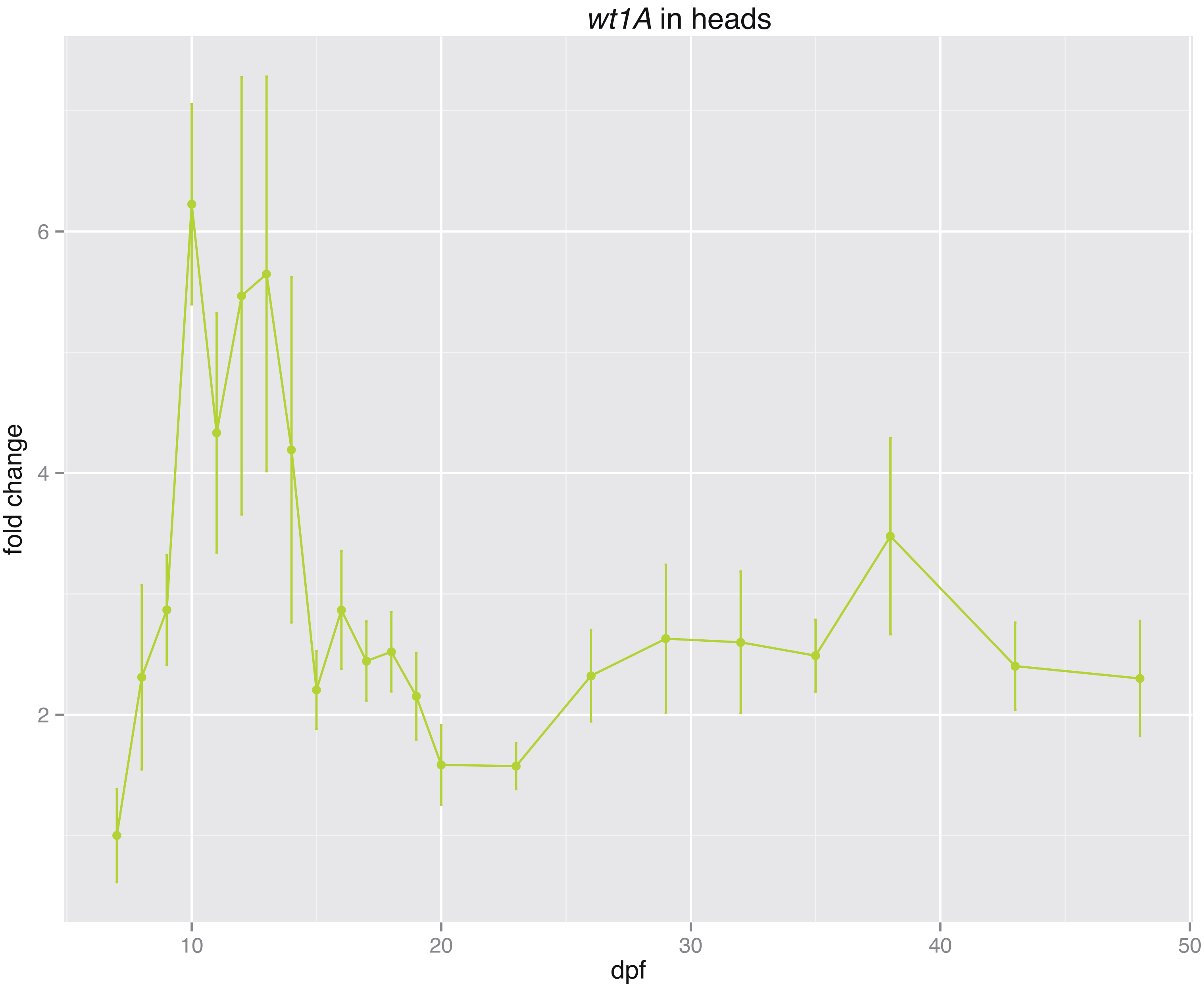

wt1A in heads
fold change
dpf
2
4
6
10
20
30
40
50

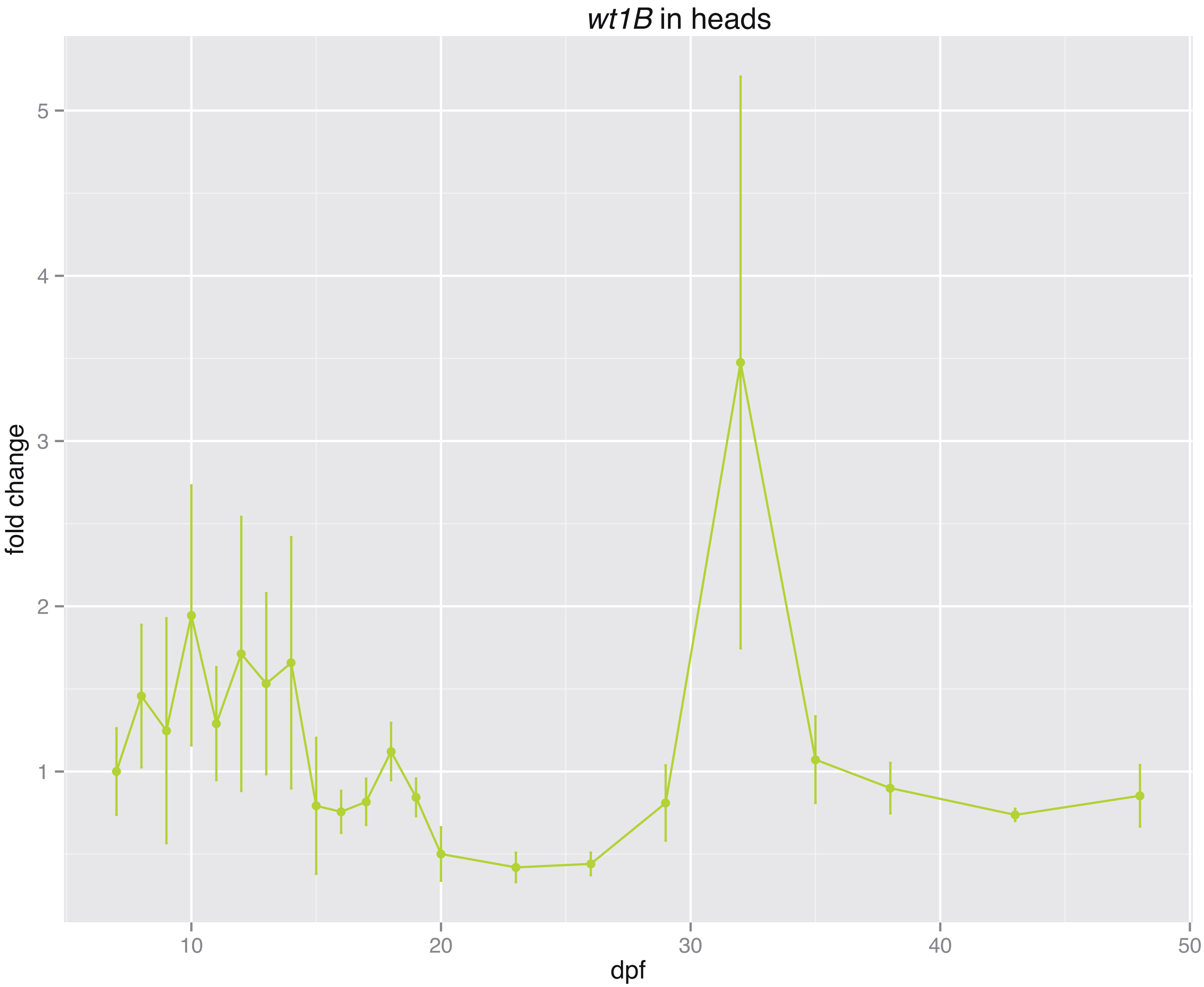

wt1B in heads
fold change
dpf
1
2
3
4
5
10
20
30
40
50

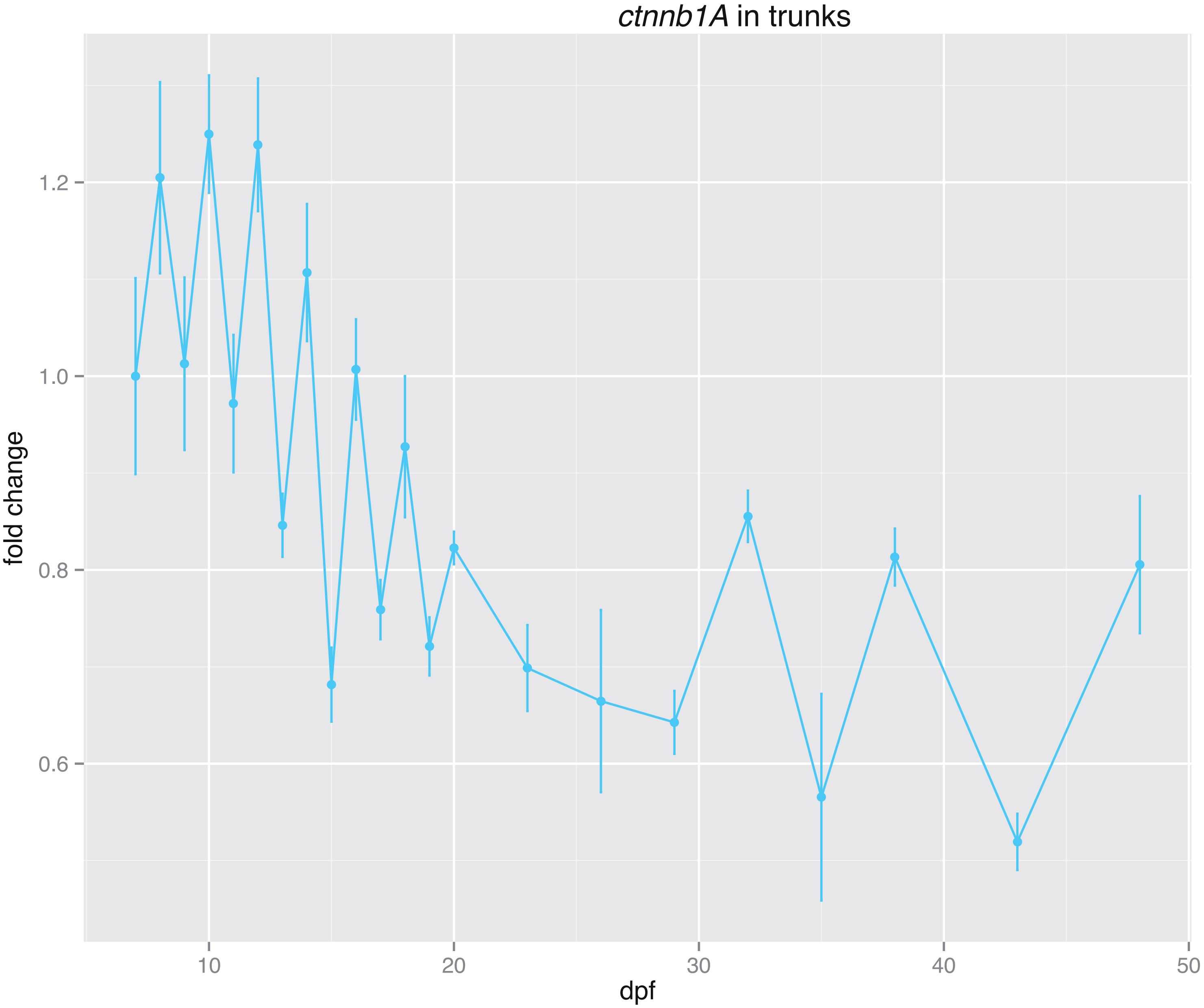

ctnnb1A in trunks
fold change
1.2
1.0
0.8
0.6
10
20
30
40
50
dpf

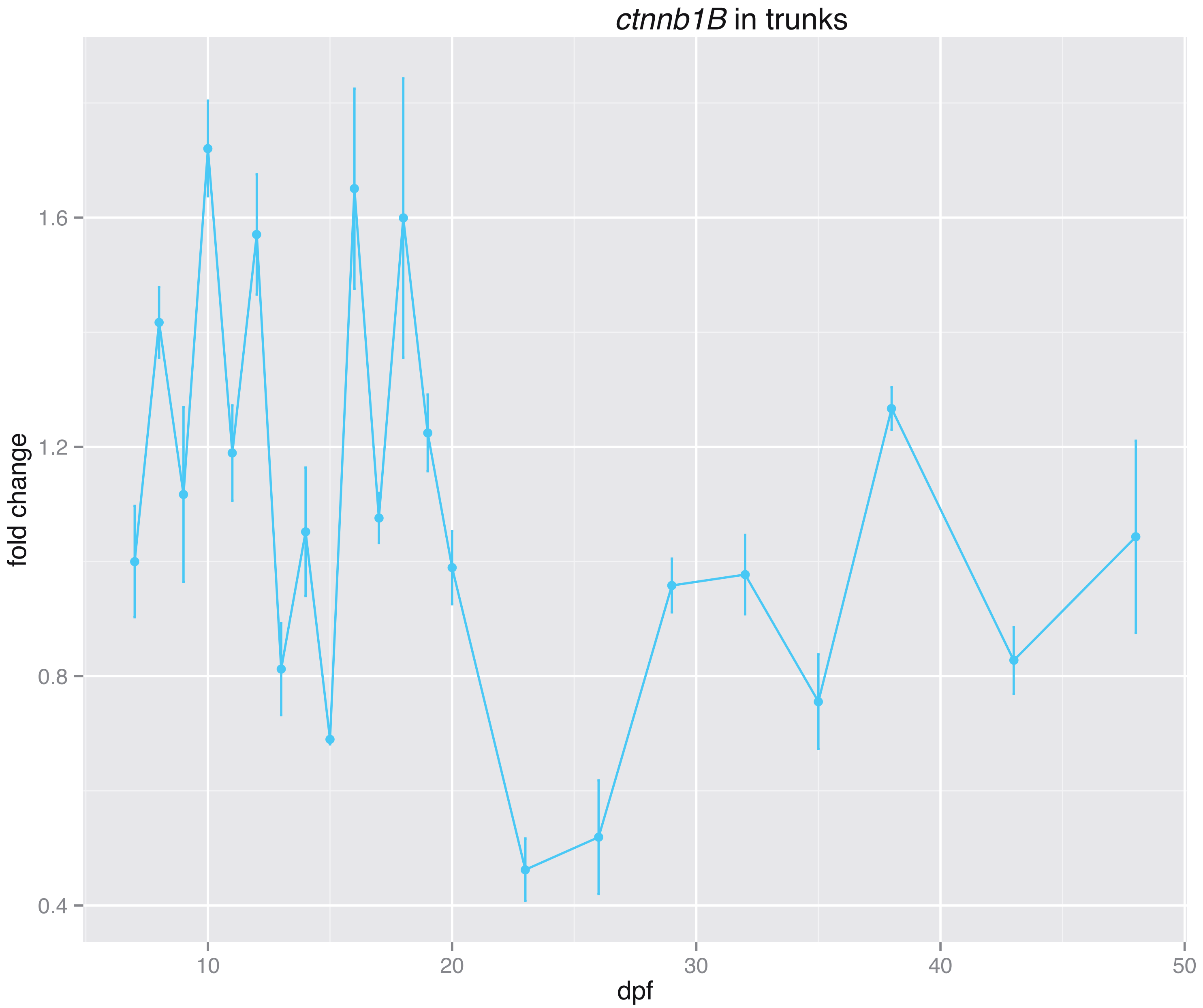

ctnnb1B in trunks
fold change
1.6
1.2
0.8
0.4
10
20
30
40
50
dpf

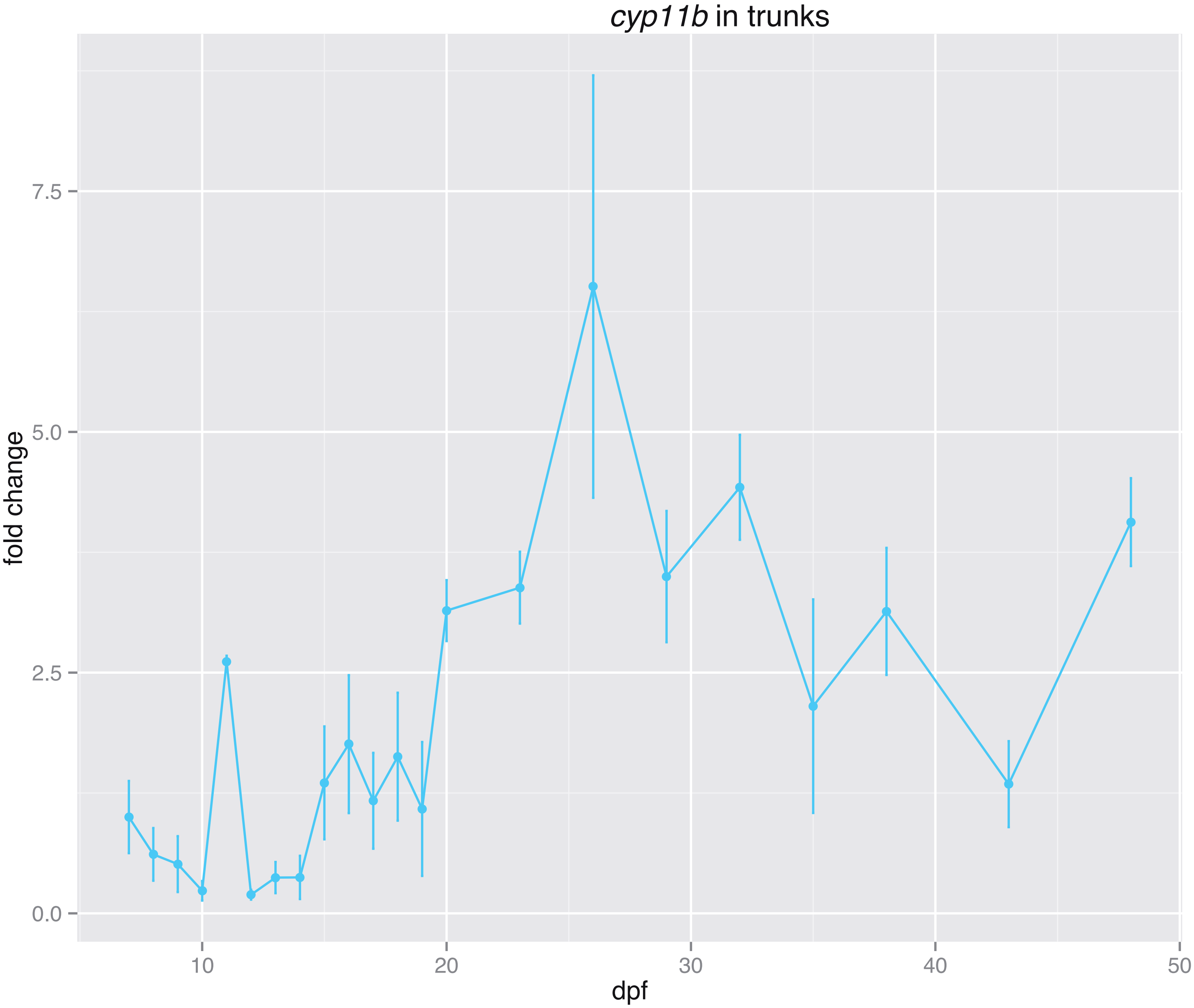

cyp11b in trunks
fold change
7.5
5.0
2.5
0.0
10
20
30
40
50
dpf

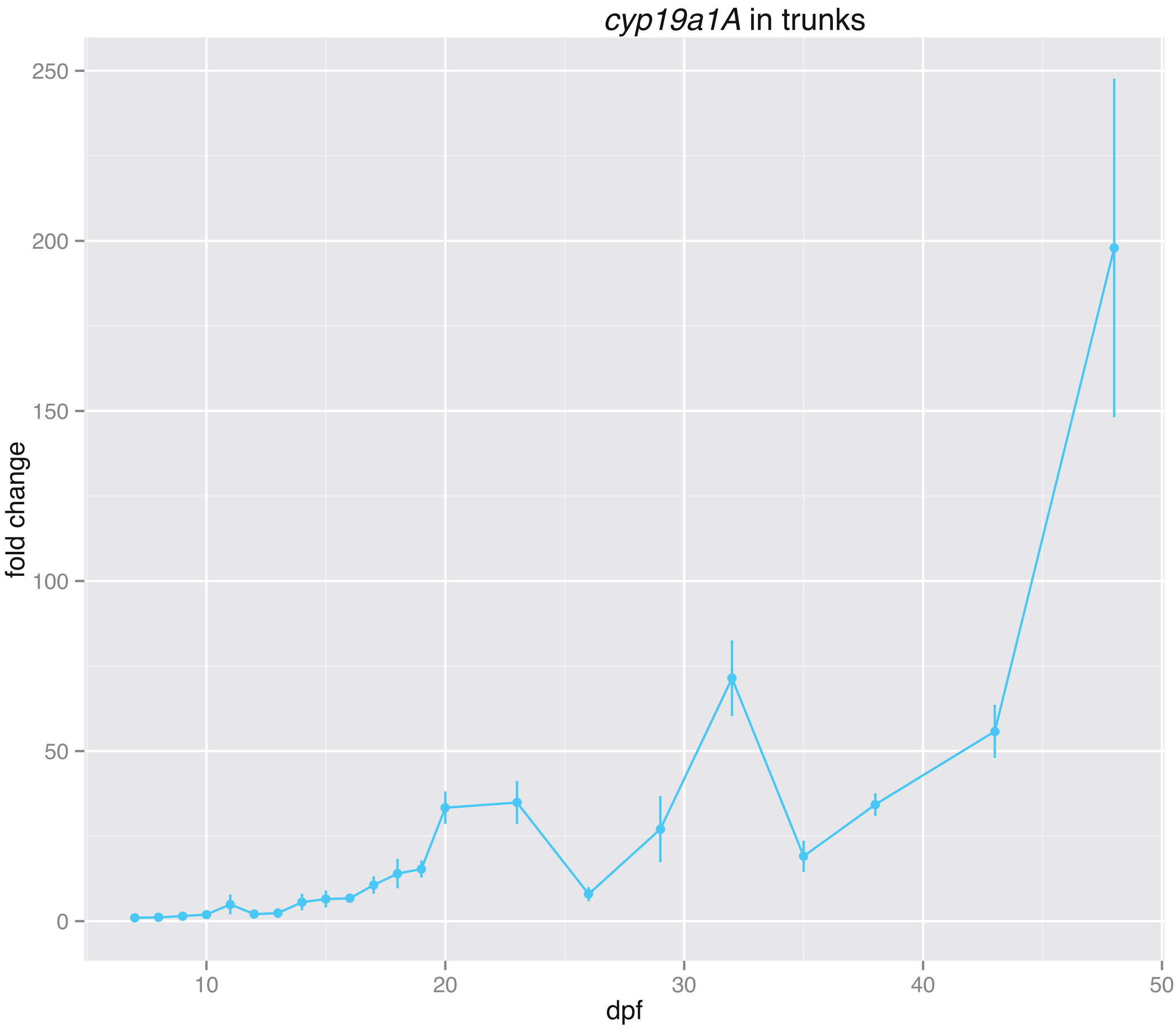

cyp19a1A in trunks
fold change
250
200
150
100
50
0
10
20
30
40
50
dpf

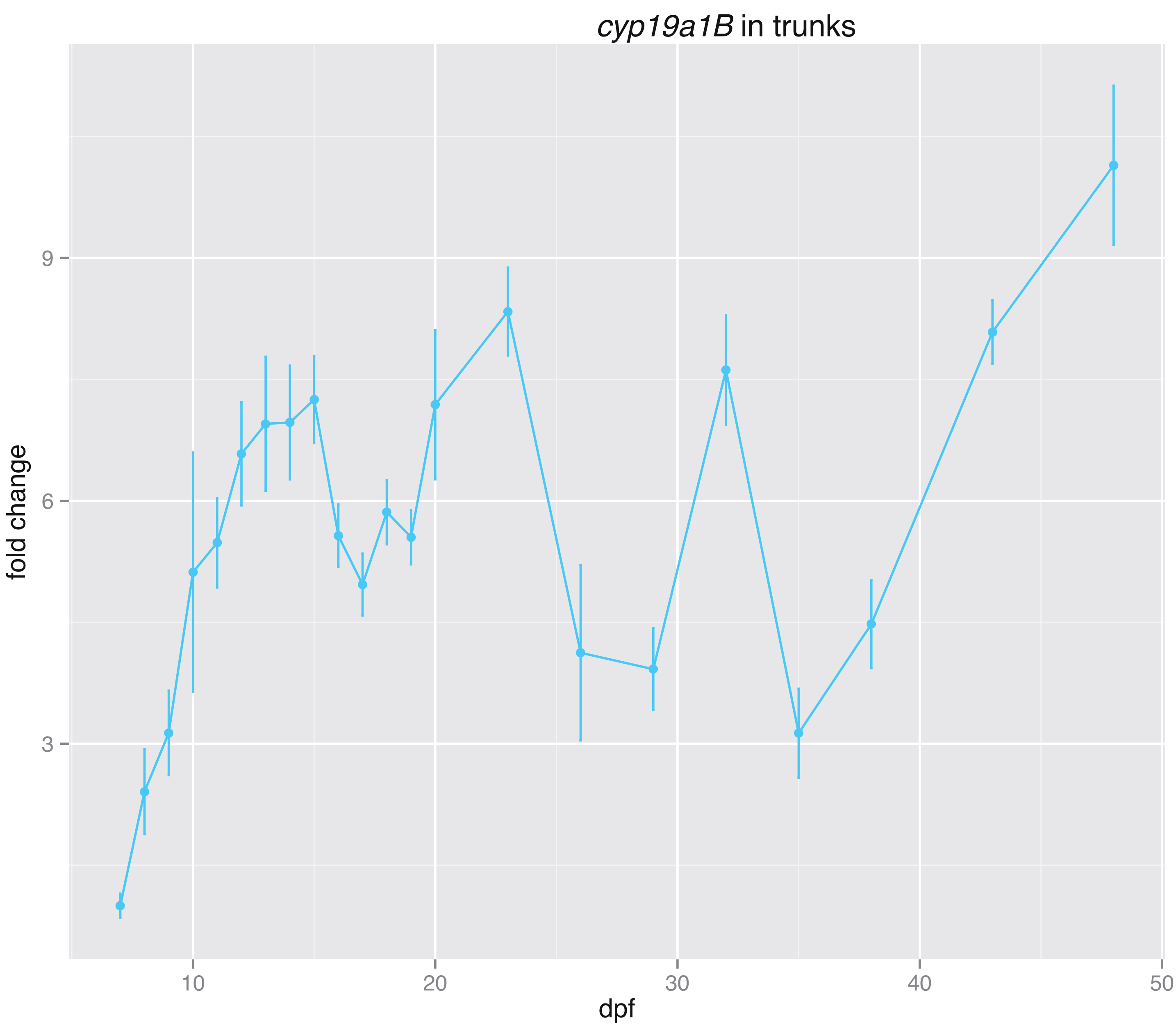

cyp19a1B in trunks
fold change
9
6
3
10
20
30
40
50
dpf

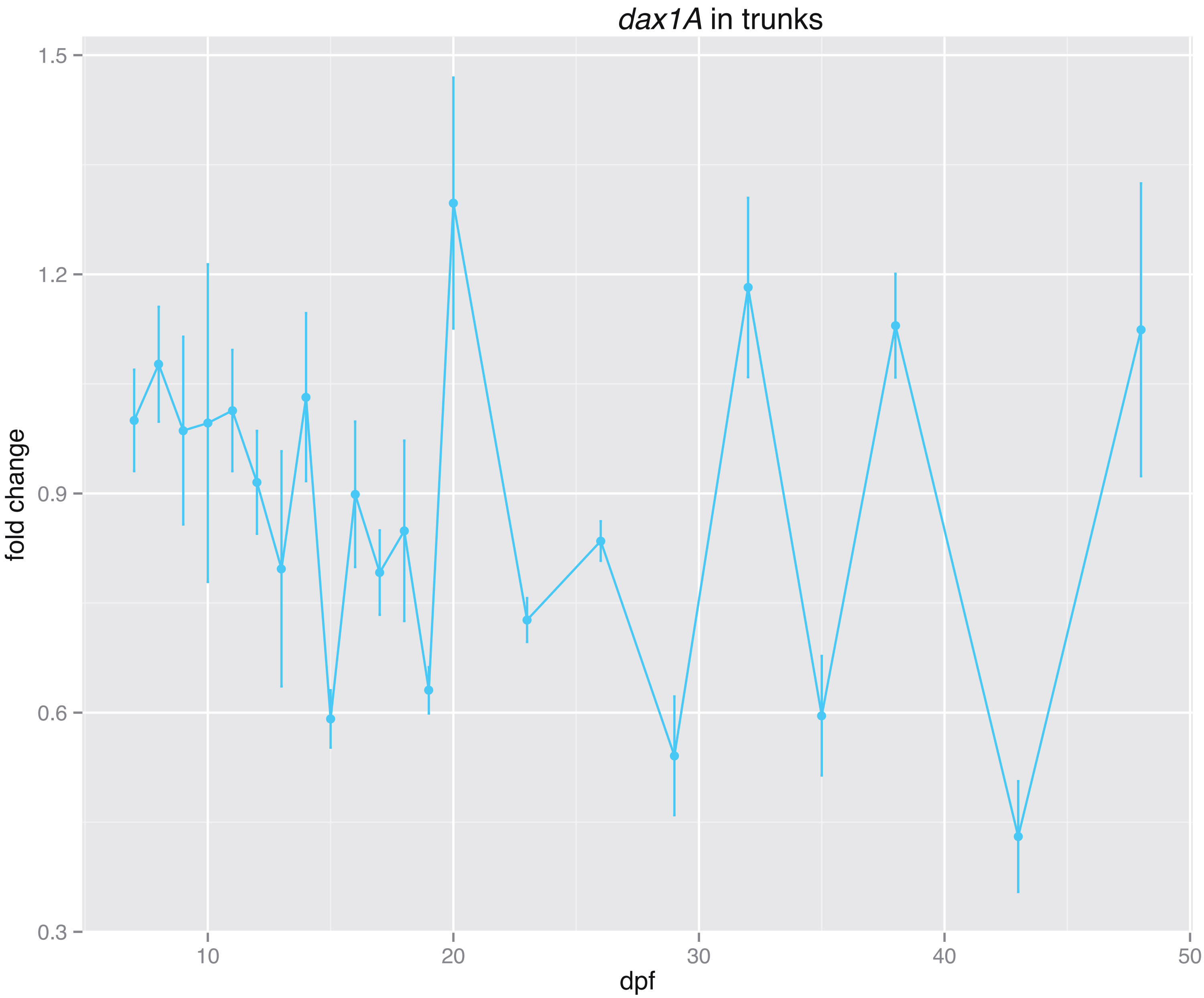

dax1A in trunks
fold change
dpf
1.5
1.2
0.9
0.6
0.3
10
20
30
40
50

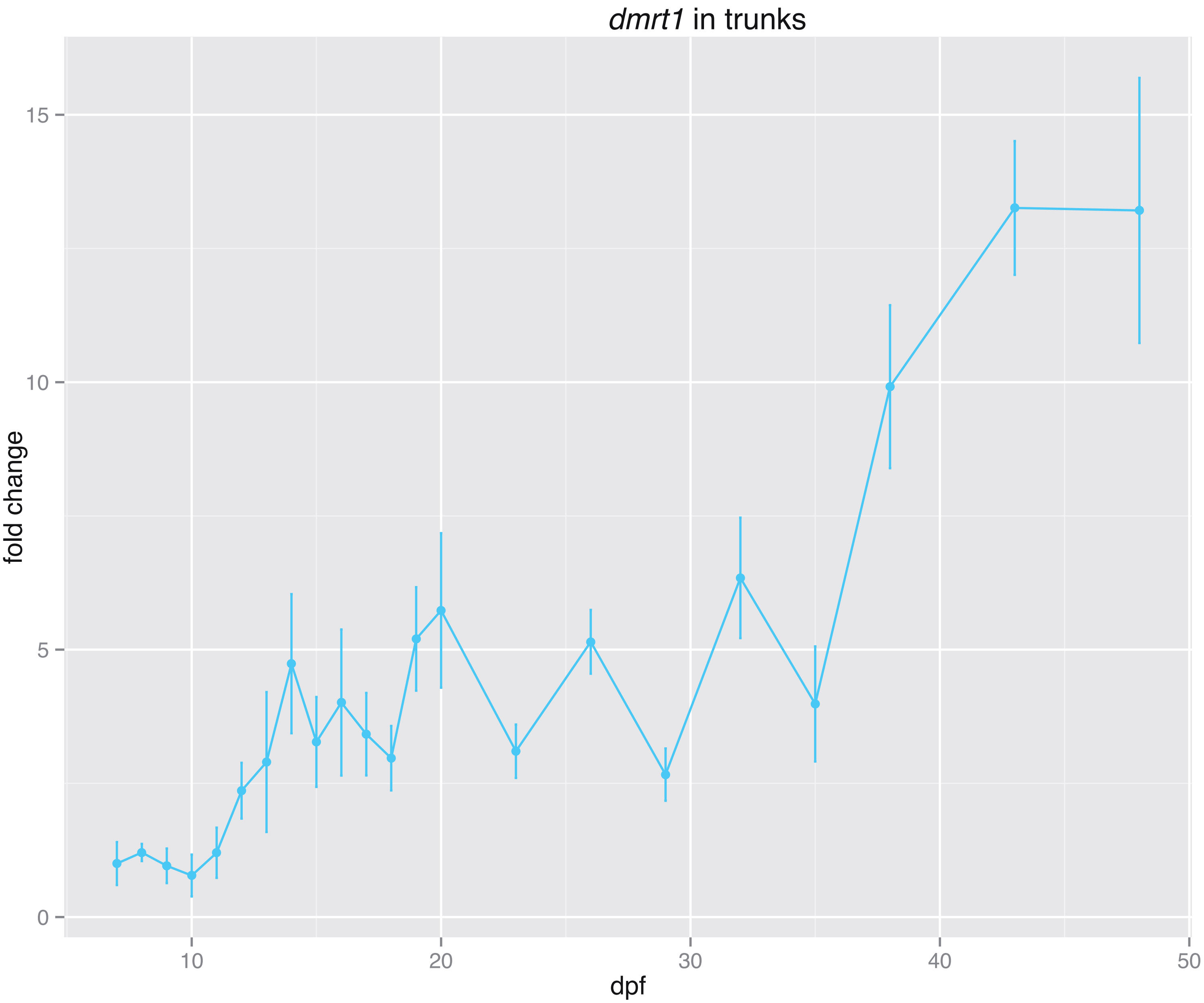

dmrt1 in trunks
fold change
dpf
0
5
10
15
10
20
30
40
50

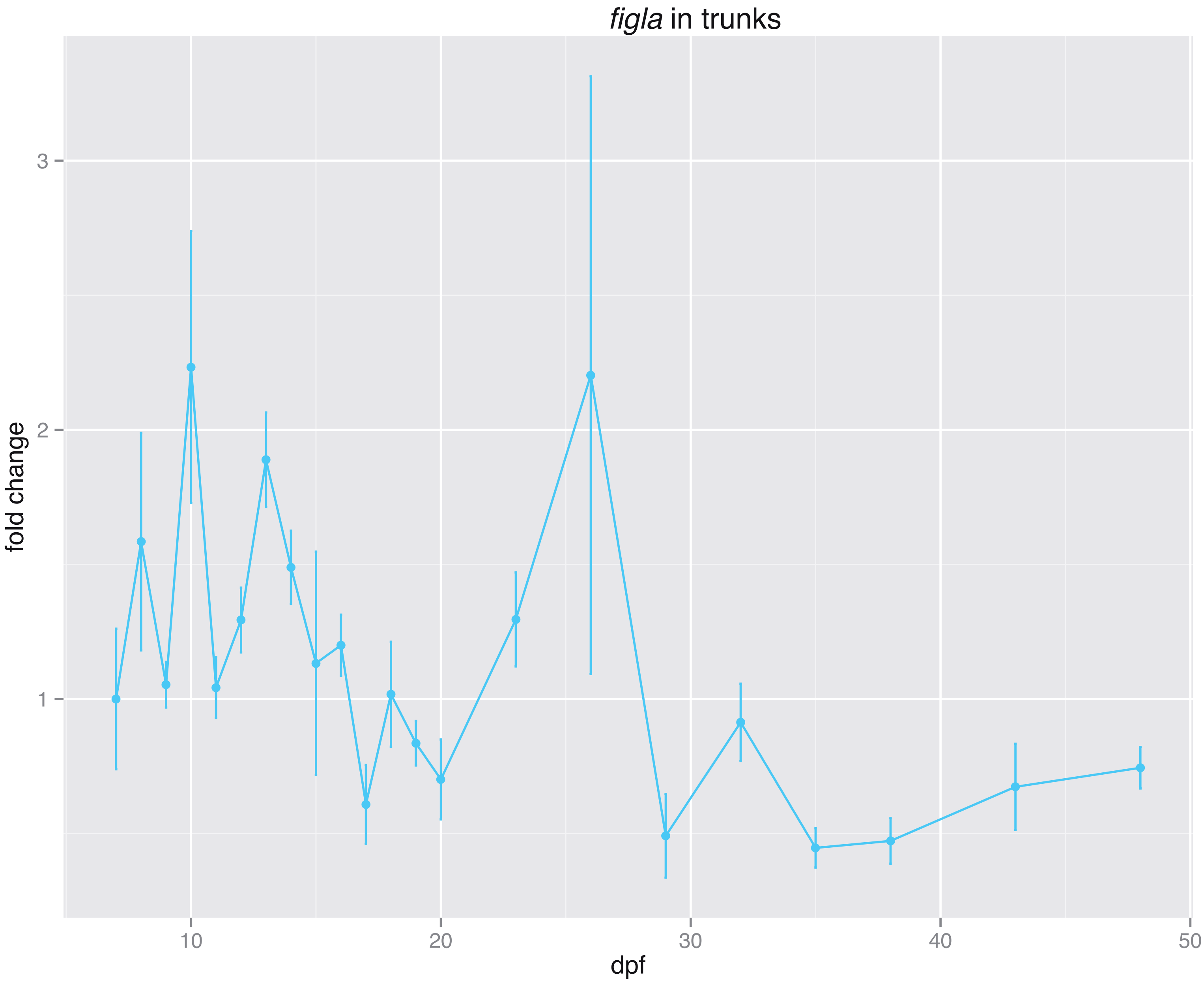

figla in trunks
fold change
dpf
1
2
3
10
20
30
40
50

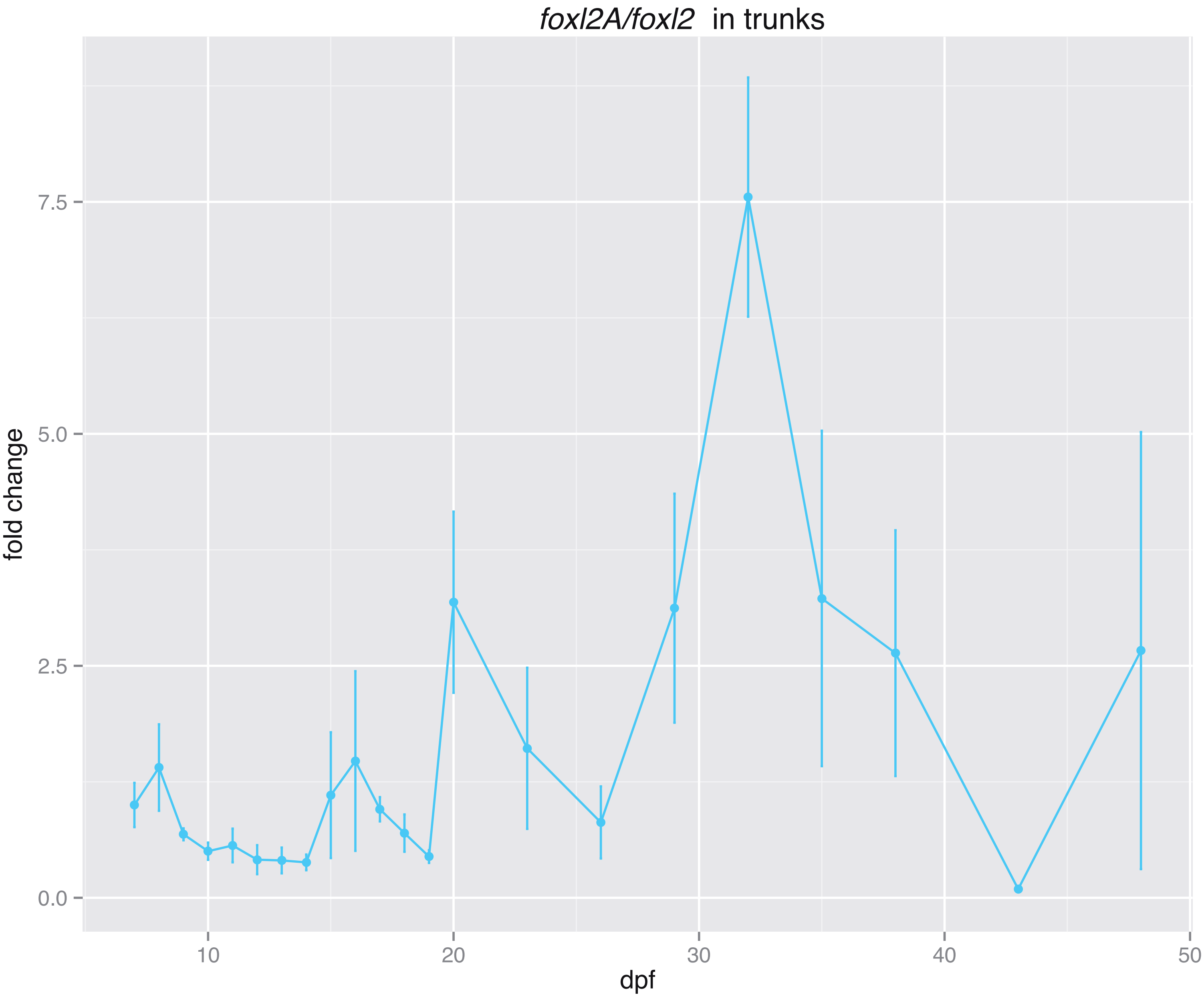

foxl2A/foxl2 in trunks
fold change
7.5
5.0
2.5
0.0
10
20
30
40
50
dpf

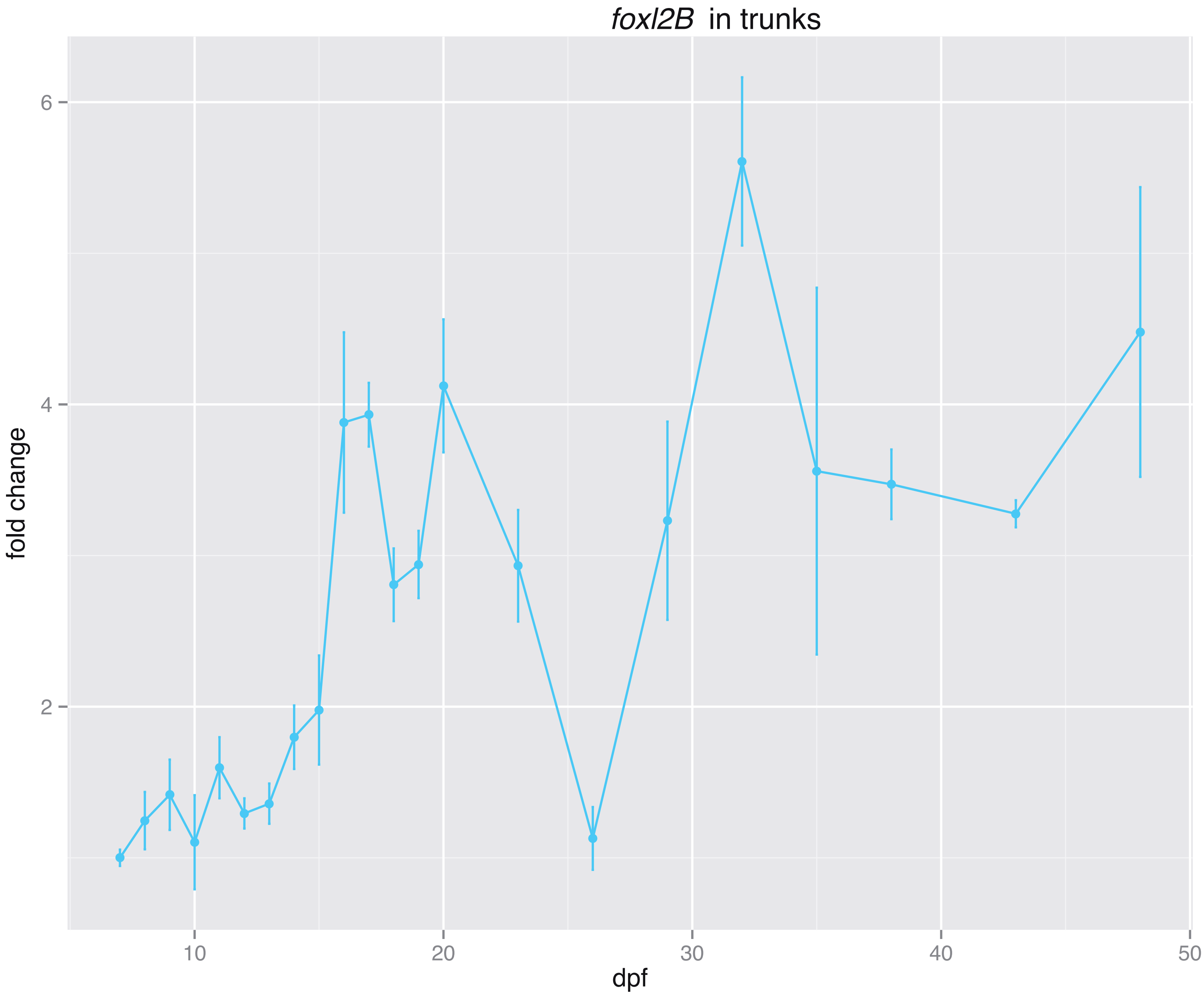

foxl2B in trunks
fold change
dpf
2
4
6
10
20
30
40
50

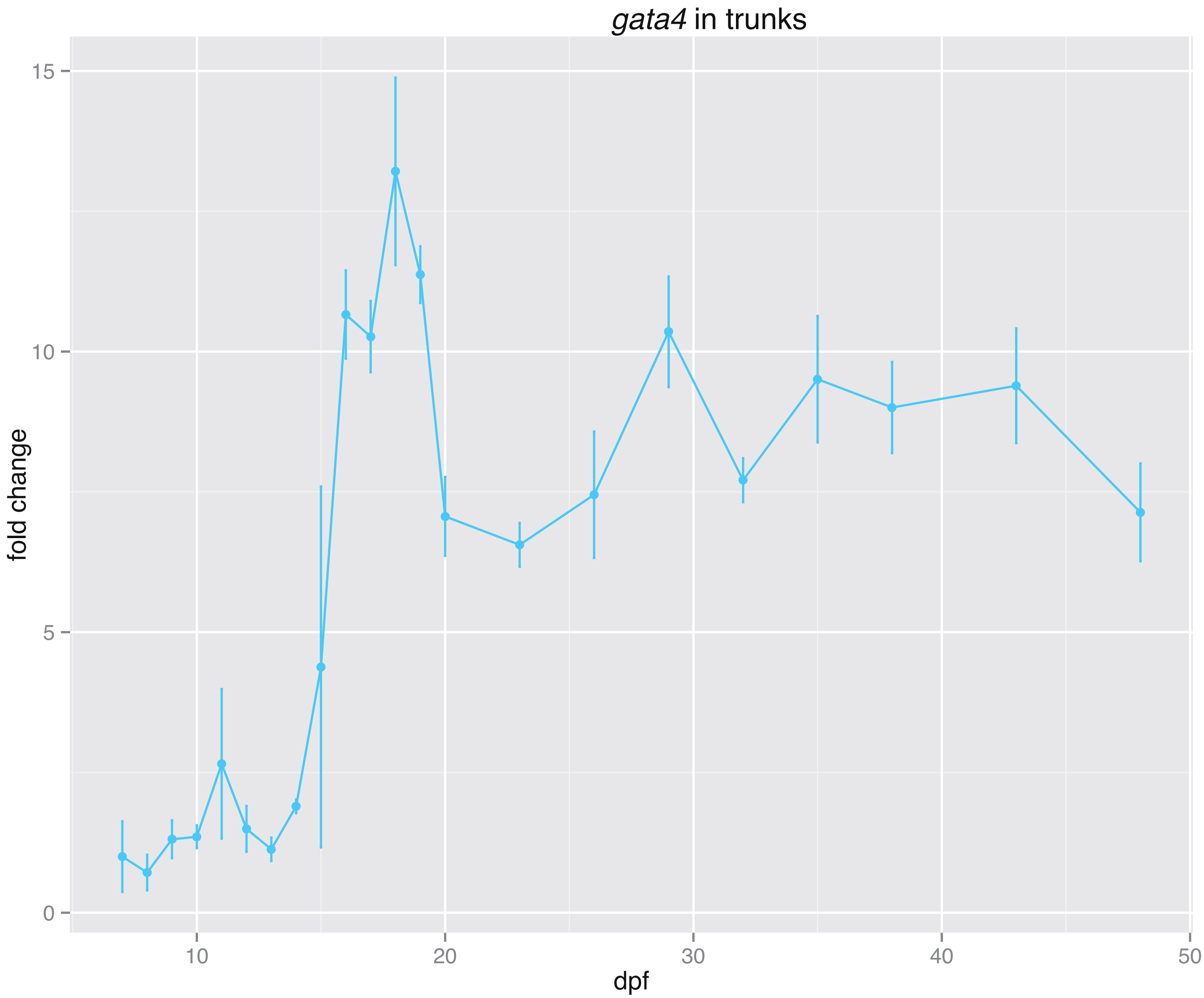

gata4 in trunks
fold change
dpf
0
5
10
15
10
20
30
40
50

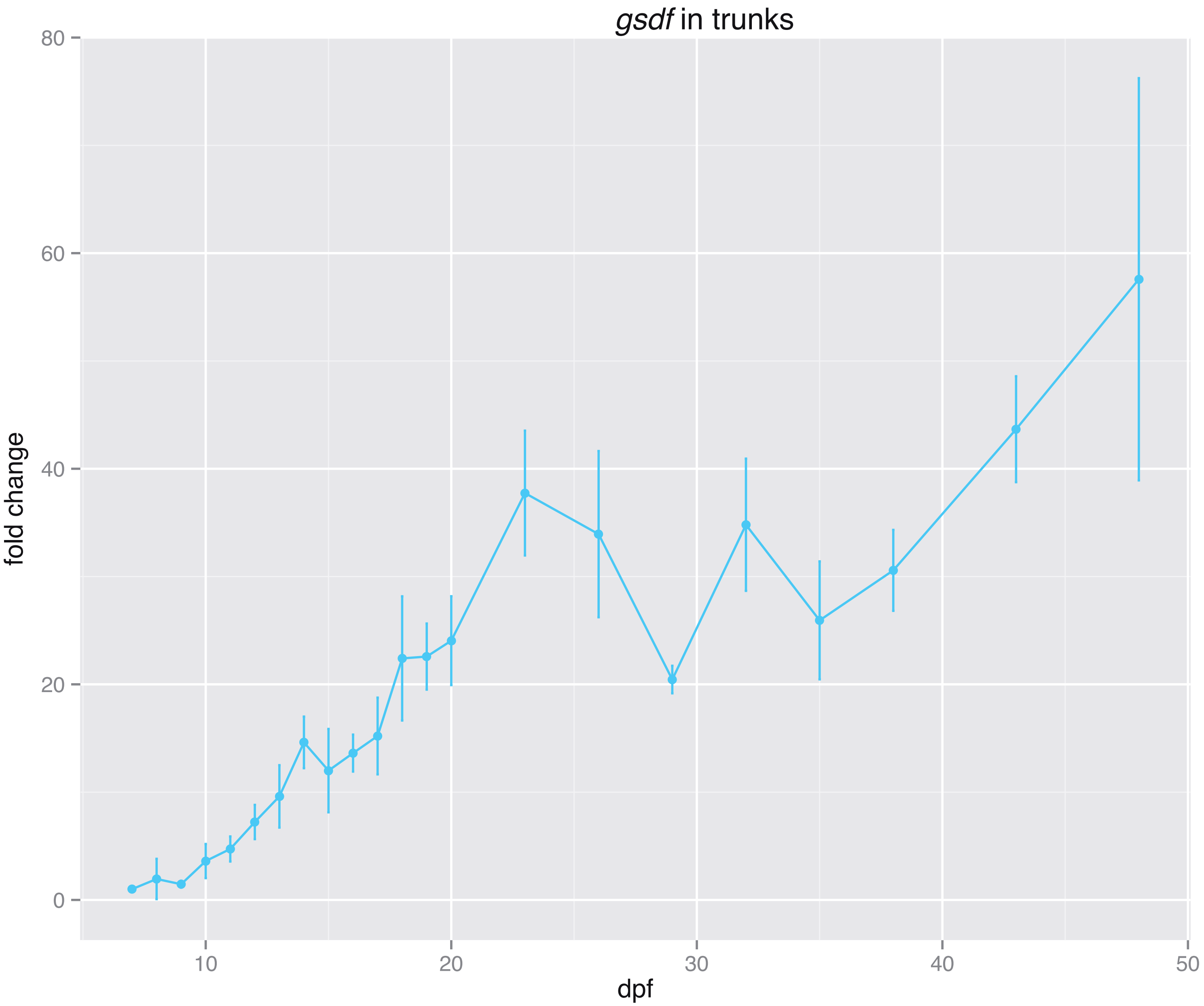

gsdf in trunks
80
60
40
20
0
fold change
10
20
30
40
50
dpf

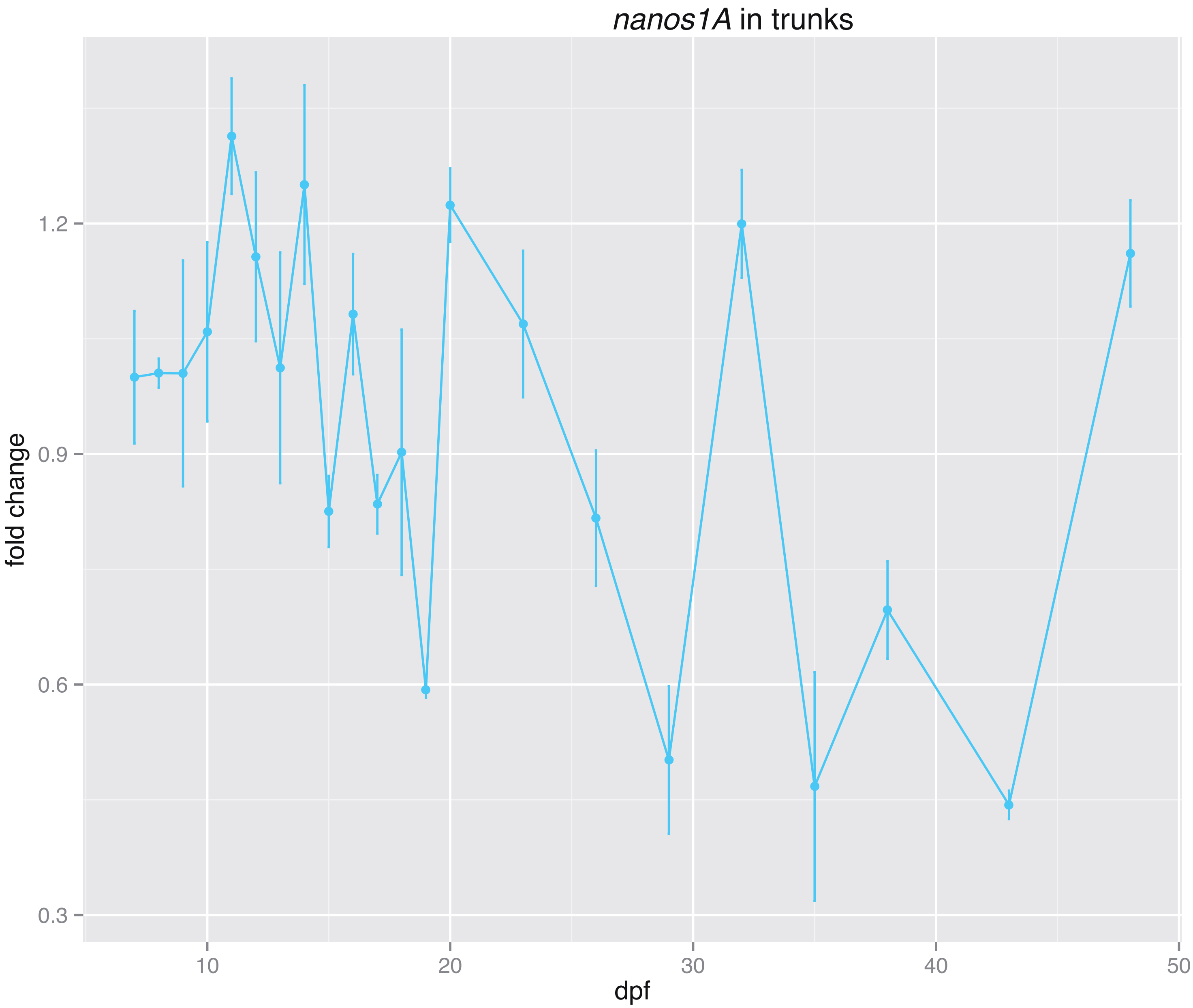

nanos1A in trunks
fold change
1.2
0.9
0.6
0.3
10
20
30
40
50
dpf

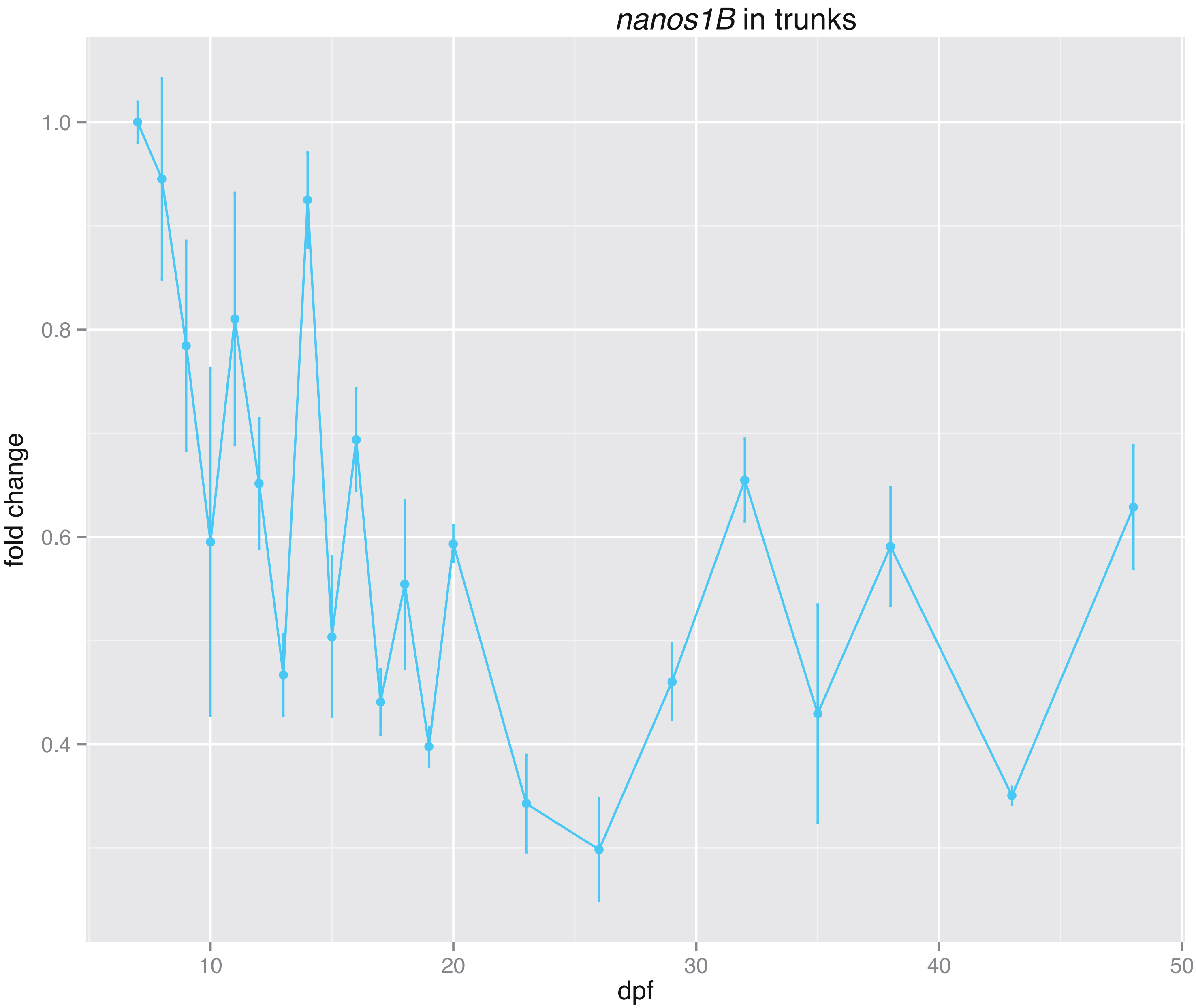
nanos1B in trunks
fold change
1.0
0.8
0.6
0.4
10
20
30
40
50
dpf

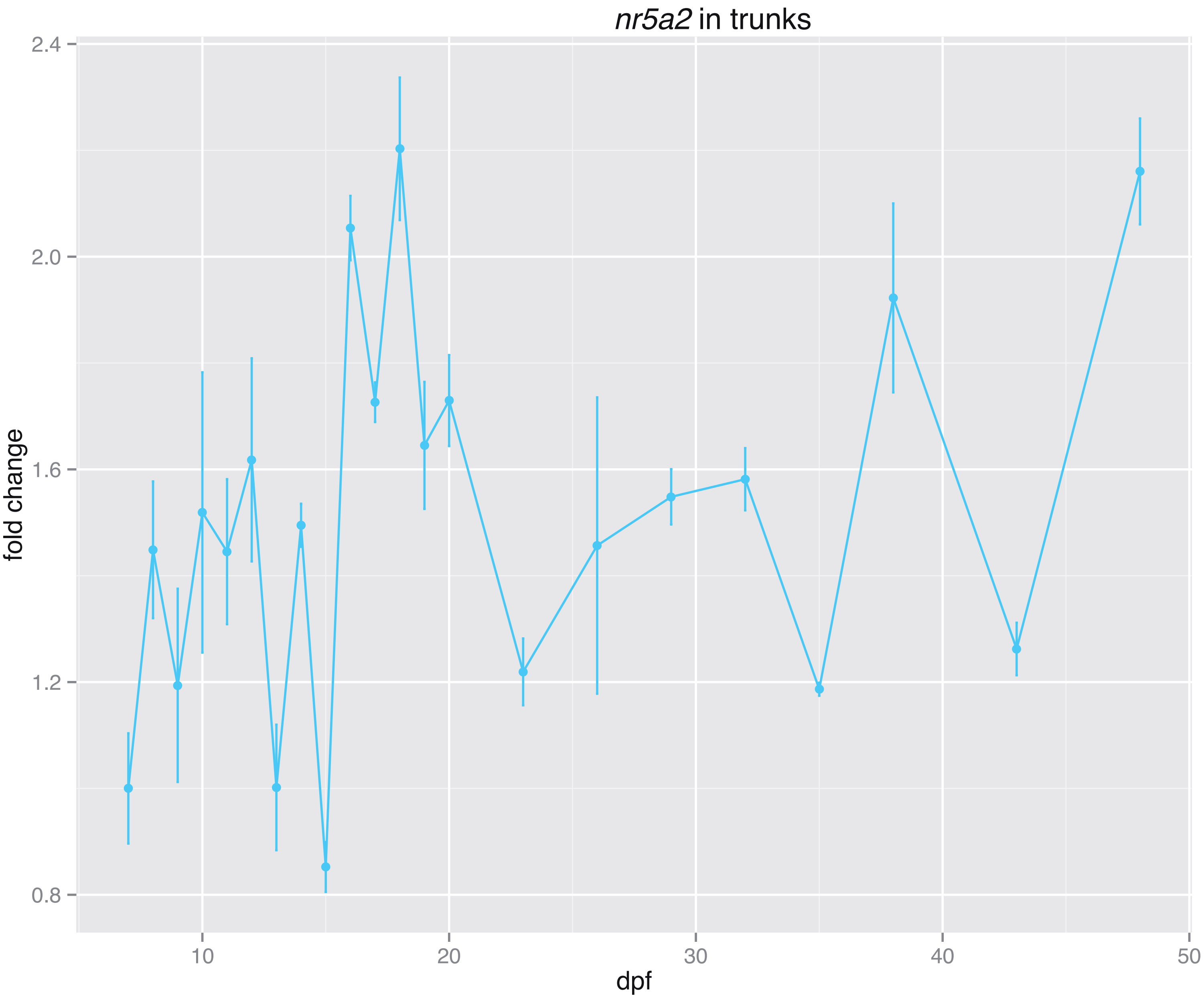
nr5a2 in trunks
2.4
2.0
1.6
1.2
0.8
fold change
10
20
30
40
50
dpf

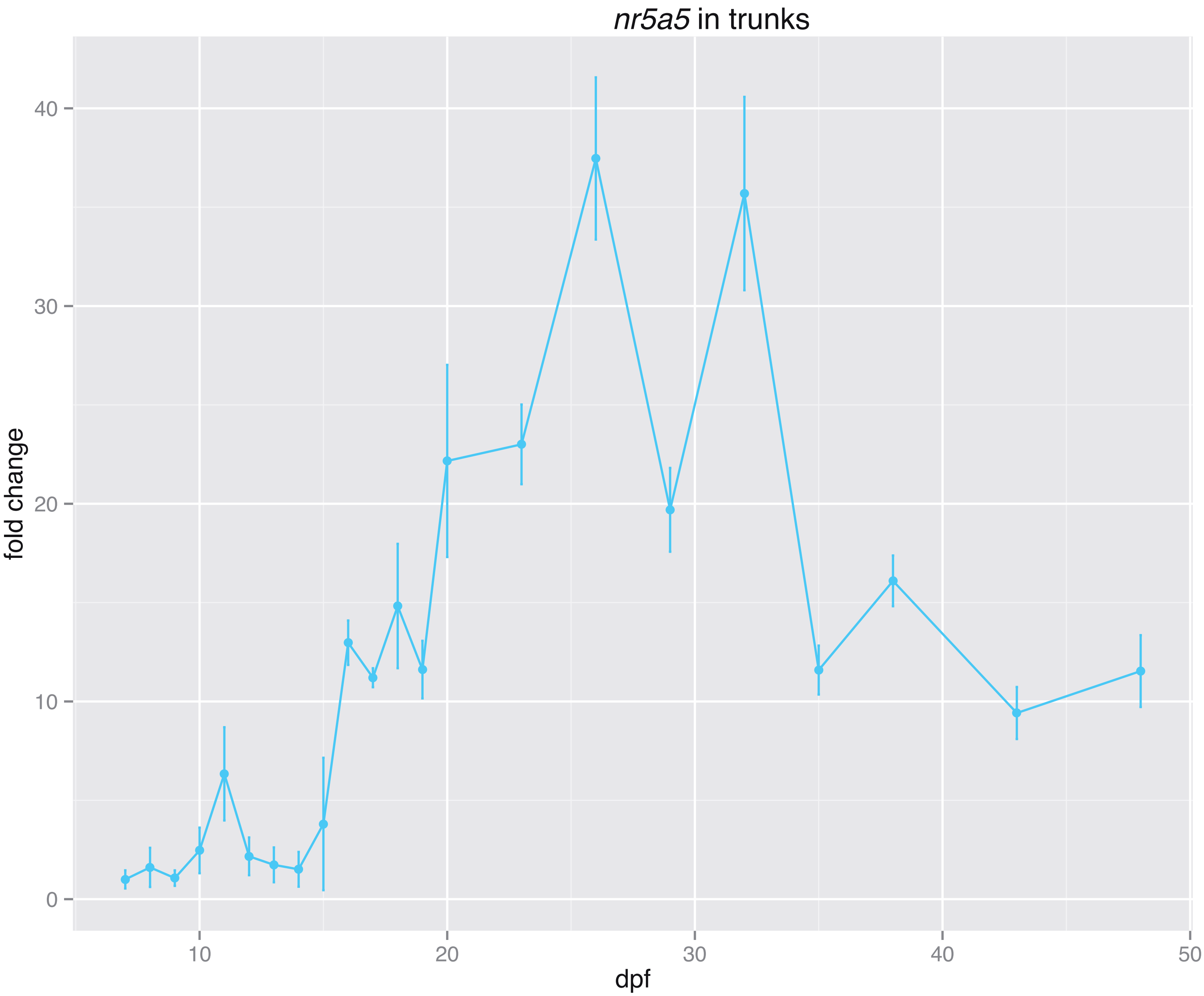

nr5a5 in trunks
fold change
dpf
0
10
20
30
40
10
20
30
40
50

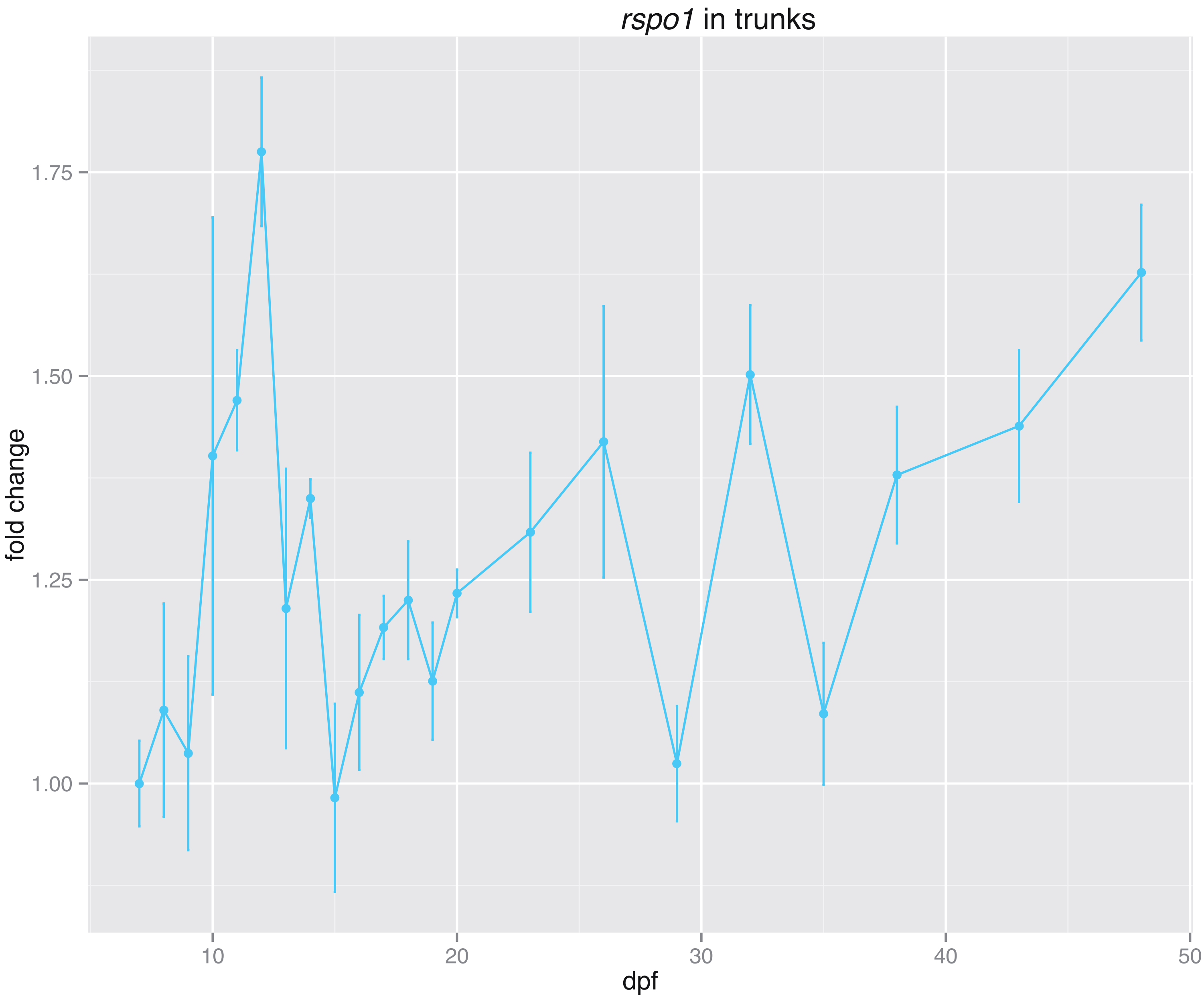

rspo1 in trunks
fold change
1.75
1.50
1.25
1.00
dpf
10
20
30
40
50

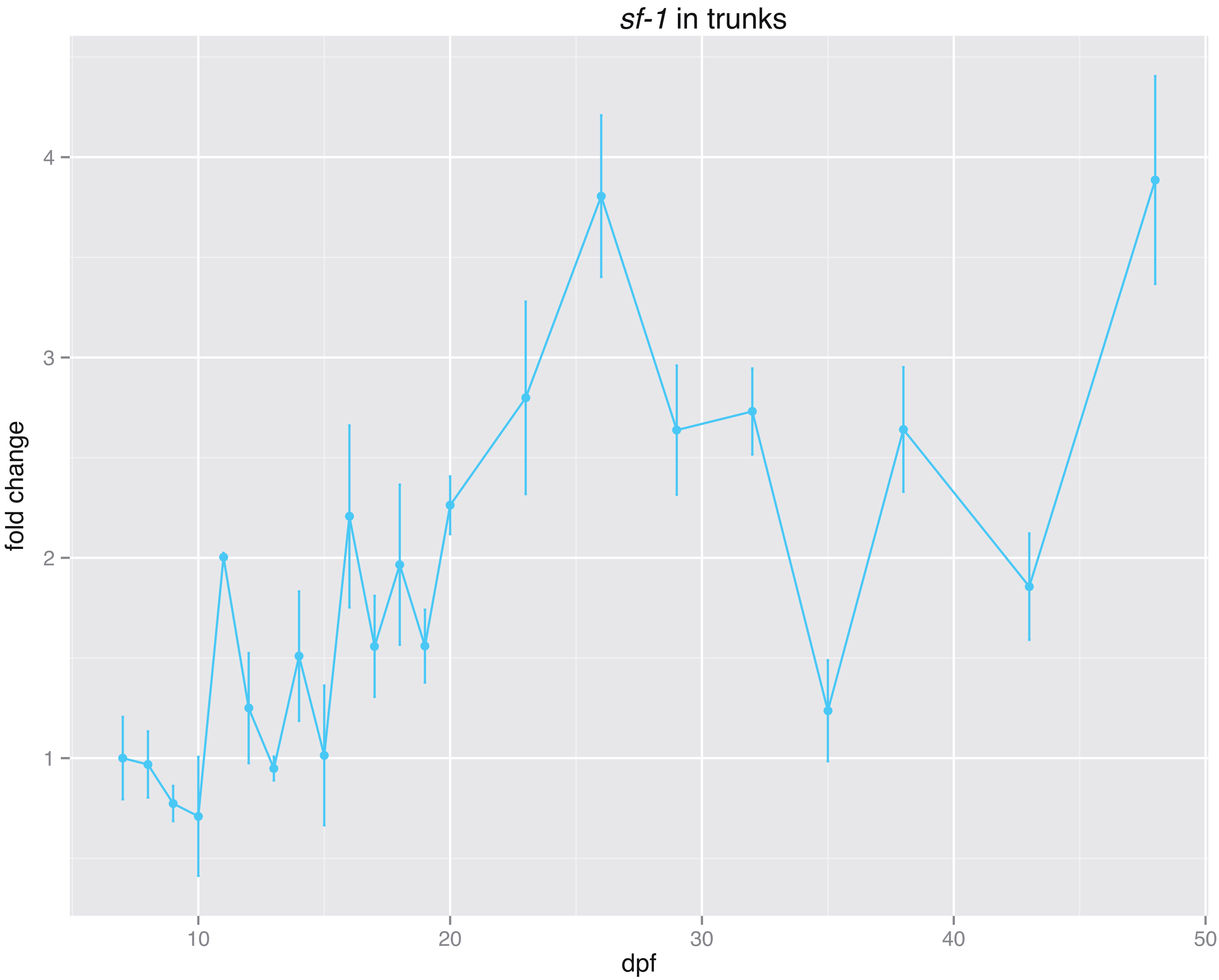

sf-1 in trunks
fold change
4
3
2
1
10
20
30
40
50
dpf

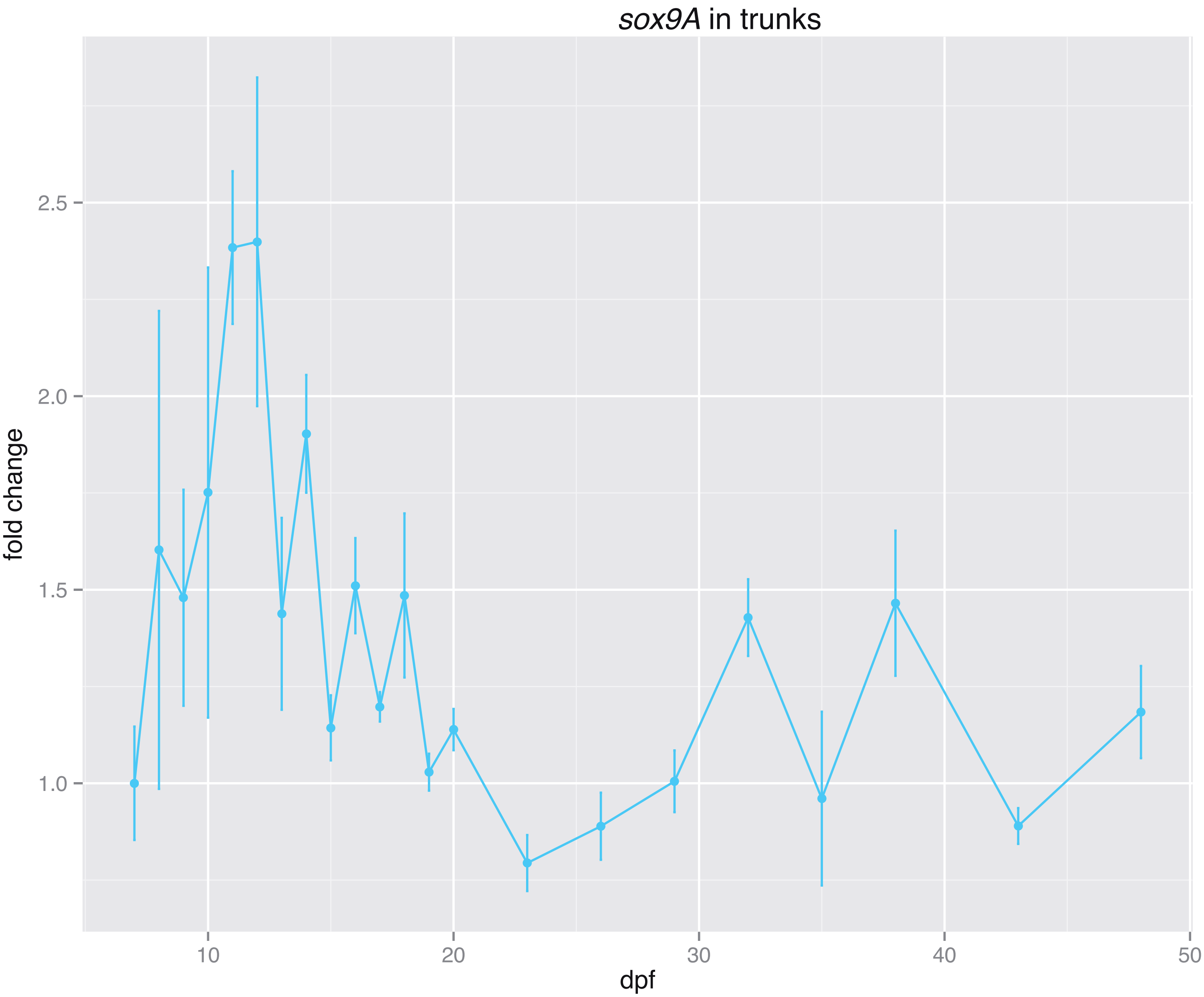

sox9A in trunks
fold change
dpf
2.5
2.0
1.5
1.0
10
20
30
40
50

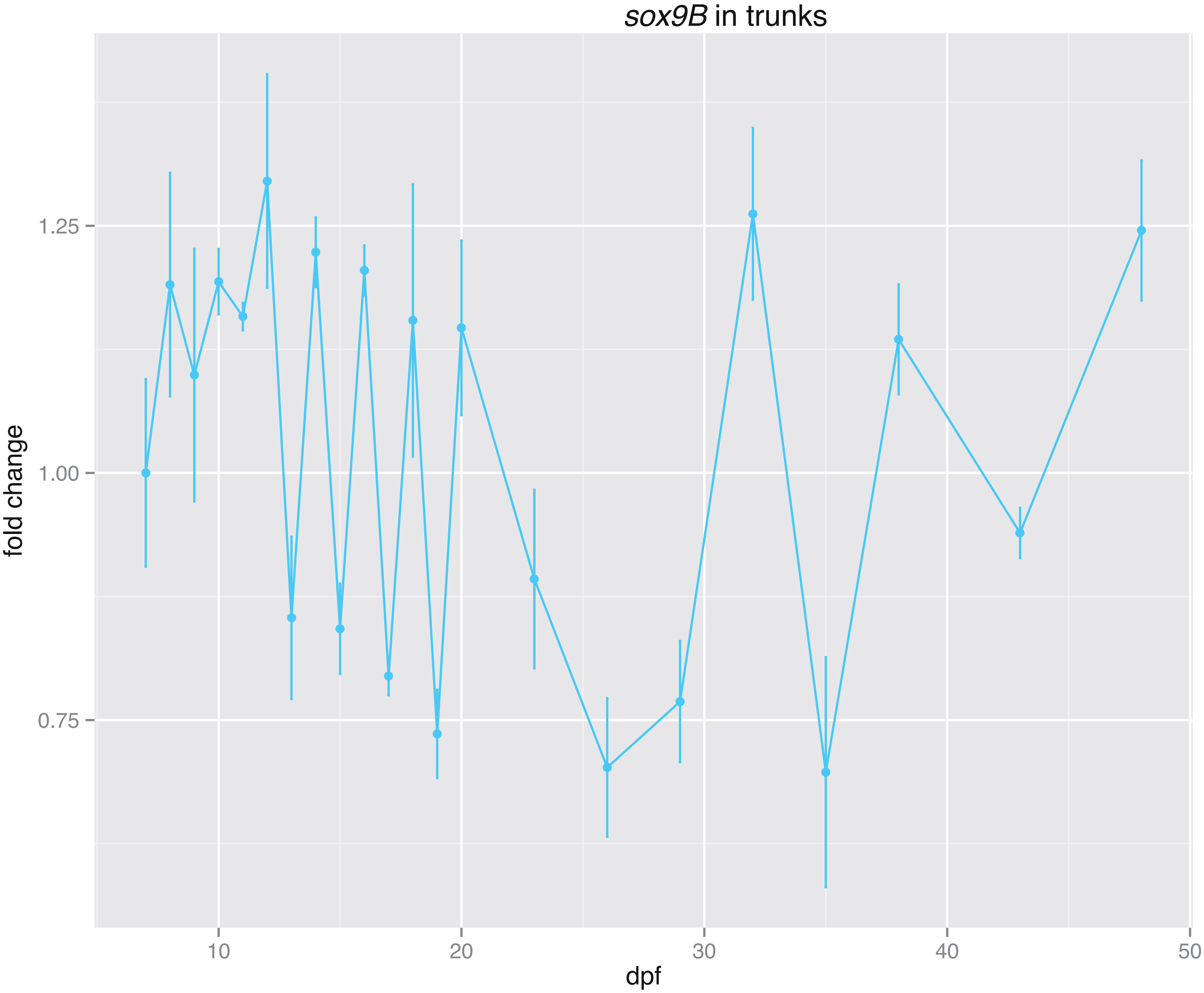

sox9B in trunks
fold change
1.25
1.00
0.75
10
20
30
40
50
dpf

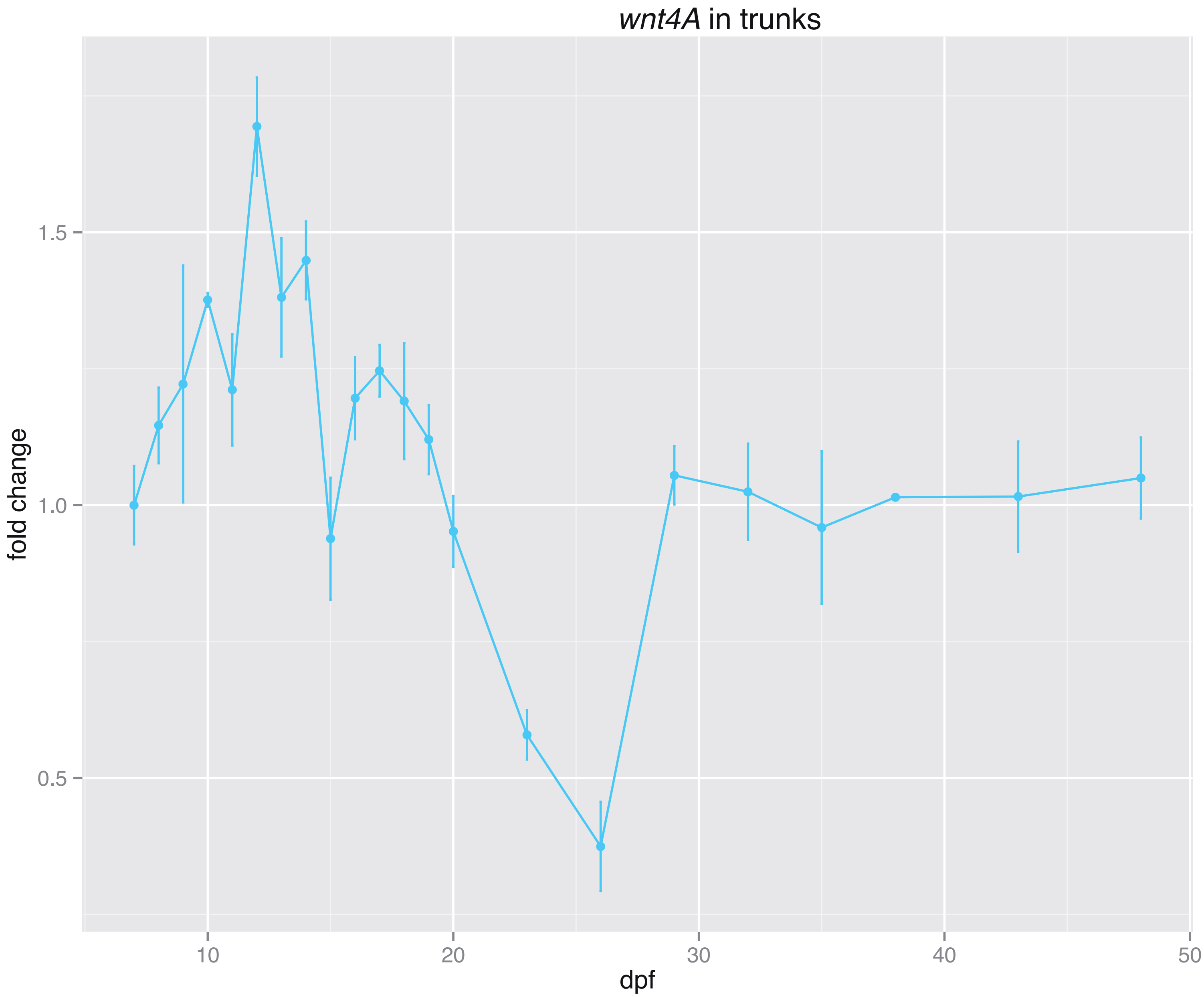

wnt4A in trunks
1.5
1.0
0.5
fold change
10
20
30
40
50
dpf

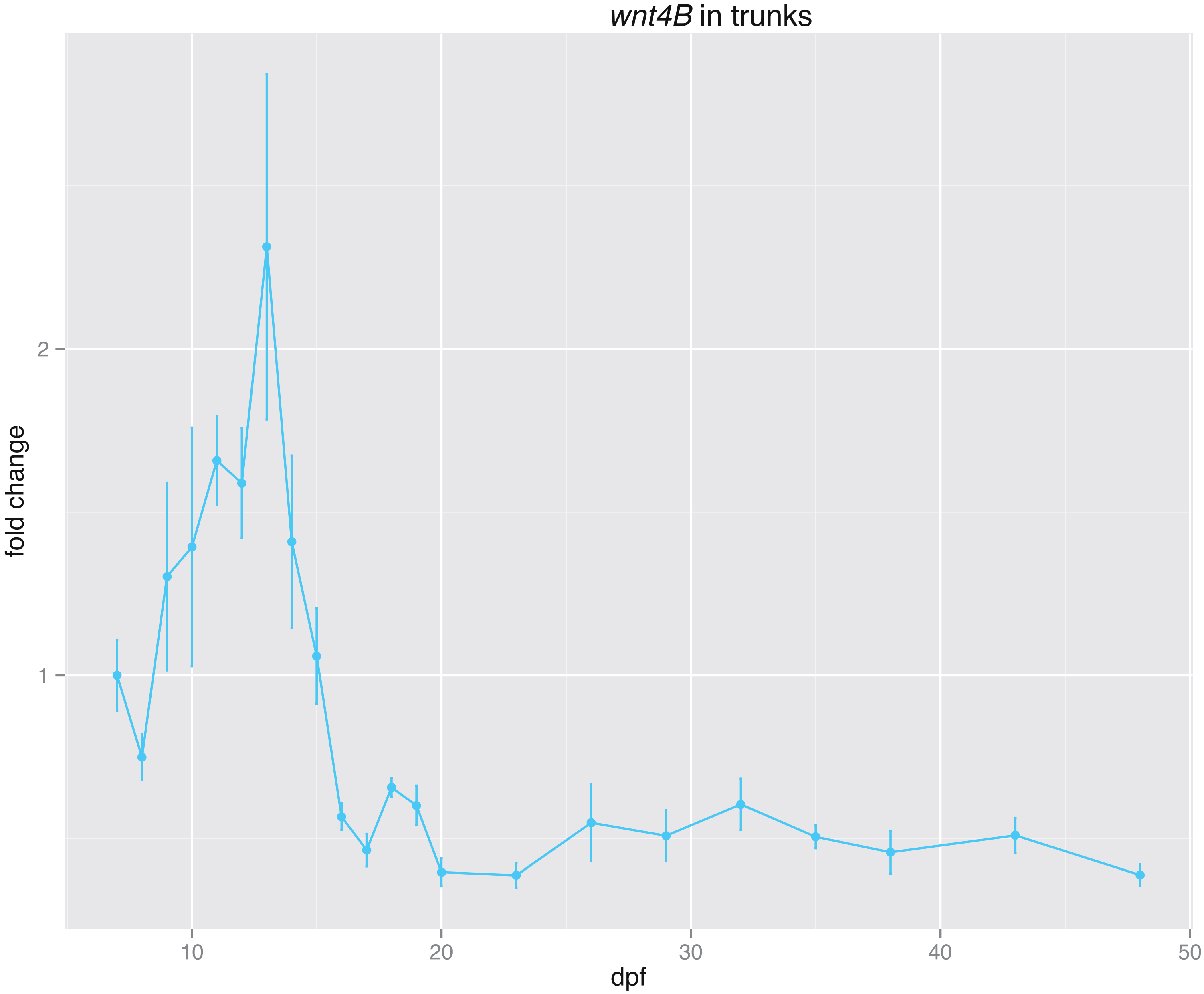

wnt4B in trunks
fold change
2
1
10
20
30
40
50
dpf

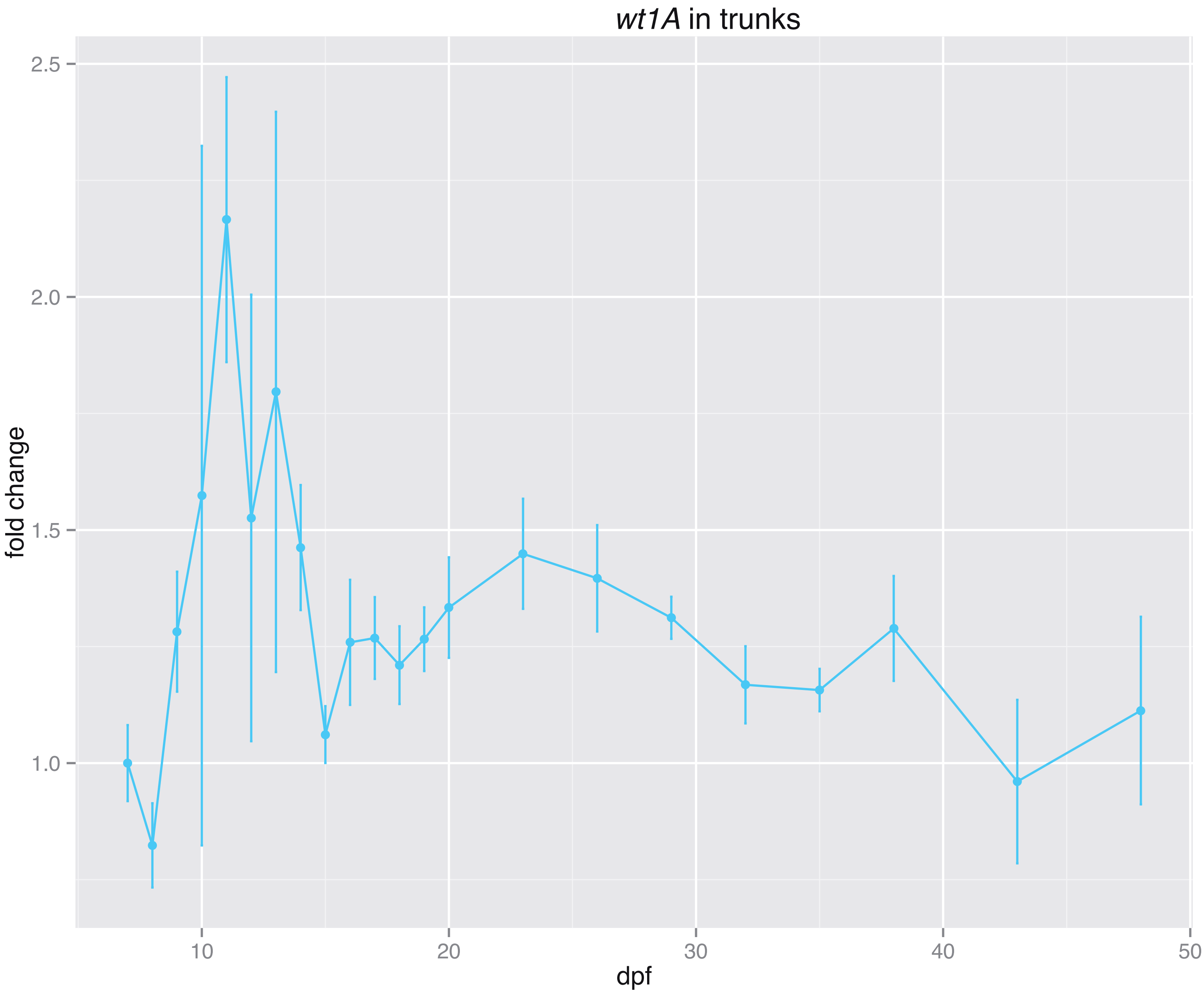

wt1A in trunks
fold change
2.5
2.0
1.5
1.0
dpf
10
20
30
40
50

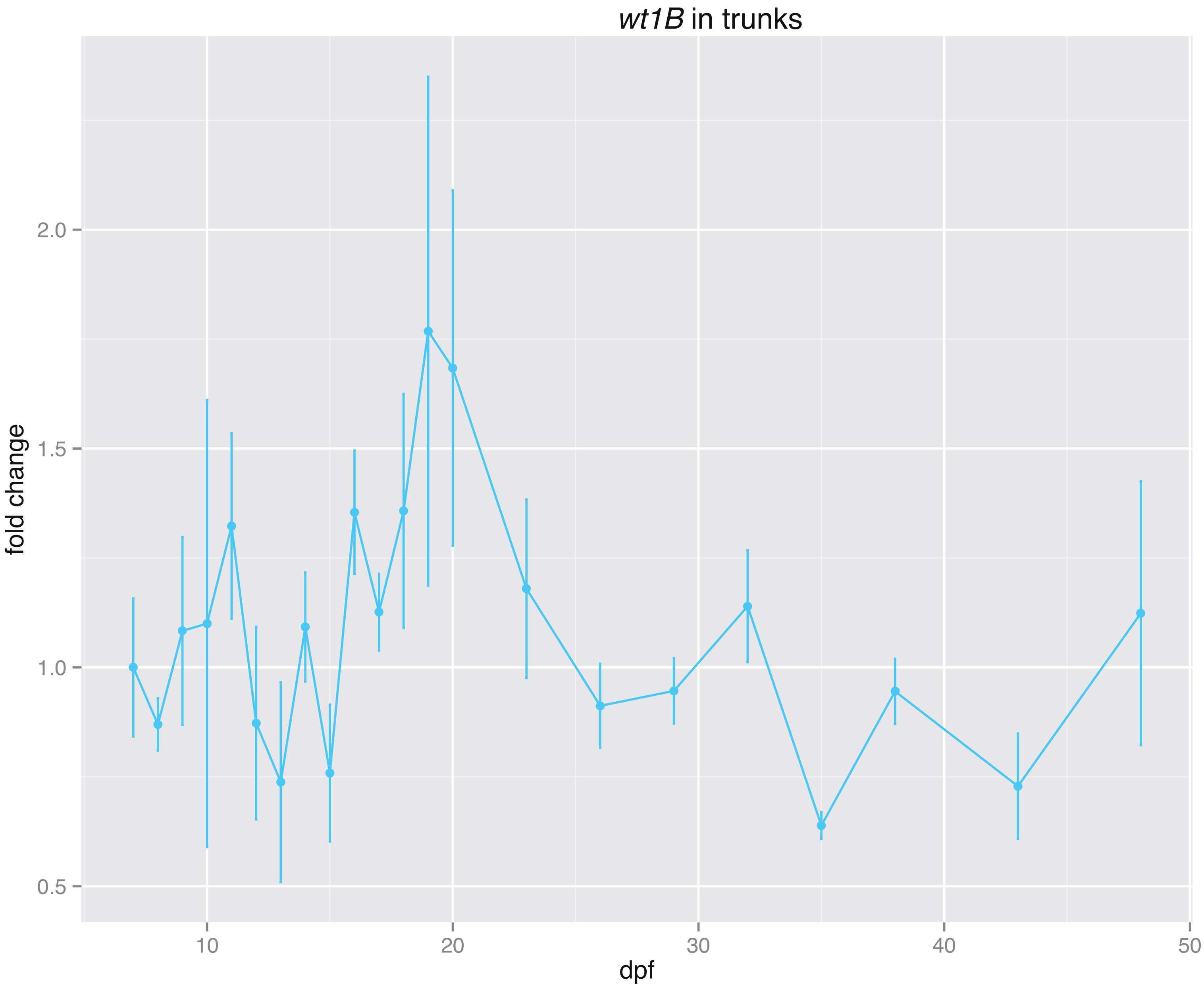

wt1B in trunks
fold change
2.0
1.5
1.0
0.5
dpf
10
20
30
40
50
